# Integrative proteomics defines FANCA as a multifunctional hub linking DNA repair to translation

**DOI:** 10.1016/j.isci.2026.117461

**Published:** 2026-09-09

**Authors:** Virgile Gibert, Frédérique Maczkowiak-Chartois, Benedetta Mancini, Emilie-Fleur Gautier, Johanna Bruce, Morgane Le Gall, Serge Urbach, Angelos Constantinou, Anne Helbling-Leclerc, Jihane Basbous, Filippo Rosselli

**Affiliations:** 1 Université Paris-Saclay, Gustave Roussy, CNRS, 94805 Villejuif, France; 2 Proteom'IC Facility, Université Paris Cité, CNRS, INSERM, Institut Cochin, 75014 Paris, France; 3 Bioinformat'IC Facility, Université Paris Cité, CNRS, INSERM, Institut Cochin, 75014 Paris, France; 4 IGF, Université Montpellier, CNRS, 34094 Montpellier, France; 5 IGH, Université Montpellier, CNRS, 34094 Montpellier, France; 6 ENS Paris-Saclay, 91190 Gif-sur-Yvette, France; 7 Equipe Labellisée Ligue Nationale Contre le Cancer, Villejuif, France

**Keywords:** fanconi anemia, DNA damage response, DNA repair, replication stress, spliceosome, ribosome, interactome

## Abstract

Fanconi anemia (FA) is a genome instability syndrome caused by defects in the FANC/BRCA DNA repair pathway, yet its broad clinical heterogeneity suggests functions beyond DNA repair. Using an integrative interactomics strategy combining endogenous co-immunoprecipitation, proximity-dependent labeling, *in silico* network integration, and functional analyses, we construct an interaction score-based multilayered FANCA protein-protein interaction landscape. Beyond its association with the FANCcore complex and DNA repair machineries, FANCA engages with proteins involved in chromatin remodeling, RNA metabolism, and ribosome biogenesis. Functional analyses reveal ribosome-related processes as a robust feature of FANCA-associated networks. Quantitative analyses further reveal alterations in ribosome protein stoichiometry, suggesting defective ribosome biogenesis. Our findings suggest translation alterations may explain the pleiotropic clinical manifestations of FA by posing FANCA as a link between genome maintenance, ribosome biogenesis, and translation. Our study highlights how integrative interaction allows deciphering regulatory roles of DNA repair proteins in cellular homeostasis and disease.

## Highlights

• FANCA interactomics reveals a multilayered network beyond DNA repair
• FANCA associates with ribosome biogenesis and translation networks
• FANCA loss alters ribosome composition and polysome organization

## Graphical abstract

**Figure:**
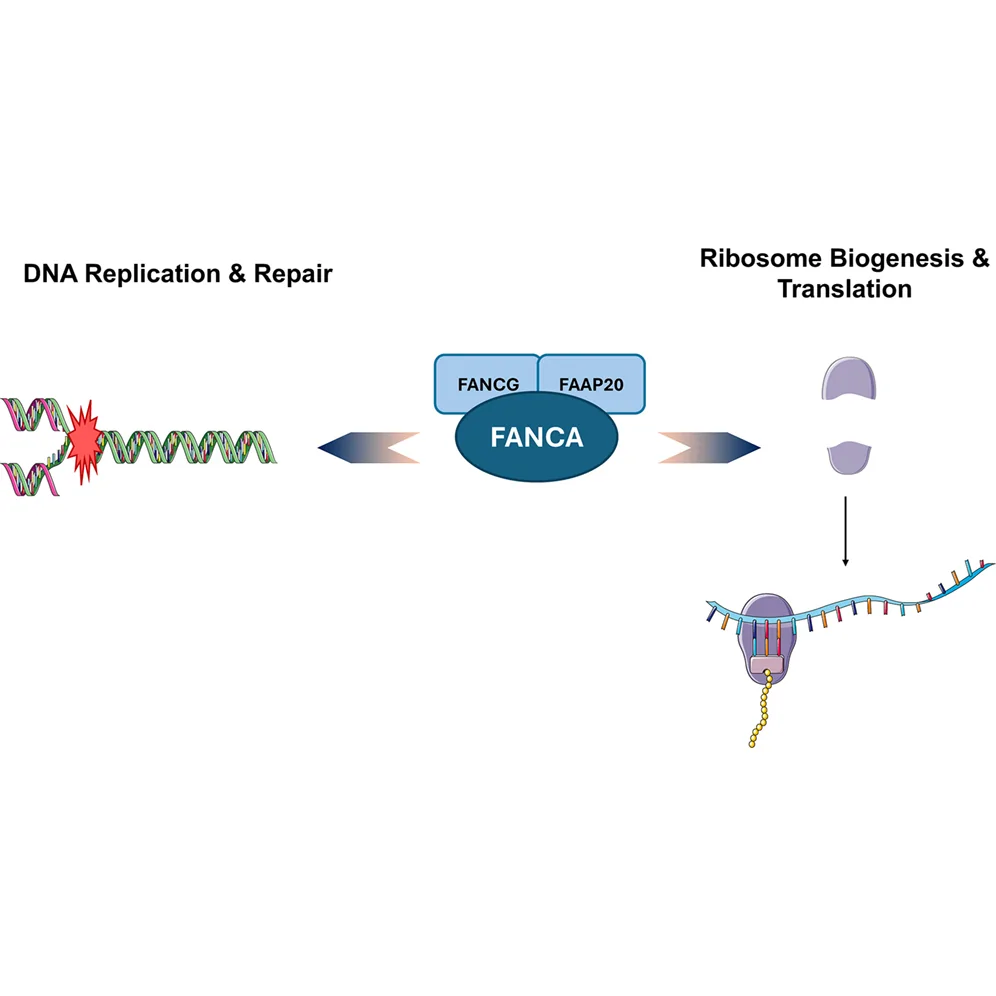

## Teaser

Genomics; Molecular biology; Cell biology

## Introduction

Fanconi anemia (FA) is a genetically heterogeneous disorder characterized by bone marrow failure, developmental abnormalities, and a high predisposition to cancer. Although FA is classically defined as a genome instability syndrome caused by defects in the FANC/BRCA DNA repair pathway, the breadth of its clinical manifestations suggests that additional cellular processes are affected. In particular, the severity of hematopoietic failure and developmental defects in FA patients cannot be fully explained by impaired DNA interstrand crosslink repair alone.^1^^,^^2^^,^^3^^,^^4^^,^^5^

At the molecular level, FANC proteins assemble into functional modules that coordinate replication stress responses and homologous recombination-mediated DNA repair within the FANC/BRCA pathway.^6^ FANCA, the most frequently mutated FA gene, plays a central role in this pathway as part of the FANCcore complex, which promotes FANCD2/FANCI monoubiquitination and recruitment of downstream repair factors. Disruption of this pathway leads to hypersensitivity to DNA crosslinking agents, replication fork instability, chromosomal abnormalities, as well as alterations in R-loop homeostasis, telomere maintenance, and mitotic progression.^7^^,^^8^^,^^9^^,^^10^^,^^11^^,^^12^ However, accumulating evidence indicates that FANCA functions extend beyond canonical DNA repair reactions.

Recent studies have implicated FANCA and other FA proteins in diverse cellular processes, including mitochondrial homeostasis,^13^^,^^14^ inflammatory signaling,^15^^,^^16^^,^^17^^,^^18^^,^^19^ and proteostasis.^4^^,^^20^^,^^21^ Notably, FANCA has been linked to nucleolar integrity and ribosome biogenesis, and FANCA deficiency is associated with reduced global translation rates.^20^ These observations raise the possibility that FANCA contributes more broadly to the coordination of genome maintenance with gene expression and protein synthesis, yet the molecular basis for these non-canonical functions remains poorly understood.

Protein-protein interaction (PPI) networks provide a powerful framework to uncover multifunctional roles of regulatory proteins. However, traditional interactomics approaches often emphasize highly stable interactions, potentially overlooking weaker or context-dependent associations that may nonetheless be biologically relevant. We hypothesized that FANCA operates within a broader interaction landscape that integrates DNA repair with other aspects of genetic information processing, including RNA metabolism and translation.

To test this hypothesis, we combined endogenous co-immunoprecipitation (coIP), proximity-dependent biotinylation, and integrative *in silico* analyses to reconstruct a comprehensive FANCA protein-protein interaction network. By systematically analyzing interaction selectivity and functional convergence across multiple experimental platforms and cell types, we uncover an extended FANCA interactome that links genome maintenance to ribosome biogenesis and translational control. Biological experiments further demonstrate that FANCA loss alters ribosome composition and polysome organization, positioning FANCA as a coordinator of DNA repair and protein synthesis. Together, these findings redefine FANCA as a multifunctional hub in cellular homeostasis and provide a framework to understand the pleiotropic manifestations of FA.

## Results

### Generation of FANCA co-immunoprecipitated proteins datasets

**Figure 1:**
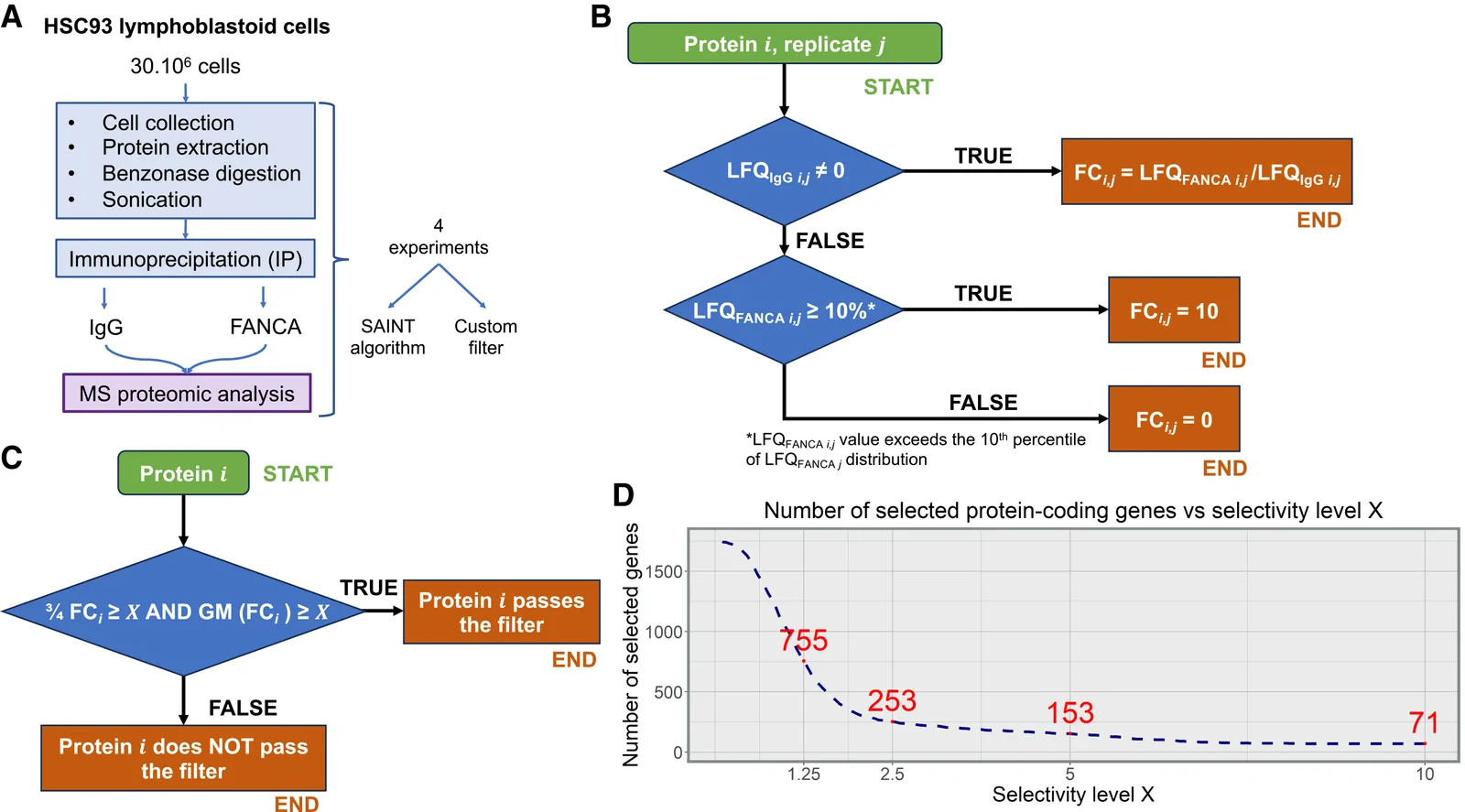
FANCA immunoprecipitation in HSC93 cells: Pipeline of analysis and data filtering (A) Schematic representation of our experimental and analytical procedure. (B) IP FANCA/IP IgG ratio calculation algorithm. (C) In-house interactor filter used with HSC93 IP data. (D) Number of genes passing the filter as a function of selectivity level *X* = 1.25, *X* = 2.5, *X* = 5, and *X* = 10.

To map the PPI network of FANCA, the endogenous protein was immunoprecipitated from whole-cell extracts of HSC93 lymphoblasts, an EBV-immortalized cell line expressing an intact FANC pathway. To minimize nucleic acid-mediated artifacts, cell extracts were treated with benzonase to digest RNA and DNA, followed by sonication, prior to immunoprecipitation (IP) with either an anti-FANCA antibody or an immunoglobulin G (IgG) isotype control. coIP proteins were subsequently identified and quantified using label-free quantitative (LFQ)-mass spectrometry (MS) (Figure 1A).

Across these experiments, MS detected between 720 and 3,234 proteins per IP, yielding a total of 3,420 unique proteins across all FANCA and control IPs (Table S1, sheet 1). LFQ values were calculated for each identified protein in each independent biological replicate. To prioritize candidate FANCA interactors, the data were analyzed using an in-house filtering strategy described below (Figure 1A).

For each protein *i* in each experiment *j*, a fold-change (FC) value was calculated as:

$${\text{FC}}_{i,j}=\frac{{\text{LFQ}}_{\text{FANCA},i,j}}{{\text{LFQ}}_{\text{IgG},i,j}}\text{.}$$

For proteins not detected in the IgG control (LFQ_IgG*,i*,j_ = 0), FC_***i***,j_ was set to 10, provided that LFQ_FANCA,*i*,j_ exceeds the 10^th^ percentile of LFQ_FANCA,j_ values in that experiment, otherwise FC _*i*,j_ was set to 0 (Figure 1B). To identify potential interactors, a protein was retained at a given selectivity threshold *X* if at least three out of four FC values exceeded *X* and if the geometric mean of all four FC values was also greater than *X*. The geometric mean criterion minimizes the influence of outliers and enhances selectivity (Figure 1C). Filtering was applied at four selectivity levels (*X* = 1.25, 2.5, 5.0, and 10), yielding lists containing 755, 253, 153, and 71 FANCA-associated proteins, respectively (Figure 1D; Table S1, Sheets 2–5; Table S2, sheet 1).

The original IP-MS data were also analyzed using SAINT (Significance Analysis of INTeractome), a probabilistic scoring algorithm specifically developed for IP-MS experiments to assess the likelihood of true biological interactions.^22^ This analysis yielded 58 FANCA-interacting proteins with a SAINT probability (SP) > 0.95 (Figure S1A; Table S1, sheet 6; Table S2, sheet 1). These proteins were combined with those identified at the highest selectivity level (*X* = 10) in our in-house filtering pipeline, resulting in a high-selectivity dataset of 93 unique FANCA interactors (Table S2, sheet 1).

**Figure 2:**
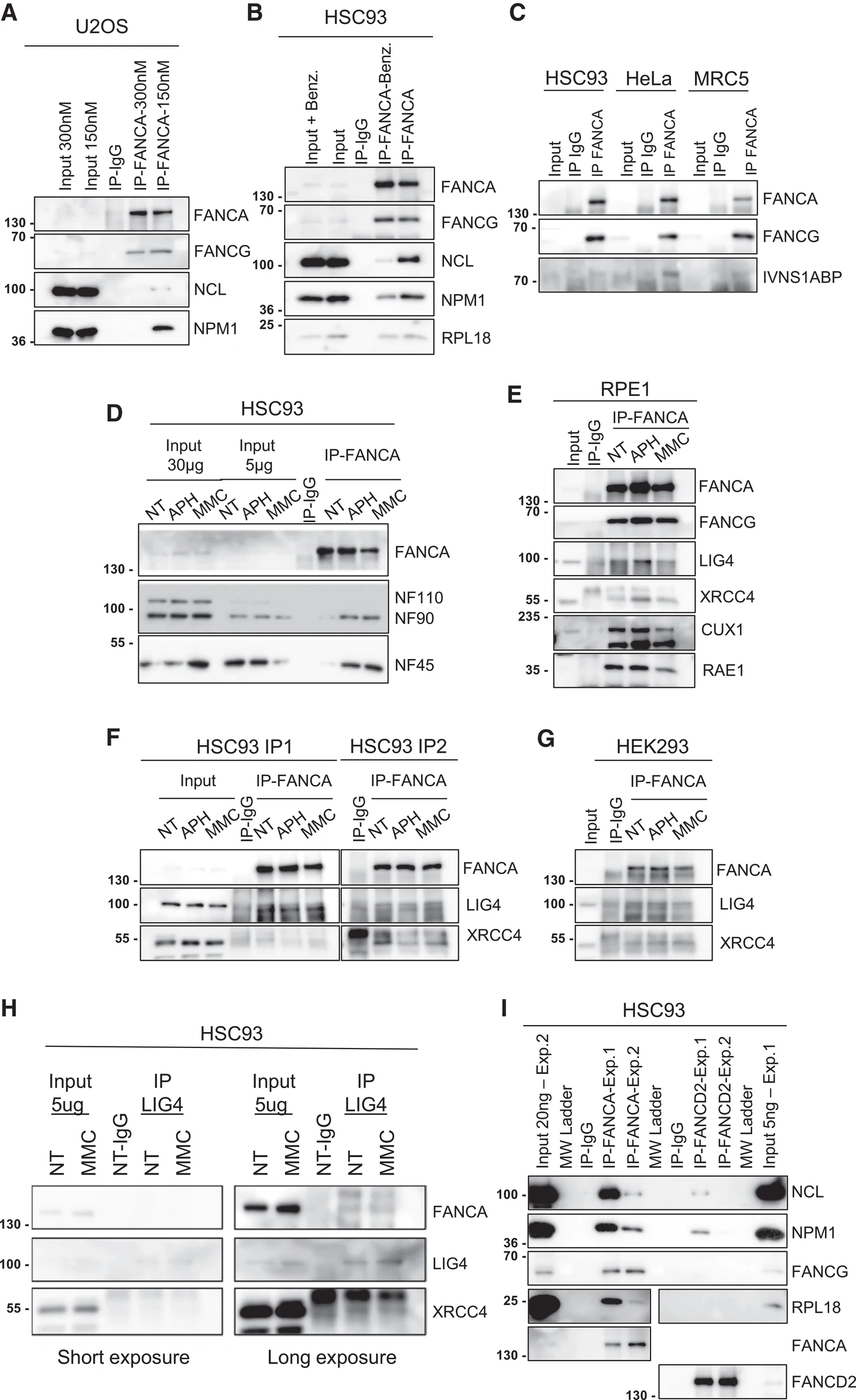
Validation of the FANCA coIP (A–C) FANCA coIP FANCG, NCL, NPM1, RPL18, or INVS1ABP in U2OS, HSC93, HeLa, and/or MRC5 cells. (D–G) FANCA coIP ILF2/NF45 and ILF3/NF90, FANCG, LIG4, XRCC4, CUX1, and RAE1 in unstressed conditions as well as in response to mitomycin C (MMC, 200 ng/ml, 16–24h) or aphidicolin (APH, 0.6 μM, 16–24h) in HSC93, RPE1 and/or HEK293 cells. (H) LIG4 coIP XRCC4 and FANCA in both untreated and MMC treated FANCA-proficient HSC93 cells (MMC, 200 ng/ml, 16–24h). (I) CoIP of NCL, NPM1 FANCG, and RPL18 with FANCA or FANCD2 in HSC93 cells. Two experiments with similar outcomes were reported. Please refer to Data S1 for the original uncropped blots showing the molecular weight (MW) scale and expected MW of the analyzed proteins, as well as images taken at different exposure times.

Before proceeding with *in silico* functional analyses and reconstruction of the FANCA PPI landscape, FANCA IPs were repeated using extracts from HSC93, HeLa, U2OS, HEK293, and RPE1 cell lines under varying experimental conditions (150 nM vs. 300 nM salt; with or without benzonase; exposure to mitomycin C [MMC] or aphidicolin [APH]). coIP proteins were analyzed by immunoblotting to validate selected interactions (Figure 2). These experiments successfully recovered FANCG, CUX1, IVNS1ABP, RAE1, and LIG4 (*X* = 10 dataset); RPL18 and XRCC4 (*X* = 5 dataset); ILF2(NF45) (*X* = 2.5 dataset); and, ILF3(NF90), NPM1, and NCL (*X* = 1.25 dataset) (Figures 2A–2I). The interaction between FANCA and FANCG was resistant to salt concentration (Figure 2A) and to benzonase treatment (Figure 2B). In contrast, interactions with NCL and NPM1 were lost at high salt concentration and were diminished, though still detectable, following benzonase treatment (Figures 2A and 2B). The FANCA-RPL18 coIP appeared to be resistant to benzonase treatment (Figure 2B). Upon genotoxic stress, exposure to MMC or APH, the FANCA-CUX1, FANCA-RAE1, and FANCA-LIG4/XRCC4 interactions persisted or even increased as observed for the FANCA-ILF2(NF45)/ILF3(NF90) coIP (Figures 2D–2G). The FANCA-LIG4/XRCC4 interaction was further validated by reverse IP, whereby FANCA and XRCC4 were recovered following LIG4 IP in both untreated and MMC-treated conditions (Figure 2H). Finally, to assess IP specificity, the coIP of NPM1, NCL, and RPL18 with FANCA was compared to that with FANCD2. Although NCL and NPM1 were also coIP with FANCD2, their association was markedly weaker than with FANCA, whereas FANCD2 failed to coIP RPL18 or FANCG (Figure 2I).

Collectively, the IP-MS and IP-immunoblotting data support a role for FANCA in multiple protein complexes mediated by interactions governed by distinct biophysical forces of varying strength, possibly contributing to or associating with dynamic supramolecular assemblies, including biological condensates.^23^

### FANCA interactors link FANCA to genetic information processing and metabolic processes

To unravel the biological functions associated with identified FANCA companions, the four *X*-based datasets were analyzed using three functional term libraries: the Kyoto Encyclopedia of Genes and Genomes (KEGG, version 113.0),^24^ the Gene Ontology biological process (GO_BP), and the Gene Ontology cellular components (GO_CC) (release 2025–03-16).^25^ The functional analysis was performed using the ClusterProfiler R package.^26^

**Figure 3:**
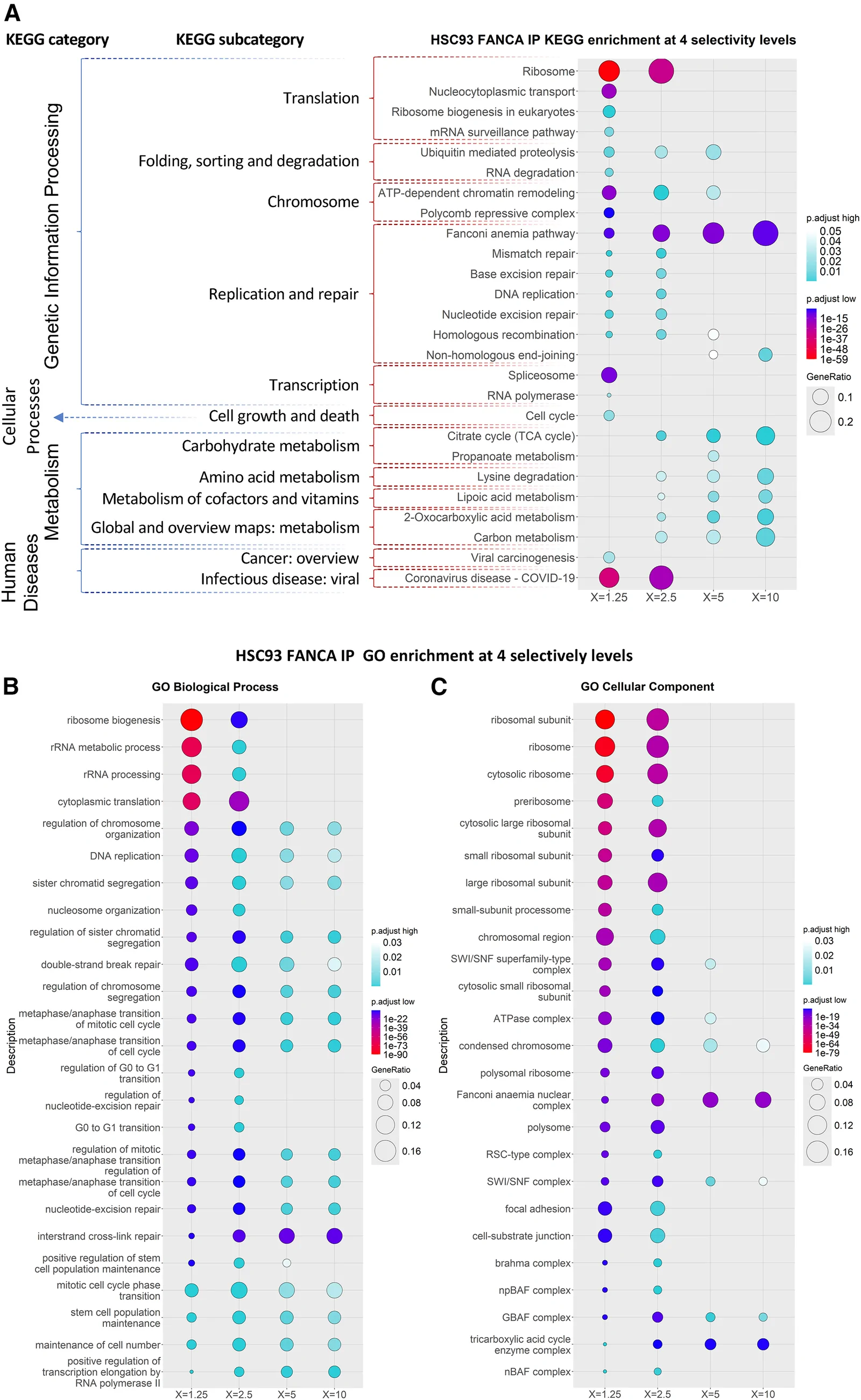
Functional enrichment analysis of FANCA coIP preys at different level of selectivity (A) Significantly enriched functional KEGG terms, as identified by ClusterProfileR-based analysis, at *X* = 1.25, *X* = 2.5, *X* = 5, and *X* = 10. (B–C) (B) GO_BP and (C) GO_CC most enriched terms in HSC93 FANCA IP as identified by ClusterProfileR-based analysis, at *X* = 1.25, *X* = 2.5, *X* = 5, and *X* = 10.

At *X* = 1.25, we identified 19 enriched KEGG pathways, belonging to four categories, namely “genetic information processing,” “human diseases,” “cellular processes,” and “metabolism”. Consistent with the role of FANCA in DNA repair and replication rescue, 6 of the 19 enriched terms belong to the subcategory “replication and repair,” namely “FA pathway,” “mismatch repair,” “base excision repair,” “DNA replication,” “nucleotide excision repair,” and “homologous recombination”. Among the other genetic information processing enriched terms, two are related to chromatin dynamics, namely “ATP-dependent chromatin remodeling” and “polycomb repressive complex”, four are included in the “translation” subcategory, i.e., “ribosome” (the most enriched KEGG term at *X* = 1.25, adjusted *p* value = 6.02 × 10^−60^), “ribosome biogenesis in eukaryotes,” “nucleocytoplasmic transport,” and “mRNA surveillance pathway”. Other mRNA-related terms are present: “spliceosome,” “RNA polymerase,” and “RNA degradation,” for which significant enrichment is lost at *X* = 2.5. Five new terms appear at this level, all related to carbon metabolism. At *X* = 5, all translation-related terms are lost. Six of the 11 retained terms are related to carbon metabolism. The others include FA pathway, ATP-dependent chromatin remodeling, homologous recombination, and “non-homologous end joining”. At the highest selectivity level studied, i.e., *X* = 10, 5 of the 7 terms are related to carbon metabolism, the remaining two being non-homologous end joining and FA pathway, which is the most significantly enriched term at *X* = 10 (*p* value = 09.3 × 10^−9^) (Figure 3A; Table S1, sheet 7). Functional enrichment using GO_BP and GO_CC terms was consistent with the KEGG enrichment, with GO_CC term “FA nuclear complex” and GO_BP term “interstrand crosslink repair” being significantly enriched at all selectivity levels, whereas the RNA processing- and translation-related processes/complexes are enriched at the lower *X* levels (Figures 3B and 3C).

These results suggest a selectivity-dependent functional stratification of FANCA’s interactome: at high selectivity (*X* ≥ 5), FANCA is primarily associated with proteins involved in genome stability maintenance (e.g., DNA repair and chromatin remodeling): at lower selectivity (*X* ≤ 2.5), FANCA interacts with proteins contributing to gene expression processes, spanning transcription, RNA processing, ribosome biogenesis, and translation. This functional dichotomy underscores FANCA’s multifaceted role in coordinating genetic information processing and metabolic regulation, with its interactions reflecting a hierarchy of biological priorities depending on the strength of association.

### Integrative analysis of FANCA interaction datasets

To further support and expand the FANCA interactome, we performed two complementary experiments. First, we repeated the FANCA IP-MS analysis using extracts from RPE1 human cells, an hTERT-immortalized, p53-proficient epithelial cell line. Second, we employed a proximity-dependent biotinylation strategy (bio-identification [BioID]) coupled to MS analysis in Flp-In HEK293 cells expressing a FANCA-BirA fusion protein. While IP-MS captures interactions based on biochemical affinity, BioID identifies proteins in spatial proximity to FANCA, thereby mapping its proximity interactome (proxilome).

RPE1-derived FANCA IP-MS identified 303 candidate interactors, selected based on detection by at least three unique peptides in FANCA IP samples and complete absence in IgG controls, as observed for the known FANCA interactor FAAP20 (Table S2, sheet 1; Table S3, sheet 1). BioID-MS analysis yielded a list of 344 biotinylated proteins (Table S4, sheet 1), from which two subsets were defined based on intensity ratios (IR) between FANCA-BirA and control samples: IR ≥ 1.5 (217 proteins) and IR ≥ 5 (133 proteins) (Table S2, sheet 1; Table S4, sheets 2–3).

**Figure 4:**
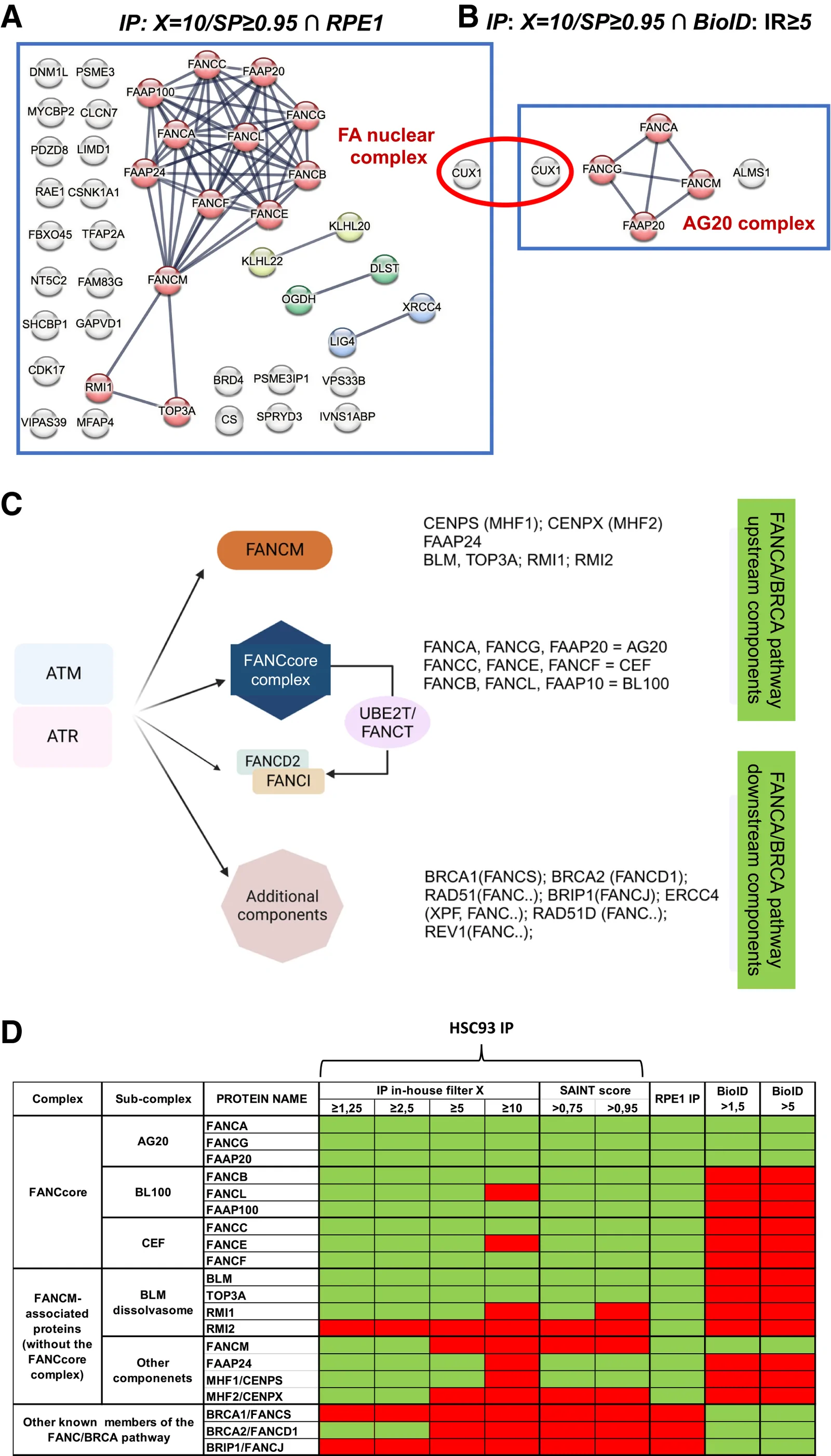
STRING based clustering of FANCA preys at different levels of selectivity as emerging from datasets obtained following IP or BioID analysis in different cells (A–B) (A) Proteins identified as FANCA partners in the *X* = 10 and SP > 0.95 lists and present in the RPE1 IP, or (B) in the BioID ≥ 5 X analysis. Clusters were proposed by STRING on the basis of the following setting: network type “physical subnetwork”; meaning of network edge “confidence”; minimum required interaction score “highest confidence” (0.9); for (C) and (D), “hide disconnected node in the network”; Markov Cluster Algorithm (MCL) clustering with an inflation parameter of 3 without edges between the clusters. (C) Schematic representation of the FANC/BRCA pathway. As indicated, the FANC core complex consists of the assembly of three subcomplexes, each of three FANC proteins, AG20, CEF and BL100. It acts as an E3 ubiquitin ligase that catalyzes, via FANCL, the transfer of a ubiquitin moiety from the E2 enzyme UBE2T/FANCT to its two substrates: FANCD2 and FANCI. The monoubiquitylation of both FANCD2 and FANCI is required for their assembly into chromatin-associated nuclear foci to guide the recruitment of the FANCA/BRCA pathway downstream components leading to homologous recombination. (D) Interactions of FANCA within FANCA/BRCA pathway proteins identified by mass spectrometry (MS) in co-immunoprecipitation (coIP) or bio-identification (BioID) approaches, revealed by different analytical methods. In green, the protein is identified; in red, it is absent in the approach and the corresponding data analysis.

Although these datasets are derived from single experiments and therefore lack statistical depth, their integration with the original IP-MS data contributes to a more comprehensive and robust view of the FANCA interaction landscape. Among the 93 proteins in the most selective HSC93-derived dataset (*X* = 0 and/or SP > 0.95), 43 (46%) were also detected in the RPE1 IP dataset, whereas only 6 (5.4%), i.e., FANCA itself, FANCG, FAAP20, FANCM, ALMS1, and CUX1, were identified in the BioID dataset at IR ≥ 5 (Figures 4A and 4B; Table S2, sheet 2). Notably, aside from FANCG and FAAP20, core components of the AG20 subcomplex (Figure 4C), only CUX1 was consistently identified as a FANCA interactor across all three experimental platforms (HSC93 IP, RPE1 IP, and HEK293 BioID) and at the highest selectivity thresholds (Figures 4A and 4B). At lower selectivity thresholds, dataset overlaps increased substantially (Table S2, sheet 2).

The distinct characteristics of IP and BioID were evident in their differential ability to capture known FANCA-associated complexes (Figures 4C, 4D and S1B). BioID robustly detected FANCG and FAAP20, as well as FANCM, BRCA1 (FANCS), its interacting partner BACH1/BRIP1 (FANCJ), and BRCA2 (FANCD1), but missed most other components of the FANCcore complex or FANCM-associated partners. In contrast, combined IP analyses in HSC93 and RPE1 cells identified all components of the FANCcore and FANCM complexes, but not FANCS or FANCJ.

**Figure 5:**
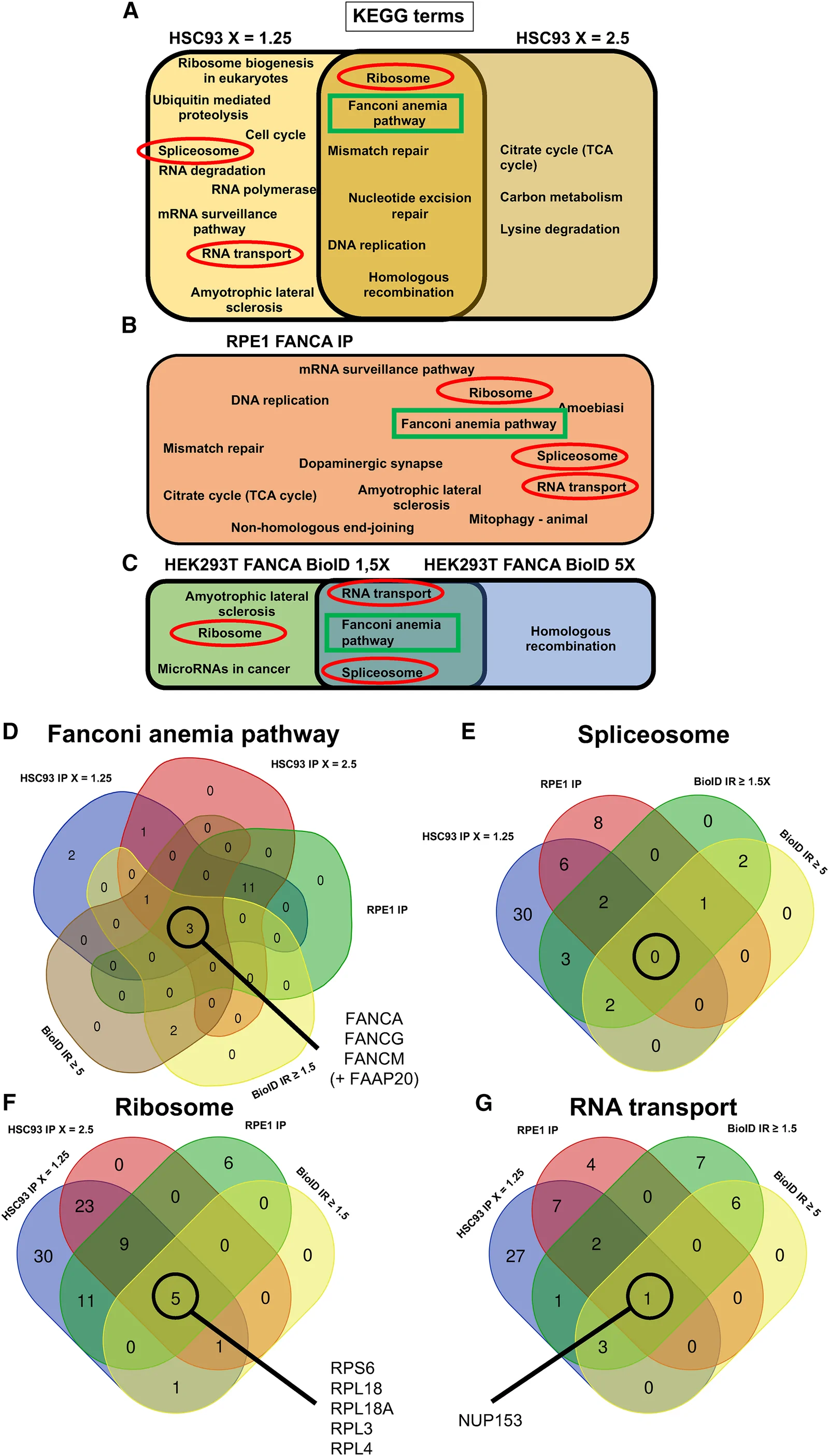
Functional enrichment analysis of FANCA preys from different datasets (A) Venn diagram representing the overlap of enriched KEGG terms between the *X* = 1.25 and *X* = 2.5 datasets from the coIP in HSC93 cells. (B) KEGG enriched terms emerging from the FANCA partners identified in the RPE1 cells. (C) Venn diagram representing the overlap of enriched KEGG terms identified in the BioID ≥ 1.5 and BioID ≥ 5 lists issues from the Flp-in HEK293 cell expressing the FANCA fused with BirA. (D–G) Venn diagram representing the overlap of proteins contributing to the enrichment of KEGG term Fanconi anemia (D), spliceosome (E), ribosome (F), and RNA transport (G) in the datasets we generated.

To further validate our findings, we used the STRING database^27^ to perform KEGG pathway enrichment analyses on the HSC93 IP datasets, as well as on the RPE1 IP and HEK293 BioID datasets. For the most selective HSC93 datasets (*X* = 5, *X* = 10, SP > 0.75, and SP > 0.95), the number of enriched KEGG terms identified by STRING was too limited to allow meaningful comparisons. In contrast, KEGG terms retrieved from the *X* = 1.25 and *X* = 2.5 datasets largely recapitulated those previously identified using ClusterProfiler (Figure 3A vs; Figure 5A), with minor differences likely reflecting distinct multiple-testing correction strategies, as both tools rely on hypergeometric testing. STRING-based KEGG enrichment analysis of the RPE1 IP dataset identified pathways related to translation (with the ribosome term being the most significantly enriched), RNA metabolism (spliceosome, mRNA surveillance pathway, and “RNA transport”), the FA pathway, mitochondrial processes (“Tricarboxylic Acid (TCA) cycle” and “mitophagy,” a process reported to be altered in FA,^24^ as well as several pathways associated with DNA transactions (Figure 5B). KEGG enrichment analysis of the BioID dataset identified FA pathway, spliceosome, homologous recombination, and RNA transport (Figure 5C). Only one KEGG term, FA pathway, was enriched across all five datasets, while four additional terms, homologous recombination, ribosome, RNA transport, and spliceosome, were enriched in four out of five datasets (Figures 5A–5C). Despite this convergence at the pathway level, the proteins contributing to these enriched terms were largely distinct across datasets (Figures 5D–5G). For example, enrichment of the FA pathway term was driven by 18, 16, 14, and 6 proteins in the *X* = 1.25, *X* = 2.5, RPE1 IP, and BioID IR ≥ 5 datasets, respectively. However, only three proteins, FANCA, FANCG, and FANCM, were common to all datasets. We additionally included FAAP20, the third component of the FANCA-FANCG-FAAP20 subcomplex, which is not annotated in KEGG as part of the FA pathway (Figure 5). Similarly, enrichment of the ribosome term was driven by 80, 38, 31, and 20 proteins in the HSC93 IP *X* = 1.25, RPE1 IP, BioID IR ≥ 1.5, and BioID IR ≥ 5 datasets, respectively. However, only five proteins (RPS6, RPL18, RPL18A, RPL3, and RPL4) were shared across these datasets, with all but one (RPS6) belonging to the 60S large ribosomal subunit. For the RNA transport term, only a single protein, NUP153, a component of the nuclear pore complex (NPC), was common across datasets, while no proteins were shared across all datasets for the spliceosome term (Figures 5E–5G).

Finally, we compiled a reference list of known FANCA interactors by aggregating several sources: the 159 proteins listed in the BioGRID database (version 4.4.240),^28^ the 124 proteins identified by coIP and SAINT analysis (SP > 0.85, a value we chose as intermediate between our SP > 0.75 and SP > 0.95 datasets) in HEK293 human embryonic kidney cells reported by Lagundžin et al.^29^ and some additional interactors described in the literature but not included in the BioGRID dataset.^20^^,^^30^^,^^31^ This integration resulted in a reference dataset of 306 FANCA-associated proteins (Table S2, sheet 3). Twenty-three of the 93 proteins (24.7%) identified in our high-confidence HSC93-derived FANCA interactome had been previously reported as FANCA partners (Table S2, sheet 4). When restricting the comparison to the 159 interactors curated in BioGRID, we identified 16 proteins (10.5%) among the 153 FANCA-associated proteins detected at selectivity level X = 5 and 13 proteins (14%) among our most stringent high-confidence set (X = 10 and/or SP > 0.95). These proportions exceed those reported by Lagundžin et al.,^29^ who identified only 7 previously reported FANCA interactors (5.6%) among 124 co-immunoprecipitated proteins (SP > 0.85), a dataset comparable in size to our X = 5 selectivity level (Table S2, sheet 3). KEGG analysis of the Lagundžin et al.^29^ and the BioGRID datasets as well as of the 306 list of unique proteins identified at least one time as FANCA interactors converge essentially on FA pathway and other process involved in DNA transaction (repair and replication) (Table S2, sheet 3).

Among the known FANCA interactors and beyond the accepted constituents of FANC pathway, we retrieved several previous identified FANCA partners, including components related to chromatin-associated processes, as BRD4,^29^ the SWI/SNF chromatin remodeling complex, SMARCA4 (alias BRG1),^30^^,^^32^ PBRM1 (BAF180),^33^ and SMARCD2,^34^ and of the RFC and RFC-like complexes, i.e., RFC2-5, RAD17 and CHTF18,^29^^,^^34^ the NPC proteins NUP50 ^29^ and NUP155 ^30^, the nuclear-cytoplasmic shuttles KPNA1, KPNA4 and KPNB1,^29^^,^^30^ three components of the R2TP complex, i.e., RUVBL1,^35^ RPAP3^29^ and PELP1,^29^ the chaperone HSP90B1 ^36^, the nucleolus key components NCL and NPM1, the ribosomal protein RPL18 ^30^, the translation-related protein LARP1,^29^ the glycolytic enzymes ALDOA and ENO1^30^ (Table S2, sheet 3). Together, these comparisons underscore the robustness and sensitivity of our data acquisition and filtering strategy.

Together, these analyses highlight two key points. First, the limited overlap of individual proteins contributing to shared functional enrichments underscores the necessity of integrating multiple methodologies to capture the full spectrum of a protein’s interactome. Each approach reveals distinct yet complementary interaction landscapes, reflecting context-dependent and biophysically diverse associations. Second, although protein overlaps were limited, the datasets converged at the functional level, reinforcing the idea that FANCA acts as a hub linking DNA repair, RNA processing, nucleocytoplasmic transport, translation, and metabolism through diverse interactions.

In conclusion, our *in silico* analyses support the involvement of FANCA in multiple functional networks: a canonical network centered on DNA repair and chromatin/chromosome dynamics; and emerging networks involved in RNA processing, nucleocytoplasmic trafficking, translation, and cellular metabolism. Together, these analyses reveal a consistent functional stratification of the FANCA interactome, with genome maintenance dominating high-selectivity interactions and ribosome biogenesis and translation emerging as the most recurrent non-canonical functions at lower selectivity levels.

### A multilayered *in silico* FANCA PPI landscape

In order to assemble a comprehensive FANCA PPI network, we searched for proteins common to the HSC93 IP-MS datasets (*X* = 1.25 and *X* = 10/SP > 0.95), the BioID datasets (IR ≥ 1.5 and IR ≥ 5), the RPE1 IP dataset, and the literature-derived reference list, which make a total of 6 datasets. Each protein was assigned an interaction score ranging from 1 to 6, indicating the number of independent datasets in which it was detected. The strongest layer of FANCA interactors is made of those found in all 6 datasets: FANCG, FAAP20, and FANCM. Conversely, the weakest interaction layer is made of the proteins found in only one dataset.

**Figure 6:**
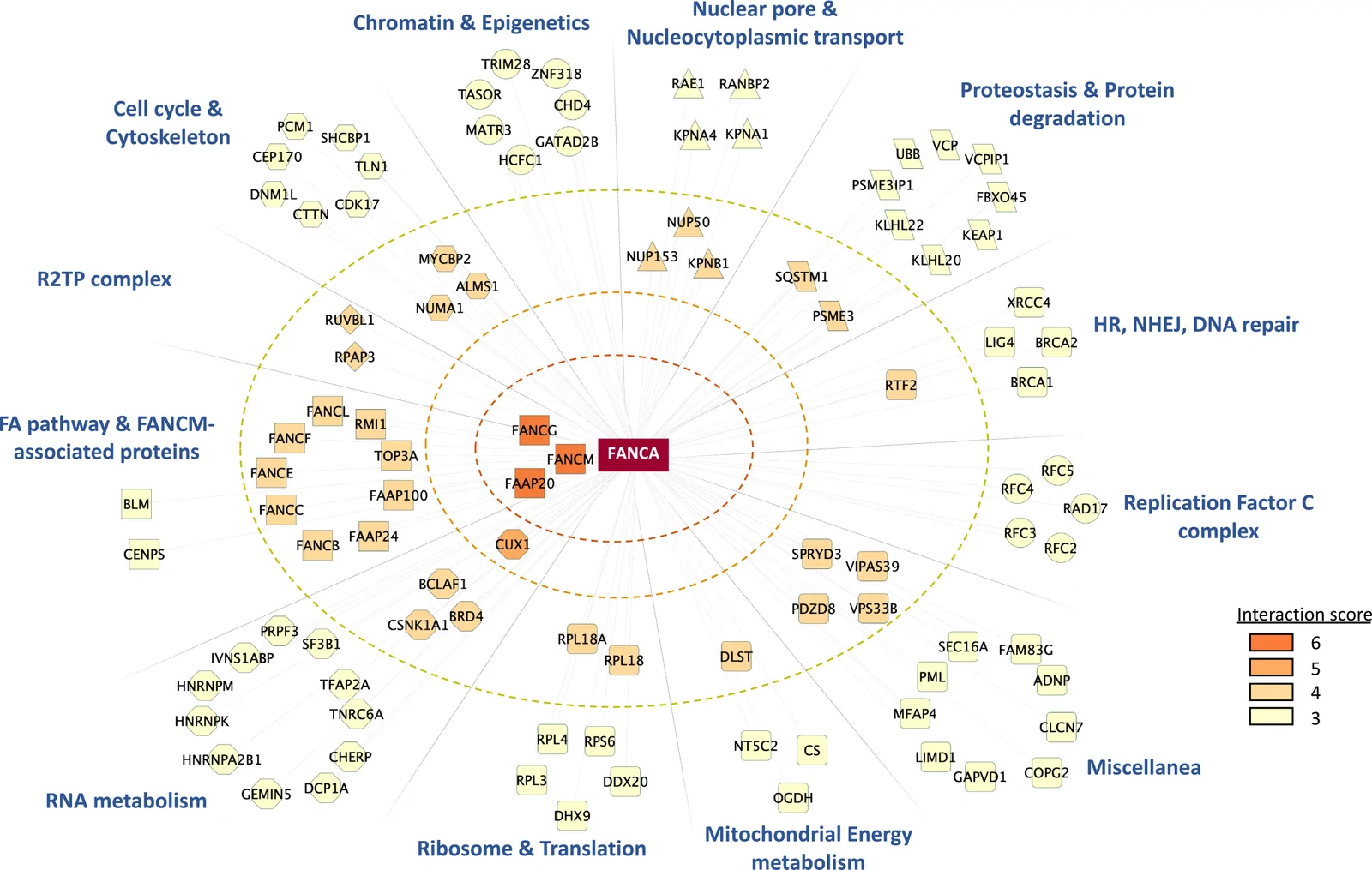
FANCA PPI landscape (A) Schematic representation of the major sub-networks that characterize the FANCA interactions landscape.

For better representation, we show a network composed only of proteins with a score ≥ 3 (Figure 6 and 100 proteins). Node color indicates interaction score. The proteins of the high selectivity list were assembled in 11 functional groups and one “miscellanea” group based on their KEGG and GO annotations. Another, more complete, network of proteins with a score ≥ 2 was also constructed (Figure 2 and 371 proteins). As expected, the central layer comprises proteins participating in the FA pathway core complex. Outer layers include a multiplicity of pathways such as translation, mRNA maturation and nucleocytoplasmic transport (Figures 6 and S2).

By assigning an interaction score based on recurrence across independent experimental methods and literature sources, we established a hierarchical view of the FANCA interaction landscape. This scoring strategy enables discrimination between core FANCA partners, and peripheral or context-dependent associations. This integrative framework provides a comprehensive view of FANCA’s involvement in multiple cellular networks. Indeed, FANCA does not operate within a single, static interaction landscape. Instead, it participates in multiple, partially overlapping interaction networks that are differentially captured depending on the experimental approach, cellular context, and selectivity criterion.

### The FANCA PPI network strengthens its biological connection to ribosome biogenesis

Although a comprehensive dissection of FANCA’s role within each identified protein network is beyond the scope of this study, our data provide strong evidence that FANCA is functionally connected to ribosome biogenesis and translational control. The enrichment of ribosomal proteins within the FANCA PPI network prompted us to directly test whether FANCA loss alters ribosome composition and function. We therefore performed polysome profiling and analyzed fractionated proteins in two independent experiments.

**Figure 7:**
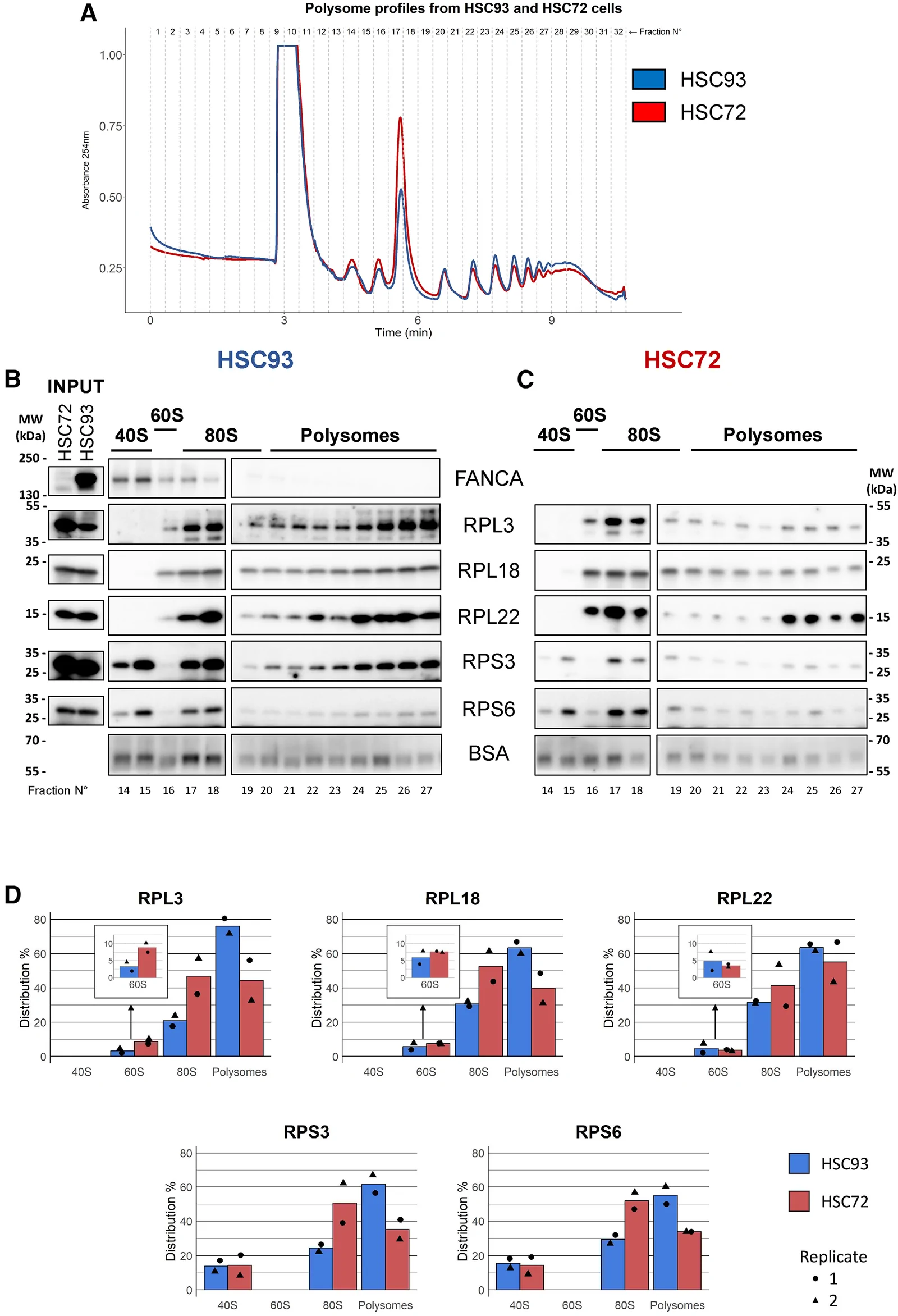
FANCA contributes to ribosome protein stoichiometry and polysome organization (A) A254nM polysome profile in exponentially growing HSC93 (FANCA-proficient, blue) and HSC72 (FANCA-deficient, red) cells. (B and C) Western blot showing the distribution of indicated RPS and RPL proteins in the 40S, 60S, 80S, and polysomal fractions. BSA (200 ng) added in each fraction before TCA extraction from sucrose fraction was used as standard for quantification (spike-in), in HSC93 and HSC72 cells. Input is showed on the left for both HSC72 and HSC93 cell lines. Please refer to Data S1 for the original uncropped blots showing the MW scale and expected MW of the analyzed proteins, as well as images taken at different exposure times. (D) The distribution of the protein/BSA signal was calculated in each fraction for proteins RPL3, RPL18, RPL22, RPS3, and RPS6. The distribution percentages in fractions were summed by RNP species (40S, 60S, 80S, and polysomes). Dots represent two independent experiments and bar height represents mean.

Polysome profiling revealed a pronounced accumulation of ribosomal proteins in the 80S fractions and a concomitant reduction in polysomes in HSC72 (FANCA-deficient) cells compared with HSC93 (FANCA-proficient) cells (Figure 7A). This shift is indicative of impaired productive translation, characterized by the accumulation of translationally inactive 80S ribosomes not bound to mRNA, and/or poised single ribosomes.^36^^,^^37^^,^^38^ The overrepresentation of single 80S versus polysomes is consistent with our previous finding that FANCA loss reduces global translation rates.^20^ To determine whether this defect reflects altered ribosome composition, we quantitatively analyzed the distribution of some ribosomal proteins previously identified as putative FANCA interactors, i.e., RPL3, RPL18, RPL22, RPS3, and RPS6, across the 40S, 60S, 80S, and polysome fractions (Figures 7B, 7C and S3; Table S5). Our analysis identified changes in the relative abundance of specific ribosomal proteins in the ribosomal subunits between FANCA-proficient and FANCA-deficient cells. Consistent with previous MS analyses,^20^ these results suggest that FANCA loss leads to heterogeneous changes in ribosomal subunit composition rather than a single uniform alteration. Indeed, the distribution of SSU proteins (RPS3 and RPS6) within the 40S fraction was largely unaffected by FANCA status. In contrast, LSU proteins exhibited distinct behaviors within the 60S fraction: RPL3 showed a disproportionate enrichment in free 60S subunits upon FANCA loss, whereas RPL18 and RPL22 did not, indicating selective perturbation of LSU composition rather than increased LSU abundance. With the exception of RPL22, all analyzed proteins were over-represented in the 80S fraction. Within polysomes, RPS3, RPS6, RPL3, and RPL18 displayed reduced association in FANCA-deficient cells, while RPL22 remained unchanged. These non-uniform effects across ribosomal fractions indicate the formation of compositionally heterogeneous ribosomes, a hallmark of defective ribosome biogenesis or maturation (Figure 7D).

Consistent with this model, FANCA itself is detected in the 40S, 60S, and 80S fractions (Figure 7B), positioning it at multiple stages of ribosome assembly and function. Notably, FANCA seems enriched in the 40S fraction (Figure 7B), consistently with our previous observations.^20^ Reconstitution of the 3D ribosome structure,^39^^,^^40^ highlighting the localization and co-localization of RPS6 and RPL3 on the ribosomal surface (Figures S4A and S4B), further supports the possibility that a FANCA-RPS6 interaction contributes to FANCA association with the 40S subunit and potentially to bridging between the two ribosomal subunits. Through direct or indirect interactions primarily with the SSU and, to a lesser extent, with LSU components such as RPL18, FANCA may facilitate proper or efficient 80S assembly and/or contribute in ribosome quality control. In the absence of FANCA, aberrant LSUs with altered protein stoichiometry may still associate with SSUs to form 80S complexes that are inefficient at initiating or sustaining translation, resulting in the accumulation of inactive monosomes and reduced polysome formation.

This model provides a mechanistic explanation for the elevated monosome-to-polysome ratio observed in A254 nm profiles and positions FANCA as a regulator of ribosome integrity and translational capacity. Notably, in our previous work, we identified a distinct set of ribosomal proteins, including the 40S protein RPS27A and the 60S proteins RPL22L1, RPL39, and RPL7L1, as recurrently differentially distributed in the 80S fractions across four independent experiments, whereas no major differences were detected in the composition of ribosomes present in the polysome fractions.^20^ The simplest interpretation of these findings is that FANCA contributes to efficient ribosomal subunit assembly and/or inter-subunit bridging, not through specificity for individual ribosomal proteins, but rather by promoting the formation of correctly assembled ribosomal complexes. Together, these findings extend FANCA function beyond DNA repair and support a role for FANCA in coordinating genome maintenance with protein synthesis capacity.

## Discussion

**Figure 8:**
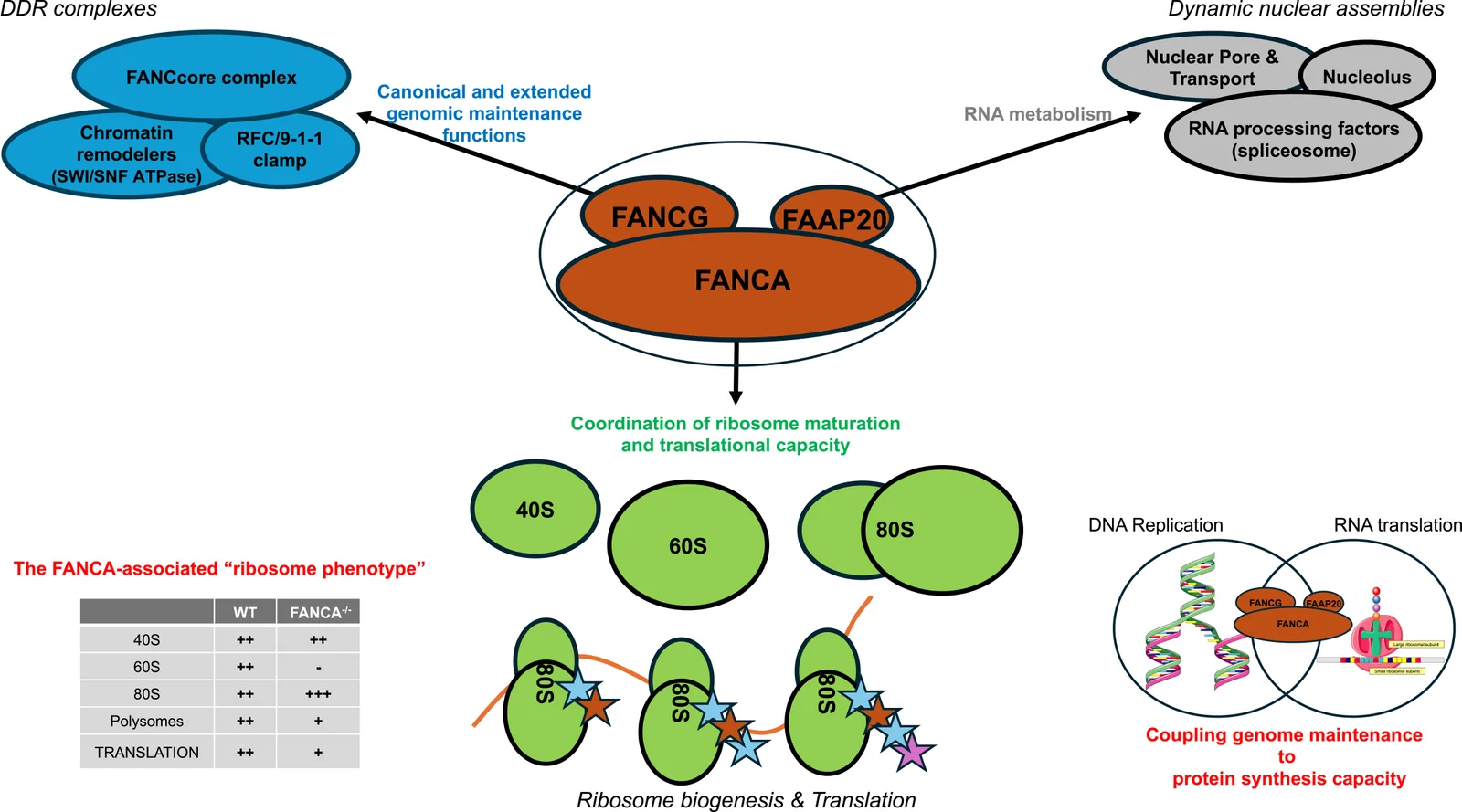
FANCA’s interactions place it at the center of cellular physiology FANCA’s mutifunctional role in cellular physiology. FANCA, in complex with its obligate partnersFANCG and FAAP20, occupies a central position at the interface between genome maintenance, nuclear organization, and protein synthesis. Beyond its canonical association with the FANCcore complex and DNA repair machineries, FANCA interfaces with chromatin remodelers, nucleocytoplasmic transport factors, and RNA-processing assemblies. Integrative interactomics and functional analyses identify ribosome biogenesis and translation as central non-canonical functions associated with FANCA. FANCA associates with free ribosomal subunits and 80S complexes and contributes to proper large ribosomal subunit maturation and translational competence, likely through transient interactions. In FANCA-deficient cells, altered ribosomal protein stoichiometry leads to accumulation of inactive 80S monosomes and reduced polysome formation. Together, these data support a model in which FANCA acts as a multifunctional adapter coordinating genome stability with translational capacity rather than as a pathway-specific enzymatic regulator.

FA has traditionally been viewed as a genome instability disorder caused by defects in the FANC/BRCA DNA repair pathway. While this framework explains hypersensitivity to DNA interstrand crosslinks and replication stress and cancer predisposition, it does not fully account for the clinical heterogeneity of FA. Through an integrative interactomics approach, we redefine FANCA as a multifunctional hub coordinating genome maintenance with gene expression programs, extending its functional scope to ribosome biogenesis and translational control (Figure 8).

By combining endogenous coIP, proximity-dependent labeling, *in silico* integration, and functional assays, we generated a multilayered FANCA interaction landscape. This strategy confirms FANCA’s canonical association with DNA repair machineries while revealing a broader, hierarchically organized network linking FANCA to chromatin remodeling, RNA metabolism, i.e., transcription, splicing, nucleocytoplasmic transport, and protein synthesis. Importantly, convergence across experimental platforms occurs primarily at the functional level, highlighting FANCA as a coordinator of genetic information flow rather than a static component of a single pathway.

Indeed, our findings raise the possibility that FANCA may function as a factor with dual DNA- and RNA-binding or -interaction functions. While FANCA has been extensively studied in the context of DNA repair, its strong affinity for single stranded (ss) RNA^41^ suggests that RNA binding may represent a conserved and functionally significant property of the protein. FANCA could engage several RNA species, contributing to their transcription, processing, transport, and/or association with other protein complexes, including ribosomes, functioning as an RNA chaperone or scaffold that stabilizes transient folding states or coordinates ribonucleoprotein complex formation. Thus, FANCA and possibly other FANC pathway components may operate at the intersection of DNA integrity, RNA metabolism, and proteostasis, forming part of a previously unappreciated regulatory layer. Moreover, FANCA is known to be able to shuttle between the cytoplasm, nucleus, and nucleolus,^20^^,^^42^^,^^43^^,^^44^ supporting a dynamic role as a coordinator of genome integrity, gene expression, and protein synthesis capacity. FANCA may act as a molecular rheostat that helps balance DNA repair, transcription, and translation in response to cellular needs. For instance, under conditions of genotoxic stress, FANCA could be preferentially retained in the nucleus to support DNA repair, concomitantly dampening transcription, ribosome biogenesis, and global translation as part of a broader stress adaptation program. Conversely, in proliferative states, increased cytoplasmic localization of FANCA might enhance translational output. This raises the possibility that FANCA participates in signaling networks that couple genome maintenance to biosynthetic capacity, ensuring that protein synthesis is aligned with DNA damage response (DDR) and DNA integrity.

At high interaction selectivity, FANCA associates predominantly with core components of the FA pathway, homologous recombination factors, and chromatin remodeling complexes, consistent with its established role in replication stress responses. We further identify connections to RFC and RFC-like complexes and to non-homologous end joining factors, suggesting that FANCA influences the coordination and balance of DNA repair pathways. Its associations with NPC components and transport factors extend its regulatory scope to nuclear organization and factor accessibility.

A major and unexpected outcome of our integrative analysis is the strong and recurrent association of FANCA with proteins involved in ribosome biogenesis and translation-related processes. Across independent datasets, ribosomal proteins and ribosome-associated pathways emerged among the most significantly enriched functional categories, particularly at lower selectivity thresholds that capture biologically coherent yet weaker/more indirect interactions. Importantly, this enrichment does not reflect a generic property of large interactomes, but rather represents a robust and reproducible signature of FANCA-associated networks across cell types and experimental approaches.

Beyond FANCA, previous studies have already suggested direct or functional links between FANC proteins and factors involved in nucleolar homeostasis, ribosome biogenesis, and ribosomal structure. For instance, a two-hybrid screen identified RPL18 and RPS3A as interactors of FANCC, which was also reported to associate with eIF2AK2 (Pang, 2002; Zhang, 2004). Pladevall-Morera et al. identified, among FANCD2 interactors, nucleolar proteins such as NCL and NPM1, translation initiation factors including eIF4A1, eIF4A3, and eIF6, as well as a large proportion of 40S and 60S ribosomal subunit components,^45^ including RPS27L, whose loss leads to a reduced expression of both FANCD2 and FANCI and to an FA-like cellular phenotype,^46^ FANCI has been shown to interact with rRNA, and its loss results in defects in ribosome biogenesis and reduced global translation. An outcome we also reported in the absence of FANCA. FANCI has also been identified as a partner of translation initiation factors EIF2S2 and EIF2S3,^47^ as well as several ribosomal proteins, including RPL31, RPS24, RPS26, and RPS6.^48^^,^^49^^,^^50^ Finally, supporting a more direct role of FANCA, FANCI, and FANCD2 in linking DDR processes to RNA metabolism, ribosome biogenesis, and/or translation, we previously reported that MS analysis of proteins present in 40S, 60S, and 80S fractions obtained from polysome profiling identified only FANCA, FANCD2, and FANCI among the components of the FANC pathway.^20^ It is important to keep in mind that the loss of function of any component of the FANCcore complex can influence, to varying degrees, the expression levels or subcellular localization of the other components. In other words, even though no direct or functional connection has been established between, for instance, FANCL, FANCB, or FANCE and RNA metabolism or ribosome biogenesis, their loss may still alter RNA and/or ribosome behavior by impacting FANCA expression and localization. Regardless of the mechanisms, functionally, FANCA deficiency appears alter the translational organization. Polysome profiling reveals an accumulation of 80S monosomes accompanied by reduced polysome formation, indicative of impaired translation. Importantly, this phenotype is not associated with a uniform loss of ribosomal subunits, but rather with alterations in the assembling of the ribosomal proteins into the subunits. Such non-uniform changes are hallmarks of defective ribosome biogenesis or maturation, leading to the formation of compositionally heterogeneous ribosomes with reduced translational competence.

The presence of FANCA within 40S, 60S, and 80S fractions positions it at multiple stages of ribosome assembly and function. Our data analysis indicates a strong association of FANCA with RPS6, RPL18, RPL18A, RPL3, and RPL4. However, consistently with present (Figure 7B) and previous observations, the data further suggest a more pronounced association of FANCA with the 40S and 80S complexes than with the isolated 60S subunit. One possible explanation of such conundrum is that FANCA associates more efficiently with RPS6 (a component of the 40S subunit) than with RPL3 (located on the 60S subunit in proximity to RPS6) or with the other nearby ribosomal proteins, contributing to bridging the two ribosomal subunits (Figures S4A and S4B). Alternatively, though not mutually exclusively, FANCA may have an intrinsically higher affinity for the 18S rRNA in the region between RPS6 and RPL3, appearing as more associated with the 40S and 80S than the 60S. FANCA may contribute, through direct or indirect interactions with subunits components, to ribosome biogenesis or maintenance, ensuring proper ribosomal protein incorporation or surveillance prior to translation start. In the absence of FANCA, aberrant ribosomal subunits may still assemble into 80S complexes but fail to efficiently initiate or sustain translation, providing a mechanistic explanation for the elevated monosome-to-polysome ratio observed in FANCA-deficient cells.

These findings extend previous reports implicating FANCA and other FA proteins in nucleolar homeostasis and ribosome biogenesis,^4^^,^^20^^,^^21^ and establish translational control as a novel function of FANCA. Given the tight coupling between ribosome production, cell growth, and differentiation, disruption of this axis offers a compelling explanation for the pleiotropic manifestations of FA, particularly in highly proliferative and differentiation-sensitive tissues such as the hematopoietic system.

Beyond discrete molecular complexes, many FANCA-associated proteins are key constituents of dynamic nuclear assemblies, including the nucleolus, nuclear speckles, and transport-associated compartments. FANCA interacts with nucleolar proteins such as NPM1 and NCL, spliceosome components, and nucleocytoplasmic transport factors, all of which are enriched in biomolecular condensates that spatially organize RNA processing and ribosome production.

While our data do not demonstrate a role for FANCA in condensate formation, they support the idea that FANCA interfaces with condensate-associated machineries. FANCA contains short intrinsically disordered regions, and its obligate partner FAAP20 is largely disordered^51^^,^^52^^,^^53^ (Figures S5A–S5C), suggesting that the FANCA-FAAP20 module may be well suited to participate in dynamic, multivalent interaction environments. In this context, FANCA may act as a scaffold or adapter that promotes functional coupling between DNA repair, RNA metabolism, and ribosome biogenesis within spatially organized nuclear domains. This conceptual framework provides a unifying explanation for how FANCA can influence diverse cellular processes without possessing any enzymatic activity. By facilitating interactions within dynamic assemblies, FANCA could coordinate cellular responses to replicative stress, ensuring that genome maintenance is aligned with transcriptional and translational capacity. This model is proposed as a conceptual framework rather than a demonstrated mechanism.

Our findings support a model in which FA is not solely a consequence of defective DNA repair, but also reflects impaired coordination between genome maintenance and protein synthesis. Disruption of ribosome biogenesis and translation is expected to disproportionately affect stem and progenitor cells, offering a mechanistic link to bone marrow failure and developmental abnormalities. Moreover, altered translational output may contribute to metabolic rewiring and stress signaling, exacerbating inflammation and cancer susceptibility in FA patients.

In conclusion, our findings reposition FANCA from a pathway-specific DNA repair factor to a multifunctional coordinator of genome stability and expression, from transcription to translation, providing a conceptual framework for the pleiotropic nature of FA and highlighting translational control as a potential therapeutic vulnerability.

### Limitations of the study

Several limitations should be acknowledged. Our interactomics approach does not distinguish direct physical interactions from indirect or proximity-based associations, and additional biochemical and structural studies will be required to define the precise molecular interfaces involved. Furthermore, our analyses were performed in a limited number of cellular contexts, and FANCA interaction landscapes are likely to be modulated according to cell types and stress conditions.

Future work should aim to dissect the molecular mechanisms by which FANCA influences ribosome assembly, quality control, and translational initiation, as well as to determine how these functions intersect with its canonical role in genome maintenance. Extending these analyses to primary hematopoietic cells and *in vivo* models will be essential to assess the contribution of FANCA’s non-canonical functions to disease pathogenesis and therapeutic response.

In summary, beyond redefining FANCA as a multifunctional hub that links genome stability to ribosome biogenesis and translational control, our study illustrates more broadly the power of integrative interaction analyses to uncover non-canonical functions of well-studied proteins and provides a conceptual framework for understanding the complexity of FA.

## Resource availability

### Lead contact

Requests for further information and resources should be directed to and will be fulfilled by the lead contact, Filippo Rosselli (filippo.rosselli@gustaveroussy.fr).

### Materials availability

This study did not generate new unique reagents.

### Data and code availability

1. All data are available in the main text or in the supplementary materials. The mass spectrometry proteomics data of the coIP realized in the HSC93 cell line have been deposited to the ProteomeXchange Consortium via the PRIDE partner repository with the dataset identifier PXD068730 (https://www.ebi.ac.uk/pride/archive/projects/PXD068730).
2. This paper does not report original code.
3. Any additional information required to reanalyze the data reported in this paper is available from the lead contact upon request.

## Acknowledgments

The Rosselli lab was supported was supported by grants from La Ligue Contre Le Cancer, the French National Cancer Institute INCa (PLBIO 2021), by the 10.13039/501100001665Agence Nationale de la Recherche (ANR, FANC-Diff), SIRIC EpiCure (INCa-DGOS-Inserm-ITMO Cancer_18002) and from Enfants Cancer Santé (ECS)/Societe Française de lutte contre les Cancers et les leucémies de l'Enfant et de l’adolescent (SFCE). The Constantinou lab was supported by the French National Cancer Institute INCa (PLBIO 2021), by the French Agence Nationale de la Recherche ANR (AAPG2021 and AAPG2023), and by the Fondation MSD AVENIR. This work was supported by the DIM Thérapie Génique Paris Ile-de-France Région, 10.13039/100015510IBiSA, and the Labex GR-Ex. BioID-associated mass spectrometry experiments were carried out using the facilities of the Montpellier Proteomics Platform (PPM, BioCampus Montpellier), a member of the national Proteomics French Infrastructure (ProFI UAR 2048) supported by the 10.13039/501100001665French National Research Agency (ANR-24-INBS-0015, Investments for the future F2030).

## Author contributions

Conceptualization, V.G., F.R., F.M.-C., A.C., and J. Basbous; investigation, V.G., F.M.-C., B.M., E.-F.G., S.U., A.H.-L., J. Basbous; data analysis, V.G., F.R., J. Bruce, M.L.G., S.U., and J. Basbous; writing, V.G. and F.R.; review & editing, all authors contributed.

## Declaration of interests

All other authors declare they have no competing interests.

## Declaration of generative AI and AI-assisted technologies in the writing process

During the preparation of this work the authors used ChatGPT & Chat.MISTRAL. AI in order to check grammar and spelling, and rephrasing the original manuscript. After using this tool/service, the authors reviewed and edited the content as needed and take full responsibility for the content of the published article. Authors do not use AI tools to generate figures, images, and artworks.

## STAR★Methods

### Key resources table

**Table:** 

REAGENT or RESOURCE	SOURCE	IDENTIFIER
**Antibodies**		

Rabbit anti-FANCA	Bethyl	Ref #: A301-980; RRID: AB_1547945
Mouse anti-FANCG	Santa Cruz Biotechnology	Ref #: sc-393382; RRID:
Mouse anti-FANCD2	Santa-Cruz Biotechnology	Ref #: sc-20022; RRID: AB_2278211
Rabbit-*anti*-FANCD2	Abcam	Ref #: AB108928; RRID: AB_10862535
Mouse anti-a-tubulin	Sigma	Ref #: T5168; RRID: AB_10862535
Mouse anti-Nucleolin (NCL)	Abcam	Ref #: AB13541; RRID: AB_300442
Mouse anti Nucleophosmin (NPM1)	Thermo Fischer Scientific	Ref #: 32–5200; RRID: AB_86912
Rabbit anti Ligase4 (LIG4)	Proteintech	Ref #: 12695-1-AP; RRID: AB_2136253
Rabbit anti-XRCC4	Proteintech	Ref #: 15817-1-AP; RRID: AB_2257478
Mouse anti-CUX1	Proteintech	Ref #: 68449-1-Ig; RRID:
Rabbit anti-RAE1	Proteintech	Ref #: 20491-1-AP; RRID: AB_2878693
Rabbit anti-INVS1ABP	Proteintech	Ref #: 14741-1-AP; RRID: AB_10638784
Mouse anti-NF45	Santa-Cruz Biotechnology	Ref #: Sc-365283 RRID: AB_10851344
Mouse anti-NF90	Santa-Cruz Biotechnology	Ref #: Sc-377406 RRID: AB_3083533
Rabbit anti-RPL3	Abcam	Ref #: AB154882 RRID:
Rabbit anti-RPL18	Novus Biological	Ref #: NBP2-13251 RRID: AB_3260959
Mouse anti-RPL22	Santa Cruz Biotechnology	Ref #: sc-136413; RRID: AB_10658965
Rabbit anti-RPS3	Millipore/Merck	Ref #: ABE391
Mouse anti-RPS6	Cell Signaling Technology	Ref #: 2317; RRID: AB_2238583
Rabbit anti-BSA	Thermo Fischer Scientific	Ref #: A11133; RRID: AB_2534139
Donkey anti rabbit IgG(H + L) HRP conjugated	Ozyme	Ref #: BETA120-108P
Goat anti-Mouse IgG(H&L) cross-adsorbed Ab HRP Conjugated	Ozyme	Ref #: BETA90-516P
Negative Control Rabbit Immunoglobulin Fraction	Agilent	Ref #: X090302; RRID: AB_906174

**Chemicals, peptides, and recombinant proteins**		

Benzonase Nuclease	Sigma-Aldrich	Ref #: 70746–3
cOmplete™ Mini EDTA-free Protease Inhibitor tablets	Sigma-Aldrich	Ref #: 5892791001
PhosSTOP	Sigma-Aldrich	Ref #: 4906837001
Bio-Rad Protein Assay Dye Reagent Concentrate	Bio-Rad	Ref #: 5000006
Dynabeads protein G for immunoprecipitation	Thermo Fisher Scientific	Ref #: 10004D
RiboLock RNAse Inhibitor	Thermo Fisher Scientific	Ref #: E00382
RNasin	Promega	Ref #: N2511
D-Sucrose	Fisher Scientific	Ref #: 10638403
Cycloheximide	Sigma-Aldrich	Ref #: C7698
Trichloroacetic acid (TCA)	Sigma-Aldrich	Ref #: 36612
Mitomycin C	Sigma-Aldrich	Ref #: M0503
Zeocin	Thermo Fisher Scientific	Ref #: R25001
Blasticidin	InvivoGen	Ref #: ant-bl
Hygromycin B	Sigma-Aldrich	Ref #: H3274
Aphidicolin	Sigma-Aldrich	Ref #: A0781
Doxycycline	Clontech	Ref #: 631311
RPMI	Gibco/Thermo Fisher scientific	Ref #: 61870–010
DMEM	Gibco/Thermo Fisher scientific	Ref #: 41965–039
DMEM-F12	Gibco/Thermo Fisher scientific	Ref #: 11320–074
Pénicilline-streptomycine (10 000 U/ml)	Gibco	Ref #:15140–122
2X Laemmli Sample Buffer	Bio-Rad	Ref #: 1610737
Sequencing Grade Modified Trypsin	Promega	Ref #: V5111
Opti-MEM	Life Technologies	Ref #: 15140–122
cOmplete, EDTA free	Roche	Ref #: 4693159001
BSA	Thermo Fisher scientific	Ref #:23209
Sodium Pyruvate (100 mM)	Gibco	Ref #:11360–039

**Critical commercial assays**		

WesternBright Quantum Kit	Diagomics	Ref #: K-12042-D20
WesternBright ECL HRP Substrate Kit	Diagomics	Ref #: K-12045-D50
Super Signal West Femto maximum Sensitivity	Thermo Fisher Scientific	Ref #: 34095
Substrat Super Signal West Dura extend 100 mL	Thermo Fisher Scientific	Ref #: 34075
Rabbit TrueBlot® ULTRA	Rockland	Ref #: 18–8816-31; RRID: AB_2610847
Bradford assay Kit I	Bio-Rad	5000001

**Deposited data**		

MS proteomics data of the co-IP realized in the HSC93 cell line	This paper	ProteomeXchange Consortium via the PRIDE repository. Identifier: PXD068730

**Experimental models: Cell lines**		

HSC93 (GM13072)	Coriell Cell Repository	RRID:CVCL_G049
HSC72 (GM13022)	Coriell Cell Repository	RRID:CVCL_AK37
HeLa	ATCC	RRID: CVCL_0030
U-2 0S	ATCC	RRID:CVCL_0042
hTERT-RPE-1	ATCC	RRID:CVCL_4388
MRC5	N/A	N/A
HEK293	N/A	N/A
Flp-In 293 T-REx	Invitrogen	Ref #:R78007; RRID:CVCL_U427

**Recombinant DNA**		

pOG44 Flp-Recombinase Expression Vector	ThermoFisher Scientific	Cat# V600520
pDONR-FANCA	This paper	
pDEST-pcDNA5-BirA-FLAG N-term-FANCA	This paper	
pCDNA5_FRT-TO_Flag-BirA∗	N/A	

**Software and algorithms**		

REPRINT APMS	https://reprint-apms.org	
STRING	https://string-db.org	
BioGRID	https://thebiogrid.org	
VENN	https://bioinformatics.psb.ugent.be/webtools/Venn/	
AIUPred	https://aiupred.elte.hu	
Depicter2	http://biomine.cs.vcu.edu/servers/DEPICTER2/	
Finches	https://www.finches-online.com	
KEGG	https://www.genome.jp/kegg/	
GO	https://geneontology.org/	
ClusterProfiler R		
Cytoscape	https://cytoscape.org/	
UCSF ChimeraX version 1.12	https://www.cgl.ucsf.edu/chimerax	
Biorender	https://www.biorender.com/	
Servier Medical Art (SMART)	https://smart.servier.com/	
Xcalibur software v4.0	Thermo Fisher Scientific	Version 4.0
MaxQuant software v1.6.5.0	Max Planck Institute/MaxQuant	Version 1.6.5.0
ImageJ	NIH/Fiji ImageJ	https://imagej.nih.gov/ij/
DIA-NN v1.8.1	DIA-NN	Version 1.8.1
PROXIMA R Shiny Script	Marjorie Leduc	https://github.com/MarjorieLeduc/PROXIMA

### Experimental model and study participant details

#### Cell lines, culture conditions and treatments

EBV-immortalized lymphoblastoid cell lines HSC93 (FANCA wild type) and HSC72 (FANCA deficient), were grown in RPMI supplemented with 13% fetal bovine serum, 100 U/ml penicillin and 100 μg/mL streptomycin (all from Invitrogen). HeLa, HeLa-Kyoto, U2OS, HEK293, and MRC5 were grown in Dulbecco’s modified Eagle’s medium (DMEM) (Life Technologies) supplemented with 10% fetal calf serum (FCS), 100 U/ml penicillin, 100 μg/mL streptomycin and 1 mM pyruvate. RPE1-hTERT cells were grown in DMEM-F12 supplemented with 10% FBS, 100 U/ml penicillin and 100 μg/mL streptomycin.

Flp-In 293 T-REx and the Flp-In 293 T-REx derived stable cell lines were grown under standard cell culture conditions in Dulbecco’s modified Eagle’s medium (DMEM, Merck-Sigma-Aldrich, D5796) containing 10% fetal bovine serum (BioWest S1810-500) and penicillin-streptomycin. Parental cells were selected with 100 μg/mL Zeocin (ThermoFisher Scientific R25001) and 15 μg/mL Blasticidin (InvivoGen, ant-bl) and Flp-In 293 T-REx derived stable cell lines were maintained with 5 μg/mL Blasticidin (InvivoGen, ant-bl) and 50 μg/mL Hygromycin B (Sigma-Aldrich, H3274).

All cells were cultivated at 37°C under a 5% CO2 atmosphere and were routinely tested for mycoplasma and scored negative.

Mitomycin C (Sigma-Aldrich, M0503) and aphidicolin (Sigma-Aldrich, A0781) were prepared in H_2_O and added for 18 to 24 h.

No animal or human participants or primary human samples were used in this study. The study used established human cell lines. Cell line provenance and RRIDs are provided in the Key Resources Table. Cell line authentication was not otherwise reported in the source manuscript. All cells were routinely tested for mycoplasma contamination and scored negative.

### Method details

#### Protein extraction and immunoprecipitation

Cellular extracts for immunoprecipitation were prepared from exponentially growing cells lysed on ice for 30 min in NETN buffer (150 mM or 300 mM NaCl, 1 mM EDTA, 50 mM Tris (pH 8.0), 0.5% NP-40), supplemented with phosphatase inhibitor PhosSTOP (Roche), cOmplete ULTRA EDTA-Free Protease inhibitors (Roche), benzonase (Merck, 1 mM) and 1 mM MgCl_2_. Extracts were sonicated for 2 × 10 s at 30% (Vibracell 75042, Bioblock) and spun down for 5 min at 13 000 rpm. Supernatants were quantified using the Bradford assay (BioPhotometer, Eppendorf). One milligram of protein extract was used per immunoprecipitation with rabbit anti-FANCA (Bethyl, A301-980) or isotypic anti-IgG (Dako).

For each immunoprecipitation, antibodies (3 μg) were coupled to 20 μL of magnetic beads (Dynabeads Protein G Magnetic Beads, Thermo Fisher Scientific). Beads washed in 1× PBS (two times) were resuspended in 300 μL of 1× PBS and incubated with antibodies for at least 2 h in a roll shaker in a cold room. To preclear the protein extracts, 10 μL of beads washed in PBS and in NETN buffer was added to each whole protein extract in the presence of 0.5 μg of control immunoglobulin G (IgG) and incubated for at least 2 h in a roll shaker in a cold room. Beads coupled with IgG were collected on a magnetic support and discarded. A small amount of the precleared protein extract was kept as input. Precleared supernatants were incubated with the magnetic beads coupled to the antibodies overnight in a roll shaker in a cold room. Beads coupled with antibodies were captured on a magnetic support and washed four times with 500 μL of NETN buffer before being resuspended in 20–40 μL of 2× Laemmli buffer and heated for 5 min at 70°C to dissociate proteins and antibodies from the beads. The supernatants were transferred into a new tube, 0.5 to 1 μL of b-mercaptoethanol was added. For WB analysis, immunoprecipitated proteins are loaded on an acrylamide gel after 5 min of heating at 98°C.

#### Proteomics analysis on IP samples

##### Sample preparation

The Co-IP samples were solubilized in lysis buffer (2% SDS, 200 mM Tris-HCl, pH 8.0, 10 mM TCEP, 50 mM chloroacetamide). Bottom-up experiments’ tryptic peptides were obtained by Strap Micro Spin Column according to the manufacturer’s protocol (Protifi, NY, USA). Briefly: Proteins were digested during 14 h at 37°C with 1 μg Trypsin sequencing grade (Promega). The Strap Micro Spin Column was used according to the manufacturer’s protocol. After speed-vacuum drying, eluted peptides were solubilized in 2% trifluoroacetic acid (TFA).

#### Liquid chromatography-coupled mass spectrometry analysis (nLC-MS/MS)

nLC-MS/MS analyses were performed on a Dionex U3000 HPLC nanoflow chromatographic system (Thermo Fischer Scientific) coupled to a TIMS-TOF Pro mass spectrometer (Bruker Daltonics). Peptides were solubilized in 10 μL of 0.1% trifluoroacetic acid (TFA) in 10% Acetonitrile (ACN). One μL was loaded, concentrated and washed for 3 min on a C18 reverse phase Pepmap *neo* (3 μm particle size, 300 μm inner diameter, 5 mm length, from Thermo Fisher Scientific). Peptides were then separated at 50°C on an Aurora C18 reverse phase resin (1.6 μm particle size, 100 Å pore size, 75 μm inner diameter, 25 cm length) (IonOpticks) with a 60 min overall run-time gradient from 99% of solvent A containing 0.1% formic acid in milliQ-grade H2O to 40% of solvent B containing 80% acetonitrile, 0.085% formic acid in mQH2O with a flow rate of 400 nL/min. The mass spectrometer acquired data throughout the elution process in a positive mode and operated in DIA PASEF mode with a 1.38 s/cycle, with Timed Ion Mobility Spectrometry (TIMS) enabled. Capillary voltage was set to 1,500 V. Ion accumulation and ramp time in the dual TIMS analyzer were set to 100 ms each. The MS1 spectra were collected in the m/z range of 100–1 700. The DIA PASEF window scheme ranged in dimensions from m/z 400 to 1 200 and in dimension 1/K0 from 0.63 to 1.43. The collision energy was set by linear interpolation between 59 eV at an inverse reduced mobility (1/K0) of 1.60 versus/cm^2^ and 20 eV at 0.6 versus/cm^2^.

#### Protein identifications and quantifications

The mass spectrometry data were analyzed using DIA-NN version 1.8.1.^54^ The database used for *in silico* generation of spectral library was a concatenation of Human sequences from the Swissprot database (release 2022-05) and a list of contaminant sequences from Maxquant and from the cRAP (common Repository of Adventitious Proteins). M-Terminus exclusion and carbamidomethylation of cysteines was set as permanent modification and one trypsin mis-cleavage was allowed. Precursor false discovery rate (FDR) was kept below 1%. The “match between runs” (MBR) and the normalization option was allowed.

The results files of DIA-NN were analyzed using PROXIMA R Shiny Script (https://github.com/MarjorieLeduc/PROXIMA). *t* test greater Unpaired were done on proteins showing at least 3 valid values in one group and at least 70% of valid values in the other group using log2(LFQ intensity). Significant threshold is PValue<0.05.

#### Protein identification by BioID assay

A doxycycline-inducible cDNA encoding FANCA fused to the mutant biotin ligase BirA∗ and an FLAG epitope was stably integrated into Flp-In HEK293 cells. The R118G mutation in BirA (BirA∗) enables the transfer of biotin to nearby proteins, which are subsequently enriched using streptavidin-coated beads and identified by MS.

#### Plasmid construct

To generate pDEST-pcDNA5-BirA-FLAG N-term-FANCA, the full-length FANCA cDNA from the pDONR-FANCA construct was inserted into the pDEST-pcDNA5-BirA-FLAG N-term vector (Gift from Anne-Claude Gingras) using a Gateway recombination assay.

#### Generation of stable cell lines

Flp-In 293 T-REx cells are seeded to reach 80–90% confluence on the day of transfection. pDest-pcDNA5-BirA-Flag-FANCA expression plasmid was mixed with pOG44 encoding the Flp recombinase (Thermo-Fisher Scientific, V600520) at a 1:7 ratio in opti-MEM (Gibco, 31985-047). For a single transfection in a 6 well plate, 500 ng of the expression plasmid was mixed with 3.5 mg of pOG44 in 250 μL opti-MEM. Additionally, 8 μL Lipofectamine 2000 Transfection Reagent (ThermoFisher Scientific, 11668-019) was added to 250 μL opti-MEM. After an incubation period of 5 min at room temperature, both solutions were mixed and incubated for a further 15 min at room temperature. The mixture was then pipetted dropwise onto the cells. The medium was changed after 6 h. At 48 h post-transfection, the cells were transferred to a 100 mm Petri dish, and 24 h later, the selection was performed by adding 5 μg/mL Blasticidin and 50 μg/mL Hygromycin B. Clones were pooled, and the cells were examined for the expression of the construct by immunoblotting.

#### Affinity capture of biotinylated proteins: BioID

Flp-In™ 293 T-Rex cell lines stably transfected with BirA∗-Flag-FANCA grown to 75% confluence were incubated with 1 μg/mL of doxycycline (Clontech, 631311) for 16 h and with 50 mM biotin for 16 h. Cells were washed with PBS and lysed with lysis buffer (50 mM Tris-HCl pH 7.5, 150 mM NaCl, 1 mM EDTA, 1 mM EGTA, 1% NP-40, 0.2% SDS, 0.5% Sodium deoxycholate) supplemented with 1X complete protease inhibitor (Roche, 4693159001) and 250U benzonase (Sigma, CE1014). Lysed cells were incubated on a rotating wheel for 1 h at 4°C prior sonication on ice (40% amplitude, 3 cycles 10 s sonication with 2 s resting). After 30min centrifugation (7750 rcf) at 4°C, the cleared supernatant was transferred to a new tube and total protein concentration was determined by Bradford protein assay (BioRad, C500-0205). For each condition, 300 mg of proteins were incubated with 30 mL of Streptavidin-Agarose beads (Sigma, CS1638) on a rotating wheel at 4°C for 3 h. After 1min centrifugation (400 rcf), beads were washed, successively, with 1 mL of lysis buffer, 1 mL wash buffer 1 (2% SDS in H_2_O), 1 mL wash buffer 2 (0.2% sodium deoxycholate, 1% Triton X-100, 500 mM NaCl, 1 mM EDTA, and 50 mM HEPES pH 7.5), 1 mL wash buffer 3 (250 mM LiCl, 0.5% NP-40, 0.5% sodium deoxycholate, 1 mM EDTA, 500 mM NaCl and 10 mM Tris pH 8) and 1 mL wash buffer 4 (50 mM Tris pH 7.5 and 50 mM NaCl). Bound proteins were eluted from the agarose beads using 40 μL of 2X Laemmli Sample buffer and sent for mass spectrometry analysis.

#### Mass spectrometry on BioID samples

Sample digestion was essentially performed as described.^55^ Briefly, proteins were loaded on an SDS-PAGE (BioRad, 456–1034) and, after short migration, a single band was excised. Proteins in the excised band were digested with Trypsin (Promega). The resulting peptides were analyzed online by nano-flow HPLC-nanoelectrospray ionization using a Qexactive HFX mass spectrometer (Thermo Fisher Scientific) coupled to a nano-LC system (Thermo Fisher Scientific, U3000-RSLC). Desalting and preconcentration of samples were performed online on a Pepmap precolumn (0.3 3 10 mm; Fisher Scientific, 164568). A gradient consisting of 0%–40% B in A (A: 0.1% formic acid (Fisher Scientific, A117), 6% acetonitrile (Fisher Scientific, A955), in H_2_O (Fisher Scientific, W6), and B: 0.1% formic acid in 80% acetonitrile) for 120 min at 300 nL/min was used to elute peptides from the capillary reverse-phase column (0.075 3 250 mm, Pepmap, Fisher Scientific, 164941). Data were acquired using the Xcalibur software (version 4.0). A cycle of one full-scan mass spectrum (375–1 500 m/z) at a resolution of 60 000 (at 200 m/z) followed by 12 data-dependent MS/MS spectra (at a resolution of 30 000, isolation window 1.2 m/z) was repeated continuously throughout the nanoLC separation. Raw data analysis was performed using the MaxQuant software (version 1.6.5.0) with standard settings. Used database consist of Human entries from Uniprot (reference proteome UniProt 2019_09) and 250 contaminants (MaxQuant contaminant database).

#### Protein extraction, cellular fractionation, and western blot analysis

For proteins expression, cells collected by centrifugation or on Petri dishes were disrupted in lysis buffer [50 mM Tris-HCl pH 7.5, 20 mM NaCl, 1 mM MgCl2, 0.1% SDS and benzonase (Merck), supplemented with protease and phosphatase inhibitors (Roche)]. After 20 min of incubation at room temperature, the protein concentration was determined using the Bradford assay, and samples were combined with 4× Laemmli buffer containing β-mercaptoethanol and denatured by boiling.

For Western blot, proteins from immunoprecipitation, cellular extracts and cell fractions were separated by SDS‒PAGE by electrophoretic migration performed in 25 mM Tris, 192 mM glycine, and 0.1% SDS buffer. Semidry transfer was performed with a TransBlot cell apparatus (Bio-Rad) in transfer buffer composed of 25 mM Tris, 0.192 M glycine, and 20% isopropanol for 1 h and 30 min at 20 V. Nitrocellulose membranes (Protran 0.2 mm, Amersham) were blocked for at least 1 h in 0.1% PBS–Tween 20 and 5% milk and incubated with primary antibodies in PBS, 0.05–0.1% Tween 20 and 5% milk. Visualization was performed using ECL (Life), Western Bright ECL or Quantum (Advansta), or West Dura and Femto ECL (Thermo Fisher Scientific) developer. Images were acquired using Amersham Imager 600 or 680 (GE Healthcare). All western blot quantifications were performed using densitometry measures and ImageJ software.

#### Polysome profiling, fraction collection and protein extraction

Cycloheximide (100 μg/mL) was added for 5 min to cell cultures and maintained in PBS washes. Cells were fractionated in 5 mM tris (pH 7.5), 2.5 mM MgCl2, and 1.5 mM KCl buffer supplemented with cOmplete ULTRA EDTA-free protease inhibitors (Roche), cycloheximide (100 μg/mL), RNase inhibitor (RNasin (Promega) 0.2 U/μl or Ribolock RNAse Inhibitor, Thermo Fischer), 2 mM DTT, 0.5% Triton, and 0.5% sodium deoxycholate, and nuclei were discarded after a 7 min 15 000 g centrifugation. The cytoplasmic optical density (OD) at 260 nm was assessed, 300 μg of RNA were resuspended in a final volume of 500 μL, 50ul was kept as input and 450ul loaded on a 5 to 50% sucrose gradient [20 mM HEPES (pH 7.6), 100 mM KCl, 5 mM MgCl2, 10 μg/mL cycloheximide, 1/10 protease inhibitors, and RNase inhibitor (10 U/ml)] and ultracentrifuged for 2 h at 36 000 rpm at 4°C in a Beckman SW41Ti rotor. Absorbance at 254 nm of the content of the ultracentrifuge tube was measured from top to bottom using a UA-6 UV/VIS detector and 500 μL fractions were collected automatically by the machine.

Proteins were extracted by the classical TCA (Trichloro acetic acid/Acetone) method. 200 ng of BSA was added to each fraction before the extraction as an internal control. 10% TCA was added to each fraction; after 15 min of incubation on ice; the samples were centrifugated 5 min at 15 000 rpm at 4°C. Pellets were precipitated/washed 2 times with cold acetone and rapidly dried at RT before resuspension in 40 μL Laemmli 1x containing b-mercaptoethanol (prewarmed at 55°C). Sample were denaturated 5min at 98°C before loading on acrylamide gel.

#### Web platforms and software

**REPRINT APMS** https://reprint-apms.org/was used to calculate SAINT score^56^
**STRING** https://string-db.org/cgi/^27^
**BioGRID** https://thebiogrid.org, Version 4.4.244^28^
**VENN** diagrams were realized on the with the platform Bioinformatics & Evolutionary Genomics https://bioinformatics.psb.ugent.be/webtools/Venn/
**AIUPred, DEPICTER2 and FINCHES** were used to identify Intrinsically Disordered Protein Regions^51^^,^^52^^,^^53^
**KEGG** https://www.genome.jp/kegg/^24^
**GO** https://geneontology.org/^25^
**ClusterProfiler R** package^26^
**Cytoscape** Version 3.10.4^57^
**UCSF ChimeraX version 1.12** https://www.cgl.ucsf.edu/chimerax/^40^
For the Graphical abstract and Figure 8, artworks were from BioRender (https://www.biorender.com/) and Servier Medical Art (SMART) (https://smart.servier.com/)
**Xcalibur** software v4.0 Thermo Fisher Scientific
**MaxQuant** software v1.6.5.0
**ImageJ** https://imagej.nih.gov/ij/
**DIA-NN v1.8.1** https://github.com/MarjorieLeduc/PROXIMA

### Quantification and statistical analysis

Quantitative analysis of Western blots was performed using the ImageJ Gel Analyzer tool. Figure 7D presents data from *n* = 2 independent experiments. Individual experiment values are shown as dots and the bar height represents the mean. Thus, the center measure is the mean and individual observations provide the dispersion of the data; no SD or SEM is reported for Figure 7D.

For differential protein abundance in the DIA-NN dataset, unpaired t-tests were performed in PROXIMA on proteins showing at least 3 valid values in one group and at least 70% valid values in the other group, using log2 LFQ intensity. The significance threshold was *p* < 0.05. The exact number of valid observations varied by protein according to these criteria; the underlying measurements are provided in the supplemental datasets.

Proteomics data processing used DIA-NN version 1.8.1 and PROXIMA R Shiny Script; BioID mass spectrometry data acquisition used Xcalibur software version 4.0 and raw-data analysis used MaxQuant version 1.6.5.0. Western blot densitometry used ImageJ. Other analyses used the software and web resources listed in the key resources table and method details.

All statistical details available in the manuscript are described here and in the relevant figure legends. No asterisk-based statistical annotations are reported in the figure legends of the submitted manuscript.

## Supplemental information

Document S1. Figures S1–S5 and Data S1

Table S1. Mass spectrometry data and FANCA co-immunoprecipitated prey lists from HSC93 cells, including selectivity filtering, SAINT scoring, and functional enrichment analyses Sheet 1: Identified proteins; Sheet 2–5: Proteins identified at selectivity levels from X = 1.25 to X = 10; Sheet 6: Proteins identified at SAINT score > 0.95; Sheet 7: KEGG based analysis.

Table S2. Integrated datasets of FANCA partners from HSC93 IP, RPE1 IP, BioID, and literature-derived interaction analyses Sheet 1: Experimentally identified datasets; Sheet 2: Common proteins between the HSC93, RPE1 and Bio_ID datasets; Sheet 3: Datasets from published data and corresponding KEGG analysis; Sheet 4: Common proteins between the literature and our IP and Bio_ID datasets; Sheet 5: Proposed FANCA PPI network.

Table S3. Mass spectrometry data and functional enrichment analyses of FANCA immunoprecipitation in RPE1 cells Sheet 1: RPE1 dataset; Sheet 2–4: KEGG, GO_BP, and GO_CC functional terms enrichment analysis.

Table S4. Mass spectrometry data and functional enrichment analyses of FANCA-BirA-mediated biotinylated targets at IR ≥ 1.5 and IR ≥ 5 Sheet 1: Bio_ID datasets; Sheet 2 and 3 4: KEGG, GO_BP, and GO_CC functional terms enrichment analysis for Bio_ID > 1.5 and > 5, respectively.

Table S5. Polysome fraction protein measurements and derived data used for the ribosome composition analyses Sheet 1: Polysome profiling 1, data; Sheet 2: Polysome profiling 2, data.
