# Supplementary material for: Integrative proteomics defines FANCA as a multifunctional hub linking DNA repair to translation
Source: iScience. 2026 Sep 9;29(9):117461. doi: 10.1016/j.isci.2026.117461 (PMC13581985; doi:10.1016/j.isci.2026.117461)
Supplement: Supplementary file 1 [file mmc1.pdf]

iScience, Volume *29*

Supplemental information

Integrative proteomics defines FANCA as a multifunctional hub linking DNA repair to translation

Virgile Gibert, Frédérique Maczkowiak-Chartois, Benedetta Mancini, Emilie-Fleur Gautier, Johanna Bruce, Morgane Le Gall, Serge Urbach, Angelos Constantinou, Anne Helbling-Leclerc, Jihane Basbous, and Filippo Rosselli

Supplemental Figure Legends

Figure S1: FANCA-associated proteins identified by SAINT analysis and selective interaction filtering.

(A) FANCA protein-protein network as determined by SAINT analysis of proteins co-immunoprecipitated with FANCA in HSC93 cells (SAINT score >0.95). (B) Progressive loss of FANCA and FANCM preys in the upper part of the FANC/BRCA pathway as a function of the selectivity levels.

Figure S2: Major FANCA interaction sub-networks identified at lower interaction-score thresholds.

Schematic representation of the major sub-networks that characterize the FANCA interaction landscape at lower interaction-score threshold.

Figure S3: Distribution of ribosomal protein signals across polysome fractions in FANCA-proficient and FANCA-deficient cells.

The distribution of the protein/BSA signal was calculated in each fraction for proteins RPL3, RPL18, RPL22, RPS3 and RPS6. Dots represent distribution percentages in each fraction in two independent experiments and bar height represents mean.

Figure S4: Ribosome models showing the positions of FANCA-associated ribosomal proteins.

(A and B) Schematic representation of the ribosome with (A) or without (B) rRNA showing the relative position of the ribosomal proteins that emerged as the most robust interactors of FANCA.

Figure S5: Predicted intrinsically disordered and structured regions of FANCA, FAAP20, and FANCG.

(A-C) Intrinsically Disordered Regions (IDRs) and Structured Regions in FANCA, FAAP20 and FANCG as predicted by (A) Metapredict (v3.0), (B) AIUPRED and (C) DEPICTER2 web sites.

Data S1: Original uncropped immunoblots for main Figures 2 and 7B–C.

Original blots for main Figure 2 and Figure 7B and 7C, including molecular-weight scales and different exposure times.

A

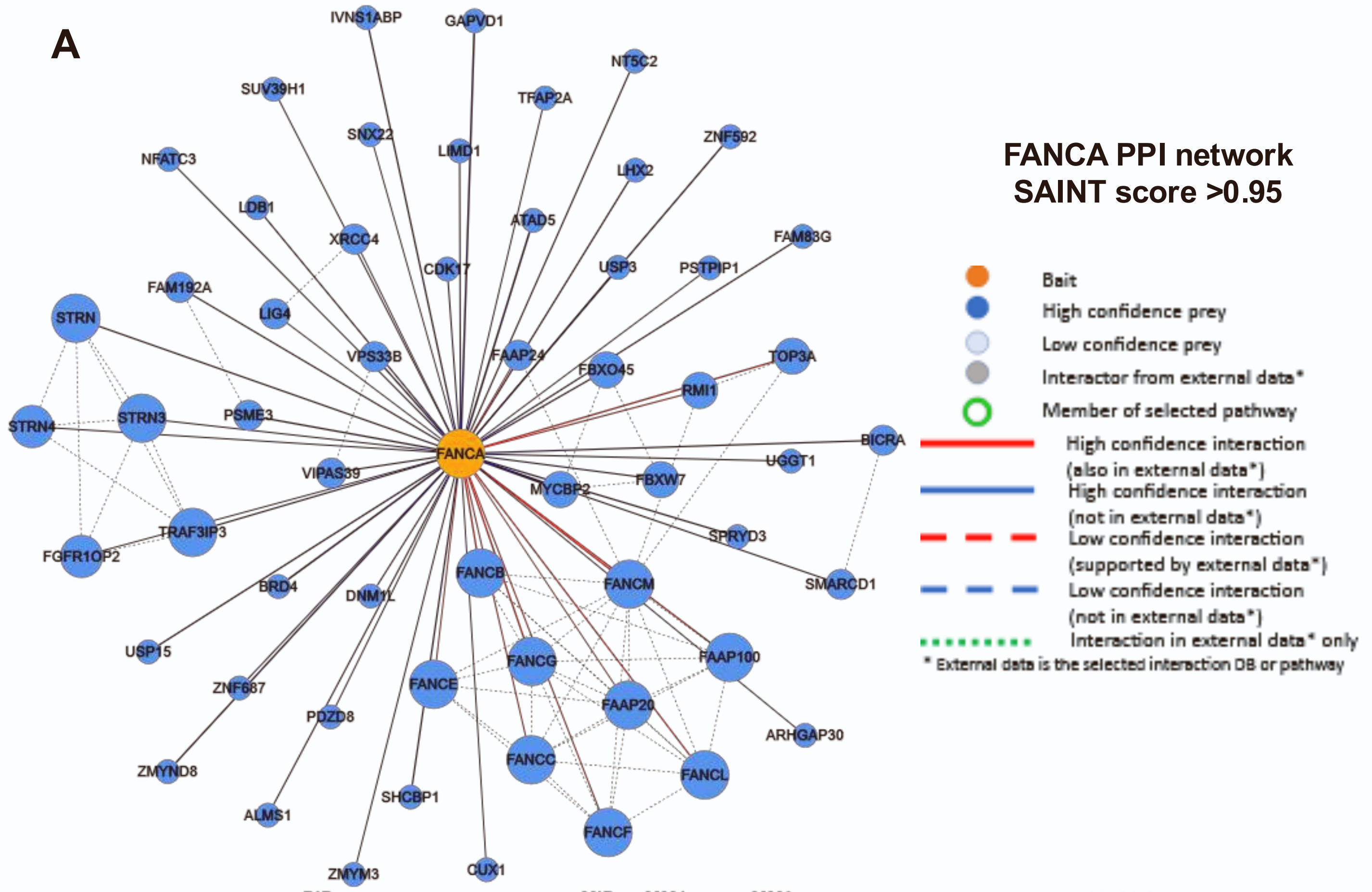

B

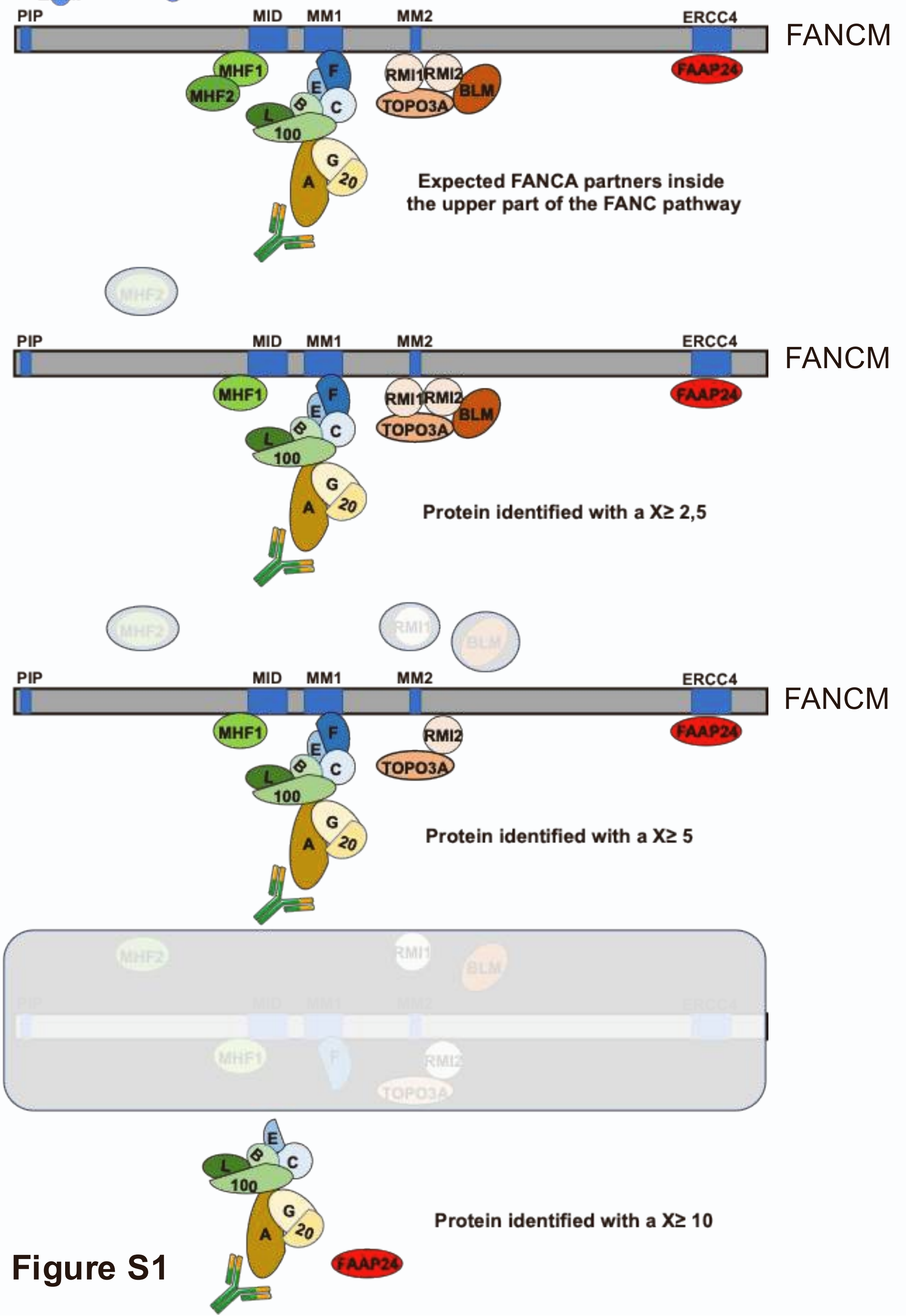

Supplemental Figure S1

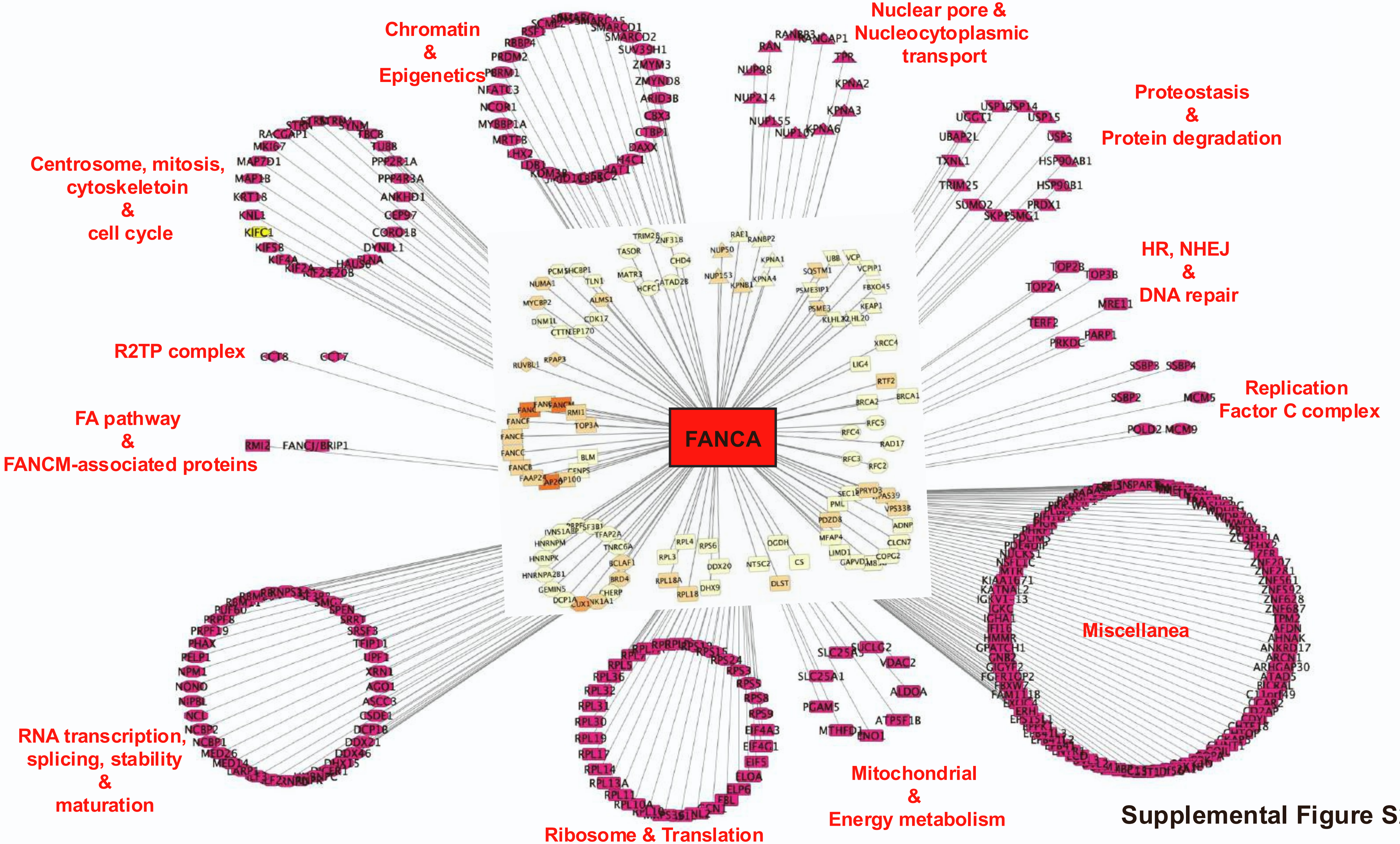

Supplemental Figure S2

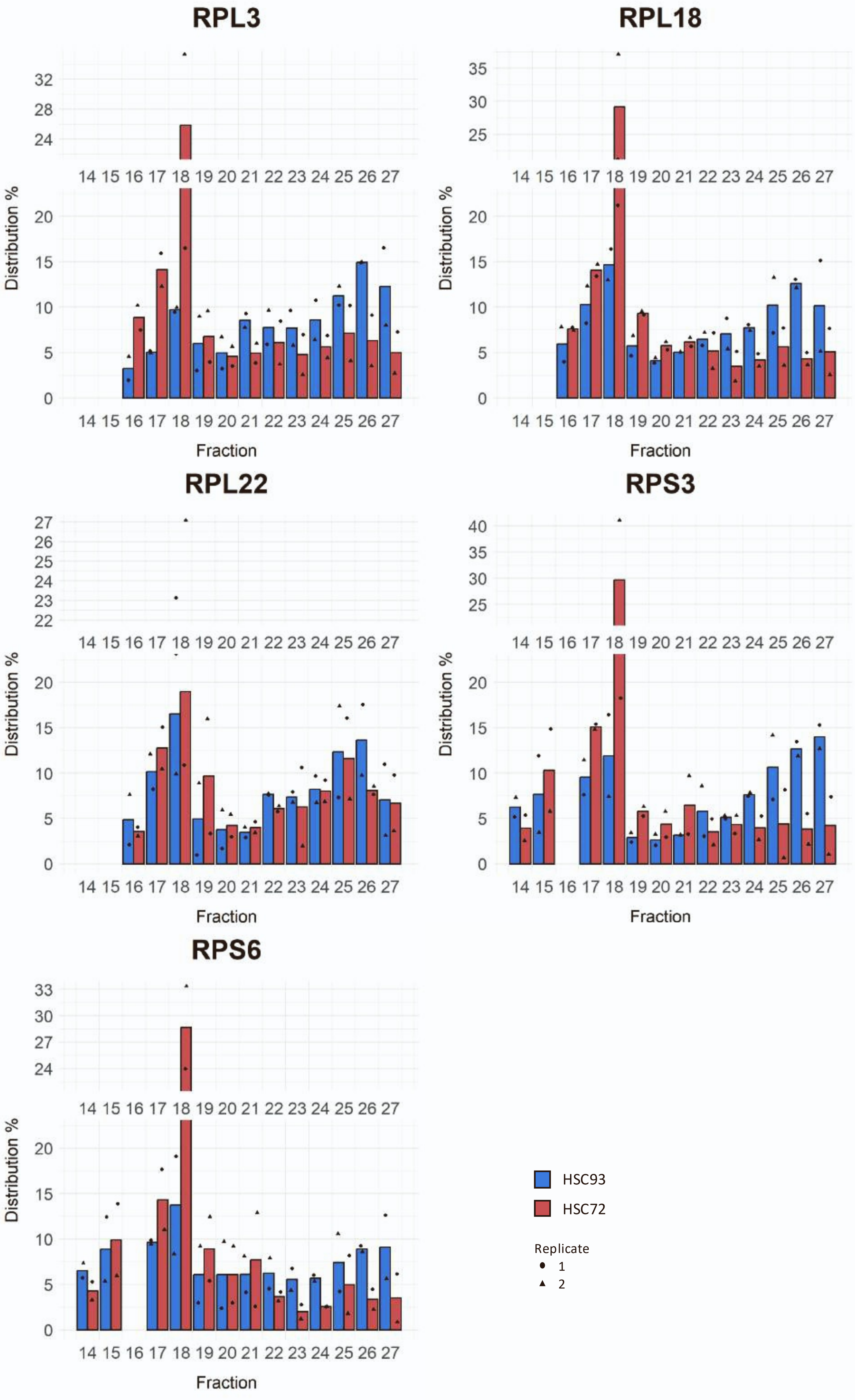

Supplemental Figure S3

A

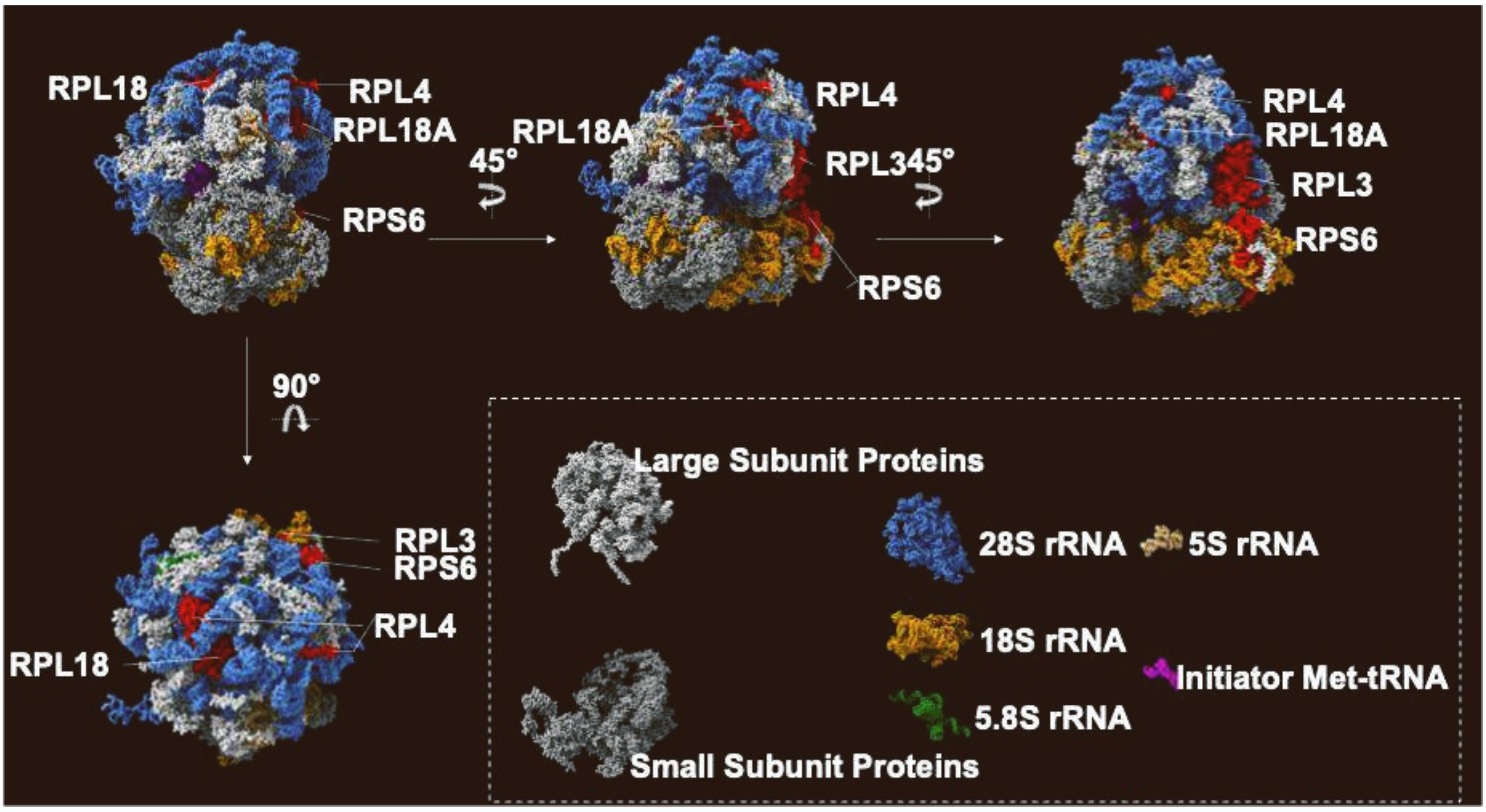

B

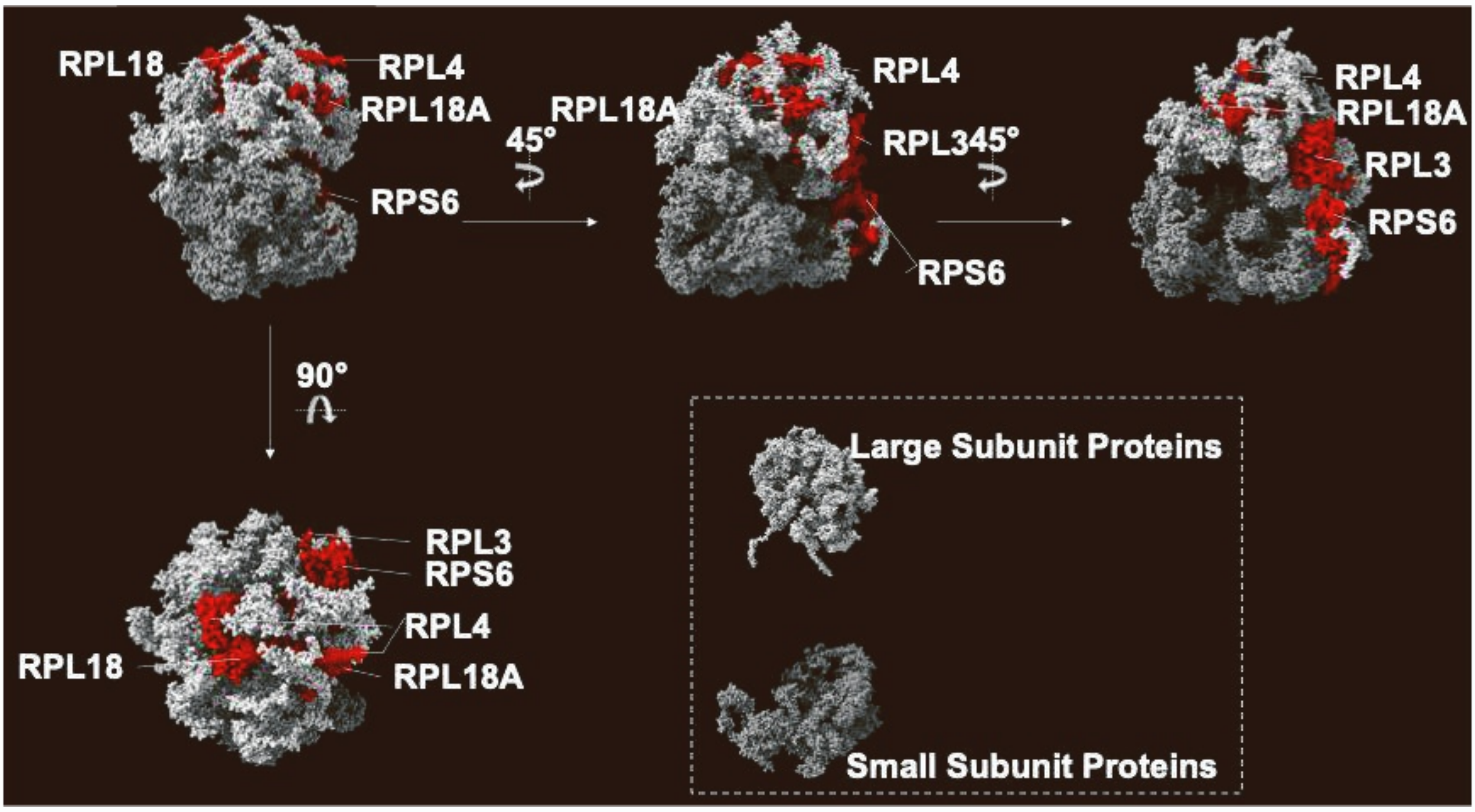

Supplemental Figure S4

A **Metapredict online (v3.0) (https://metapredict.net/) a deep-learning based consensus predictor of intrinsic disorder and predicted structure.**

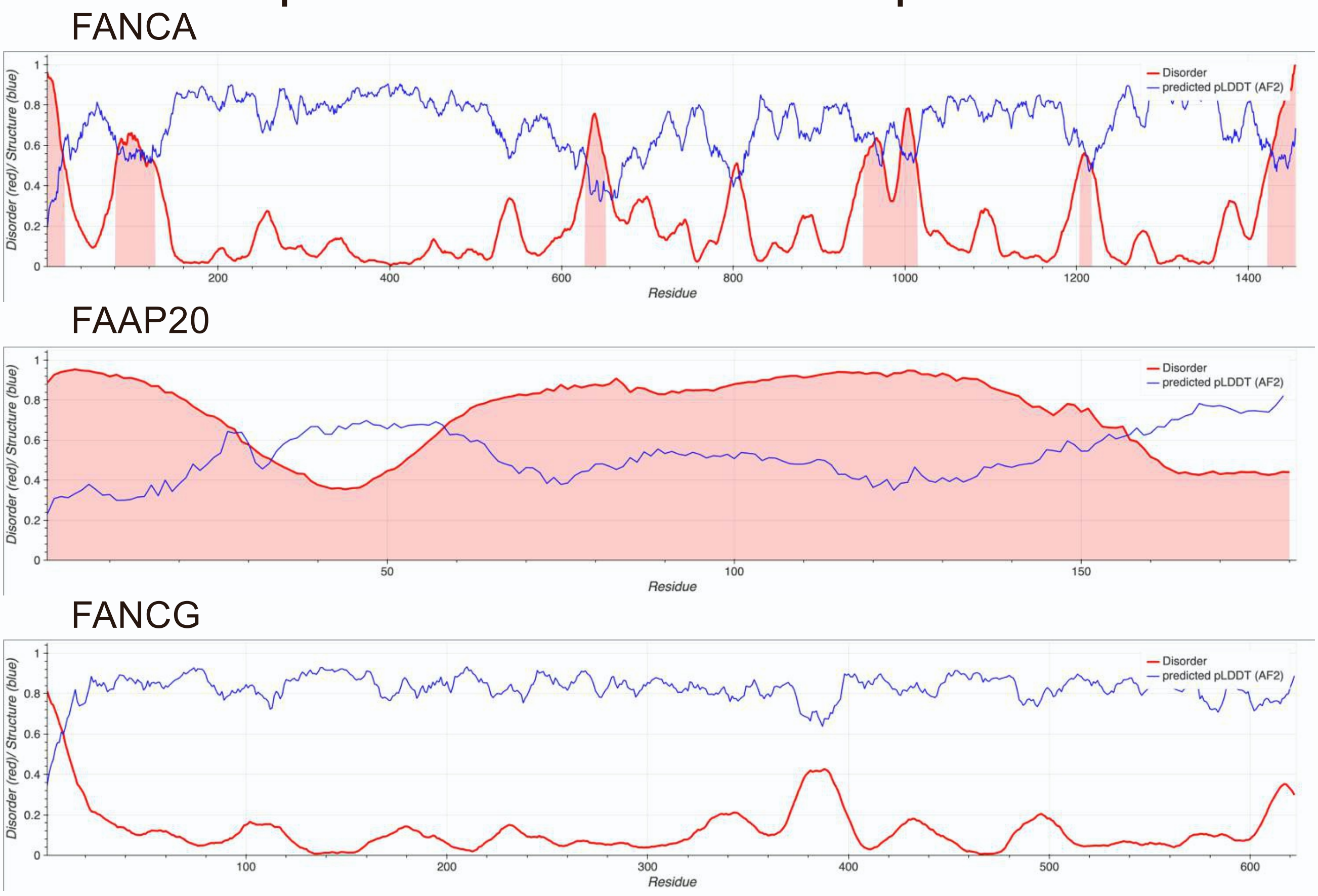

B **AIUPred - Prediction of Intrinsically Unstructured Proteins**
https://aiupred.elte.hu/

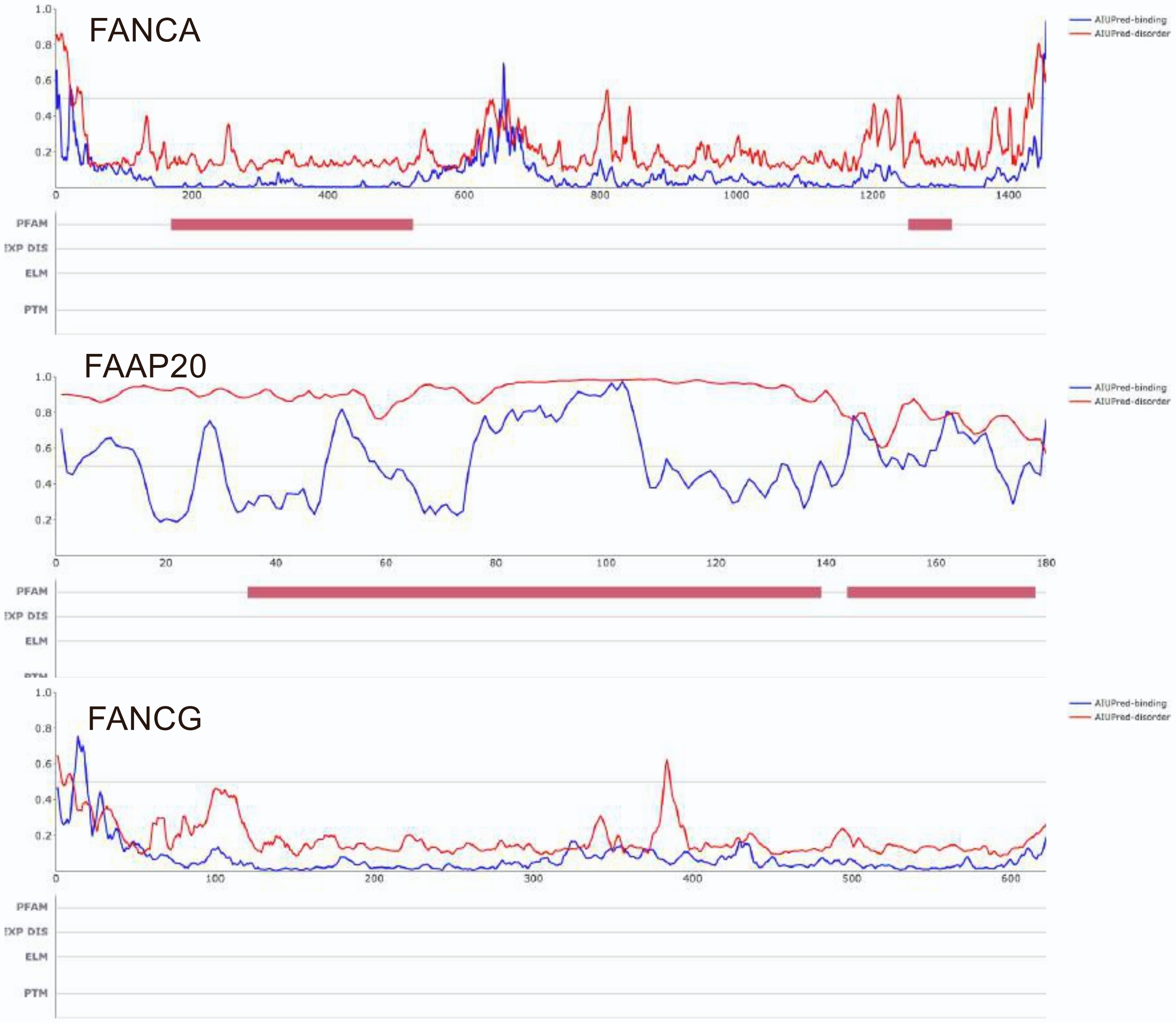

Supplemental Figure S5

C

DisorderEd PredictIon CenTER: DEPICTER2 WEB server data analysis for FANCA, FAAP20 and FANC(https://biomine.cs.vcu.edu/servers/DEPICTER/).

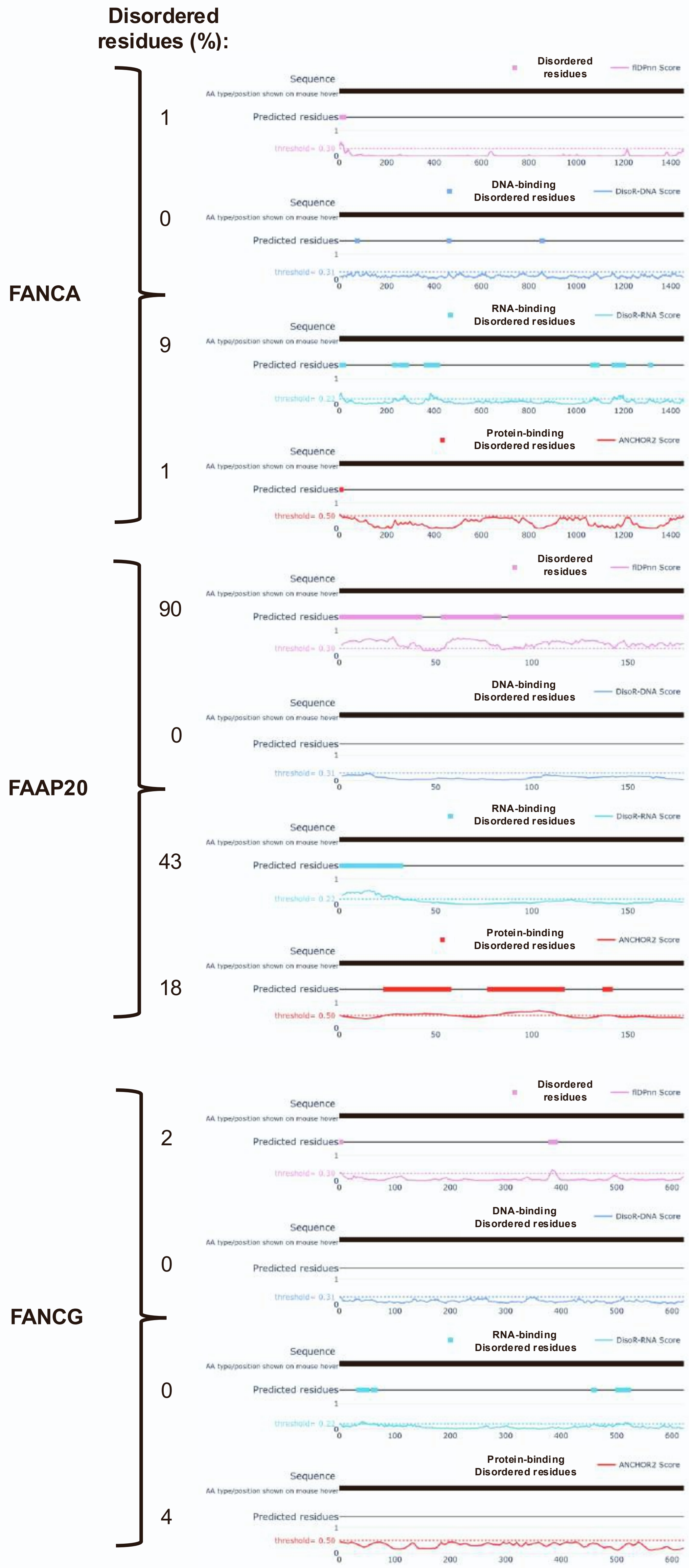

Supplemental Figure S5

Data S1

(The following 11 pages present the original blots of the panels in Figure 2 and Figure 7B-C)

The Molecular Weight of the analyzed proteins in figure 2:

	Apparent MW kDa
FANCA	160
FANCG	69
FANCD2	164
NCL	100
NPM1	37
RPL18	22
INVS1ABP	71
NF45	45
NF90	90
NF110	110
LIG4	100
XRCC4	55
CUX1	200
RAE1	44

Hybridization order →
NCL, NPM,1 FANCG & FANCA

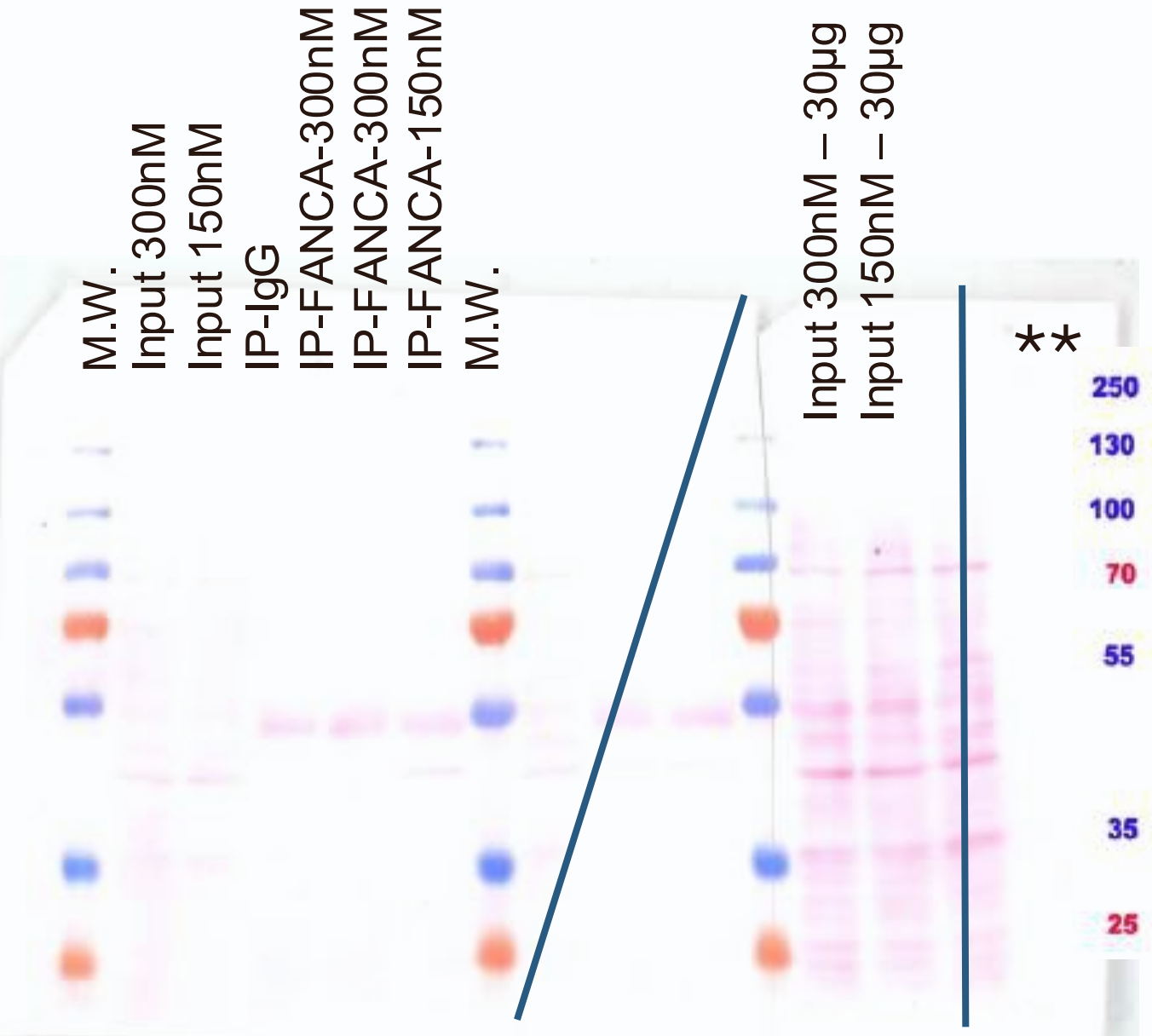

Note that the membrane was not stripped between successive hybridizations. Consequently, signals corresponding to previously probed proteins may still be visible in subsequent hybridizations. For example, signals for NCL and NPM1 can be observed in the FANCG hybridization.

The regions shown in the main figure are indicated by frames. The exposure selected for the main figure is not necessarily the same as that presented here. Images were acquired using an imaging system that records multiple exposure times, allowing selection of the exposure best suited for presentation without altering the image itself.

The amount of protein loaded in the input lanes on the left was insufficient to clearly visualize the FANCA and FANCG bands. This is evident from the corresponding input lanes shown on the right, where increased loading and higher exposure reveals their presence.

For completeness, we provide below overexposed images that demonstrate the presence of FANCA and FANCG in the input samples. However, the quality of these images is not sufficient for inclusion in the main figure.

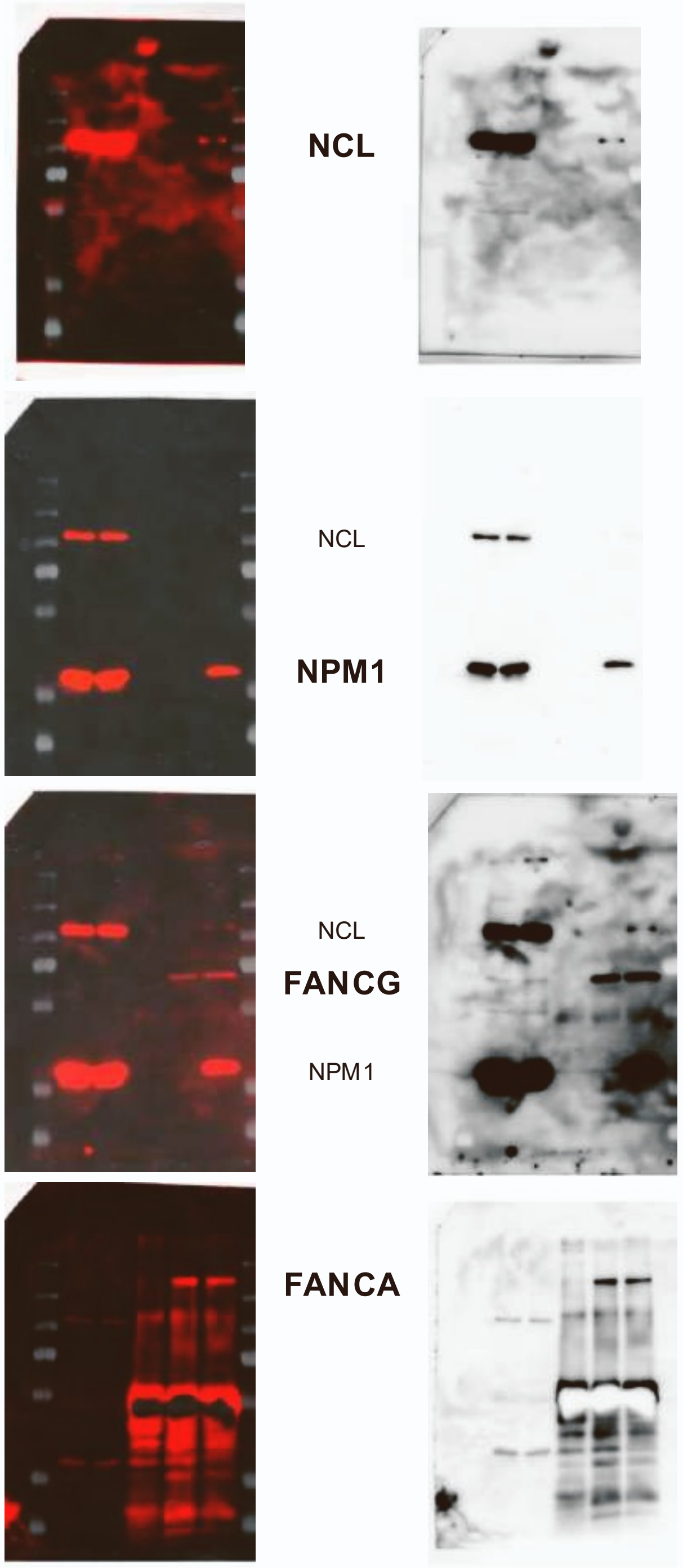

FANCA

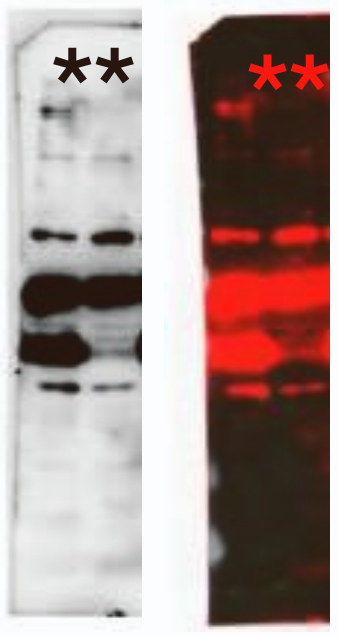

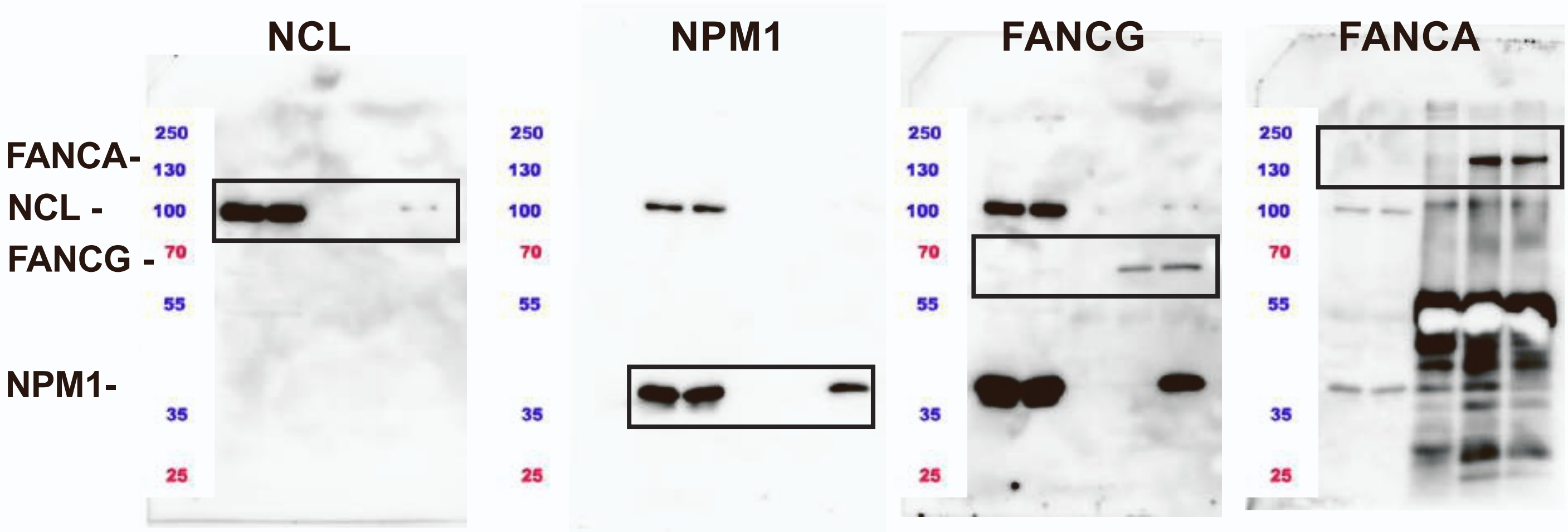

For FIGURE 2A

Hybridization order →
NCL, NPM1, FANCG & FANCA.

For FANCA and RPL18, the membrane was cut prior to hybridization to reduce the volume of antibody incubation buffer required and to allow hybridization with both antibodies to be performed in parallel on the same day.

The regions shown in the main figure are indicated by frames. The exposure selected for the main figure is not necessarily the same as that presented here. Images were acquired using an imaging system that records multiple exposure times, allowing selection of the exposure best suited for presentation without altering the image itself.

For NCL and RPL18, two different exposure times are shown.

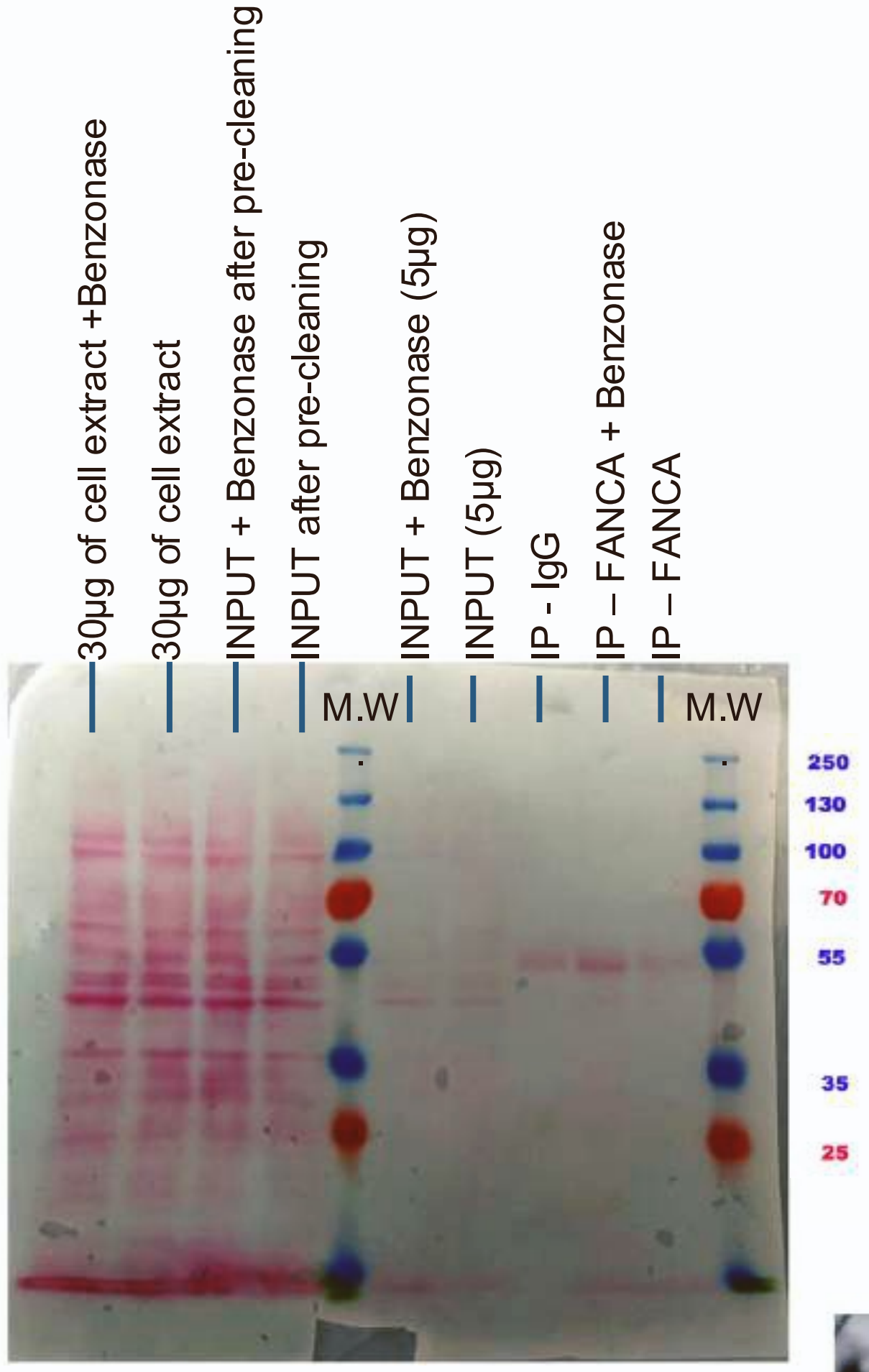

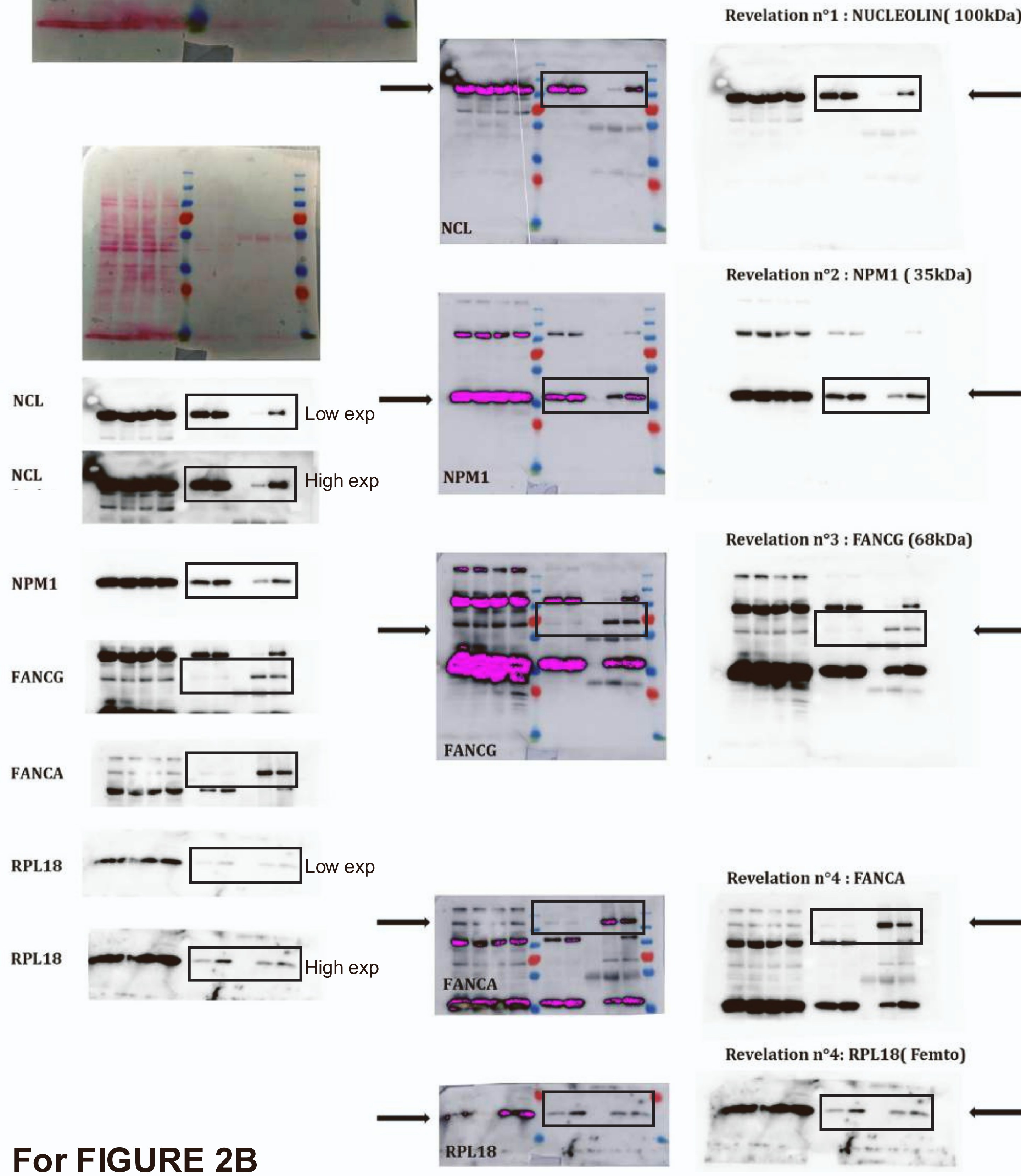

For FIGURE 2B

The immunoprecipitate (IP) was divided into two equal fractions and loaded onto two separate gels.

The first gel (bottom, left) was hybridized with anti-FANCA and anti-INVS1ABP antibodies. The second gel (top, right) was hybridized with anti-FANCA and anti-FANCG antibodies.

For Figure 2C, the INVS1ABP signal was taken from the first gel, whereas the FANCA and FANCG signals were taken from the second gel.

The FANCA hybridization is shown for both membranes, allowing readers to assess the consistency of the results across the two gels.

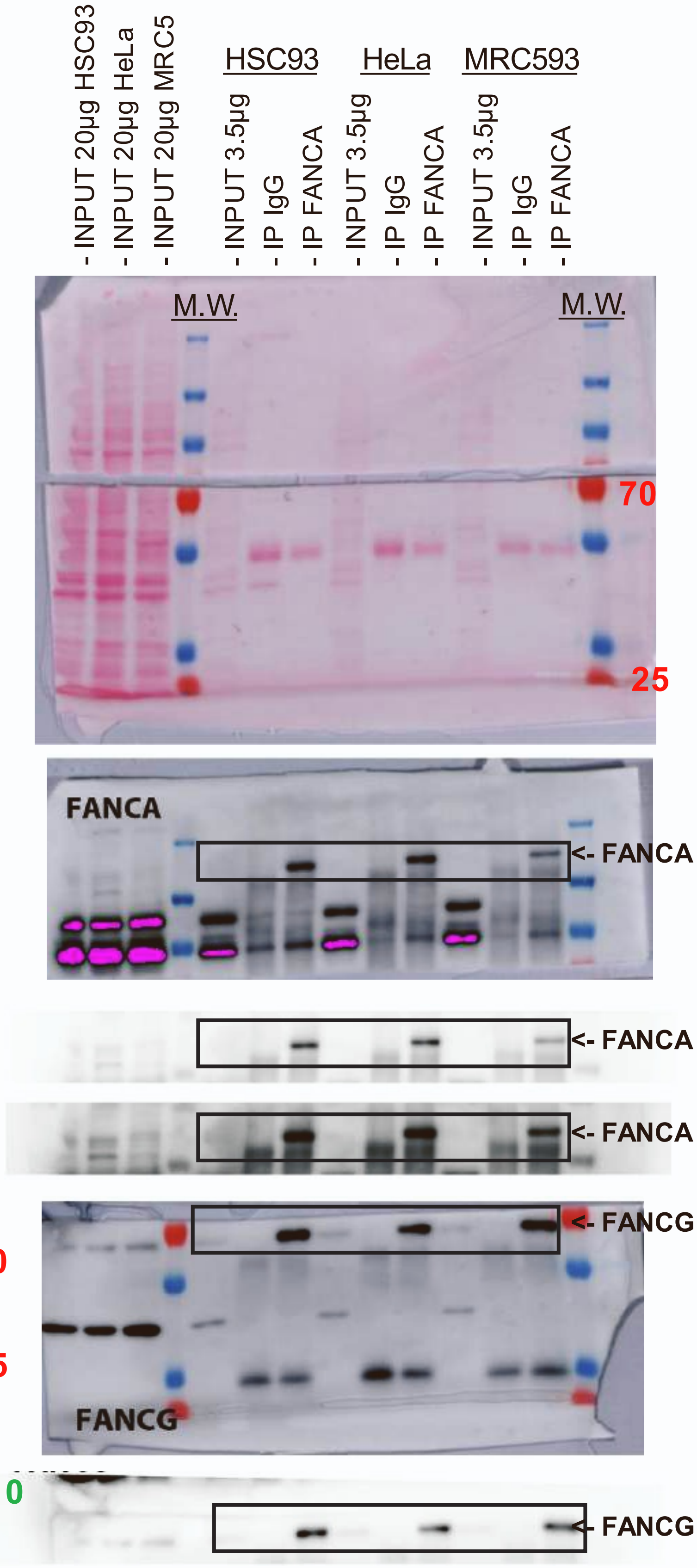

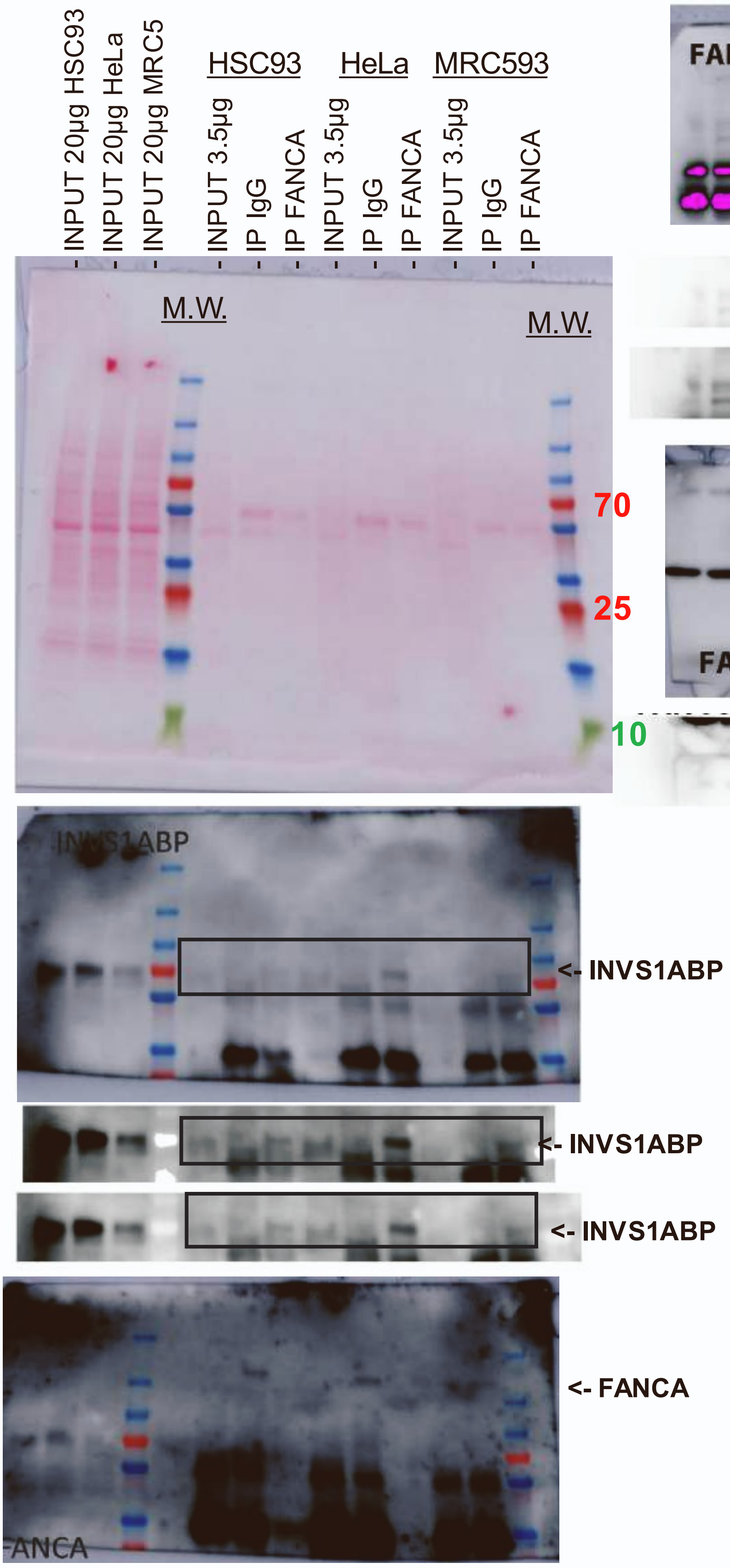

For FIGURE 2C

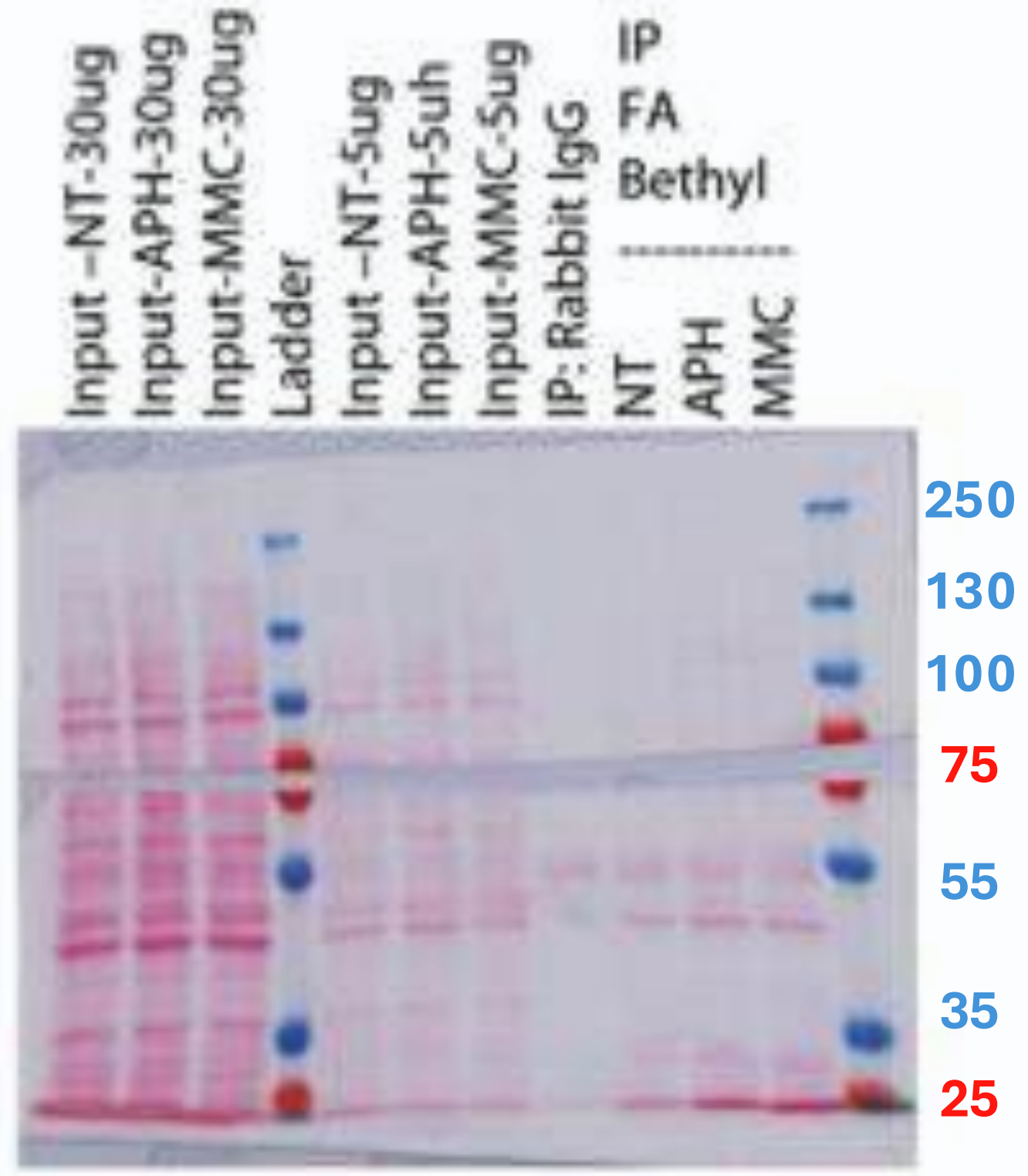

After transfer, the membrane was cut into two sections. The upper portion was used for hybridization with anti-FANCA and anti-NF90/NF110 antibodies, whereas the lower portion was used for hybridization with the anti-NF45 antibody.

Different exposure times were selected for each antibody to optimize signal visualization.

For each antibody, the image shown corresponds to the exposure time that provided the most appropriate signal visualization.

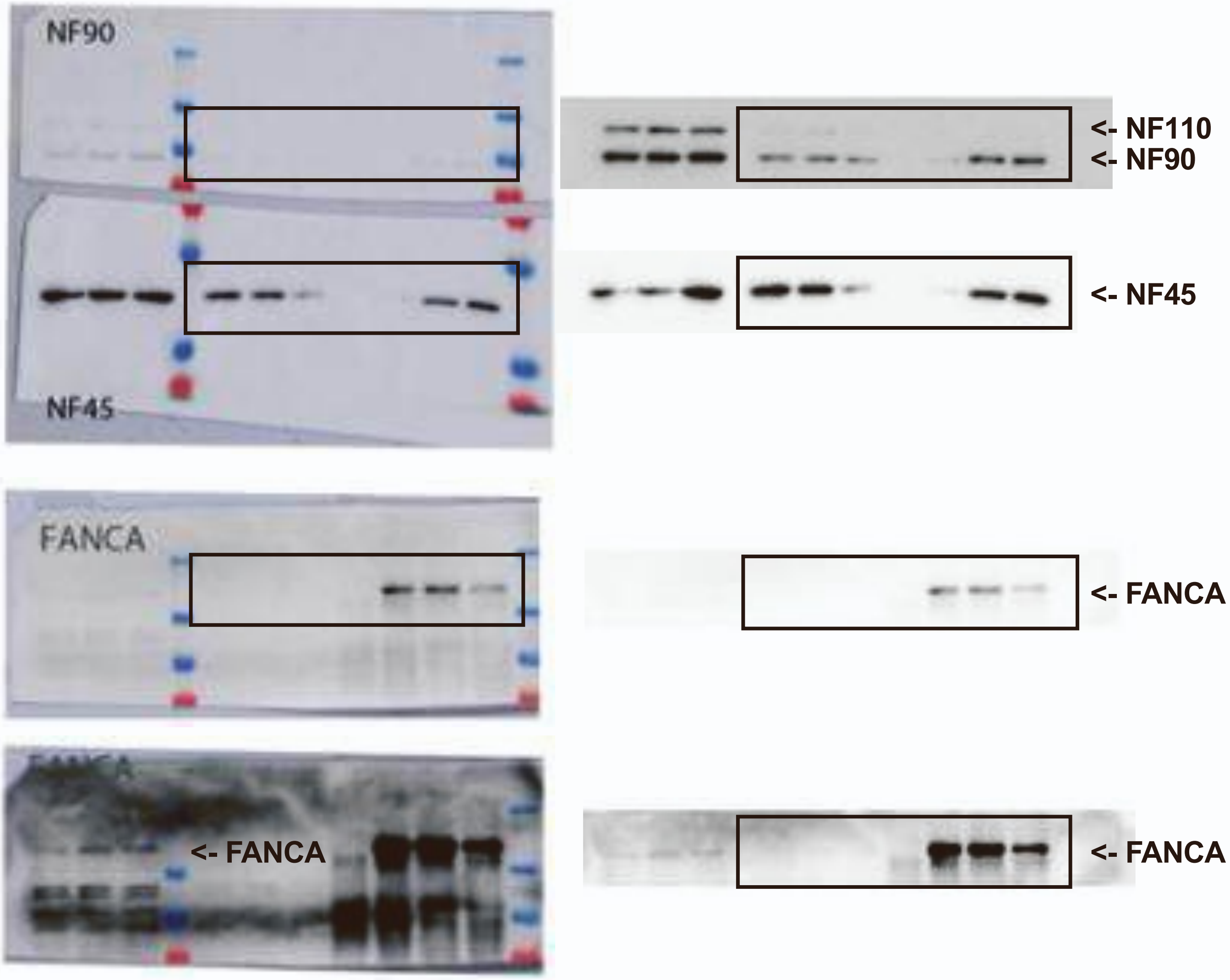

For FIGURE 2D

As indicated above, images were acquired using an imaging system that records multiple exposure times, allowing selection of the exposure best suited for presentation without any alteration of the original image.

Below are examples of different exposure times obtained from the same blot using the same antibody.

Following transfer, the original membrane was cut into two sections. On the upper membrane, LIG4 (~100 kDa), FANCA (~160 kDa), and CUX1 (~200 kDa) were detected sequentially in the order listed.

On the lower membrane, XRCC4 (~55 kDa), FANCG (~70 kDa), and RAE1 (~40 kDa) were detected sequentially in the order listed.

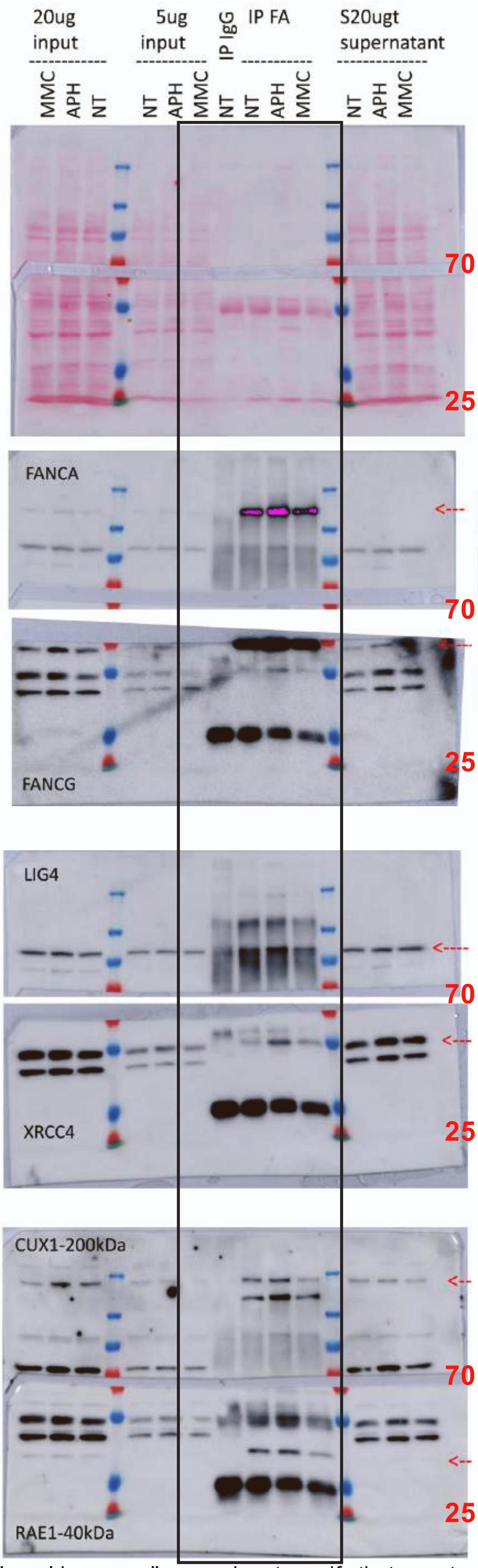

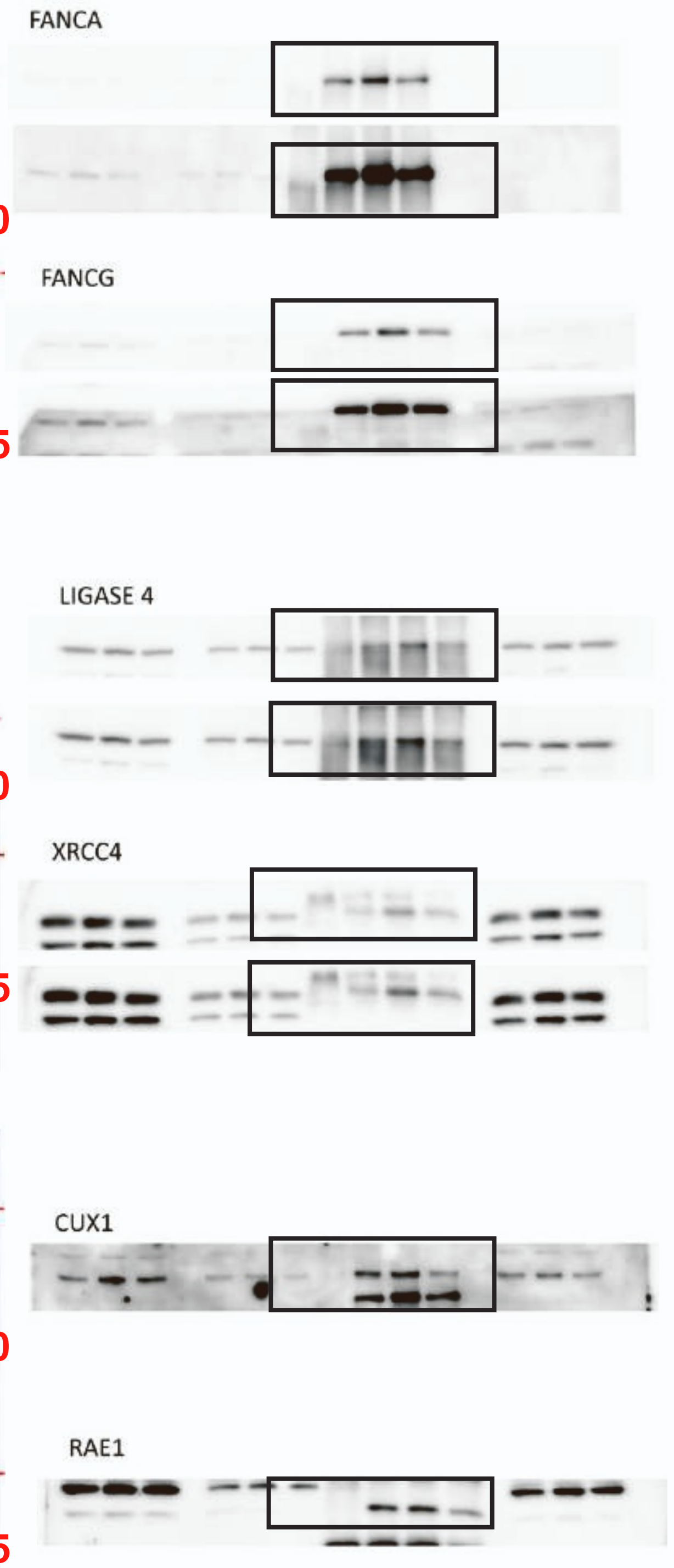

Whole-gel images allow readers to verify that genotoxic treatment does not appreciably alter the expression levels of the analyzed proteins, with the possible exception of CUX1.

Because inclusion of an input lane is essential to demonstrate the presence of each protein in the extract, while avoiding unnecessary complexity in the main figure, we chose to present the input from MMC-treated cells in the final figure to facilitate protein identification. The complete images shown here include the input lanes for all extracts, thereby providing full information to the reader.

Importantly, restricting the data displayed in the main figure to the framed regions neither removes relevant information nor introduces additional information; it simply focuses the presentation on the portions necessary to illustrate the reported results.

*** The "supernatant" lanes correspond to proteins that were not co-immunoprecipitated with FANCA and therefore remained in the post-immunoprecipitation fraction.

For FIGURE 2E

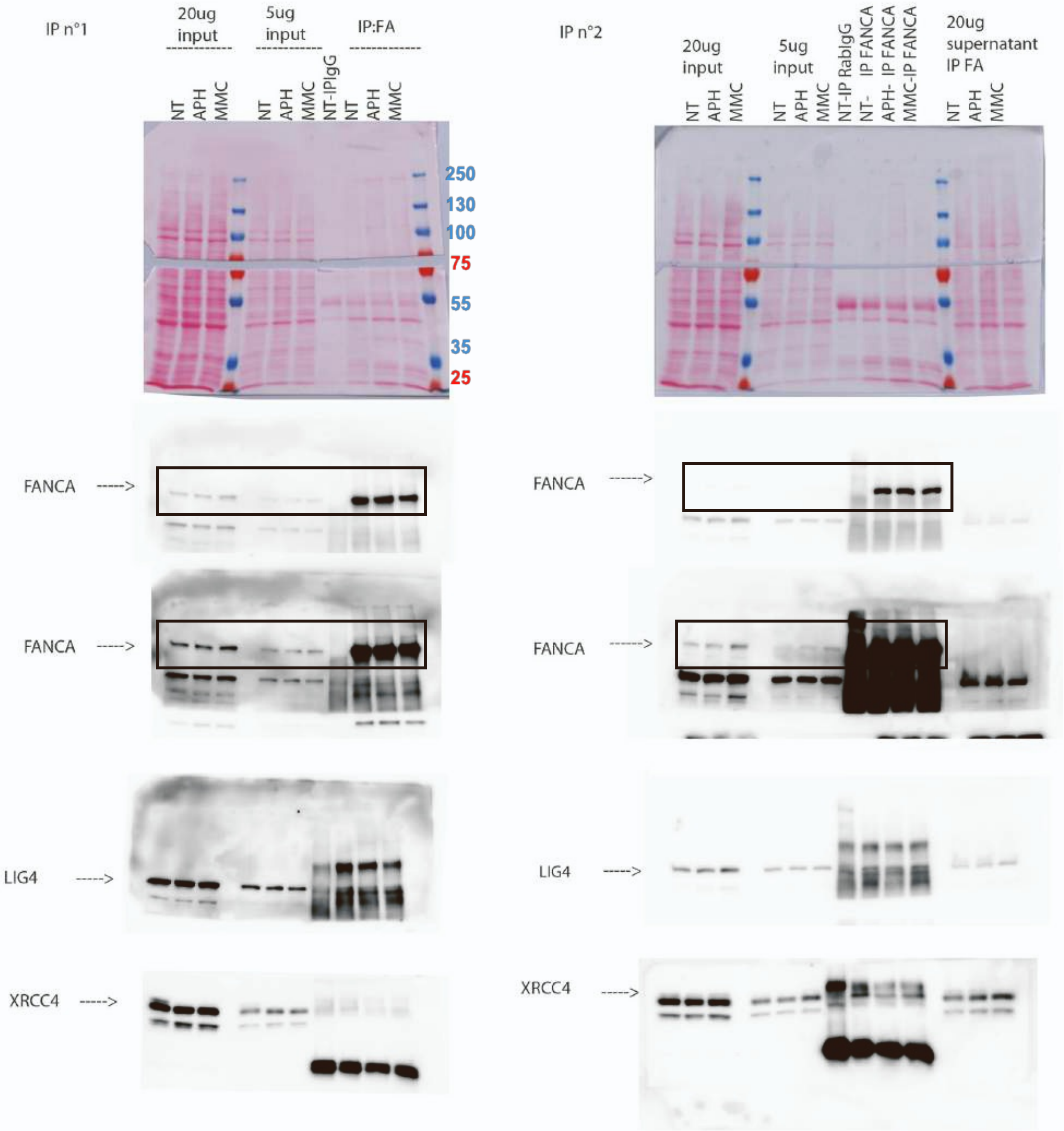

Following transfer, the original membrane was cut into two sections. On the upper membrane, LIG4 (~100 kDa) and FANCA (~160 kDa) were detected sequentially in the order.

On the lower membrane, XRCC4 (~55 kDa) were.

For completeness, overexposed images are shown to allow visualization of the FANCA signal in the input samples, that is difficult to visualize in the main figure.

For FIGURE 2F

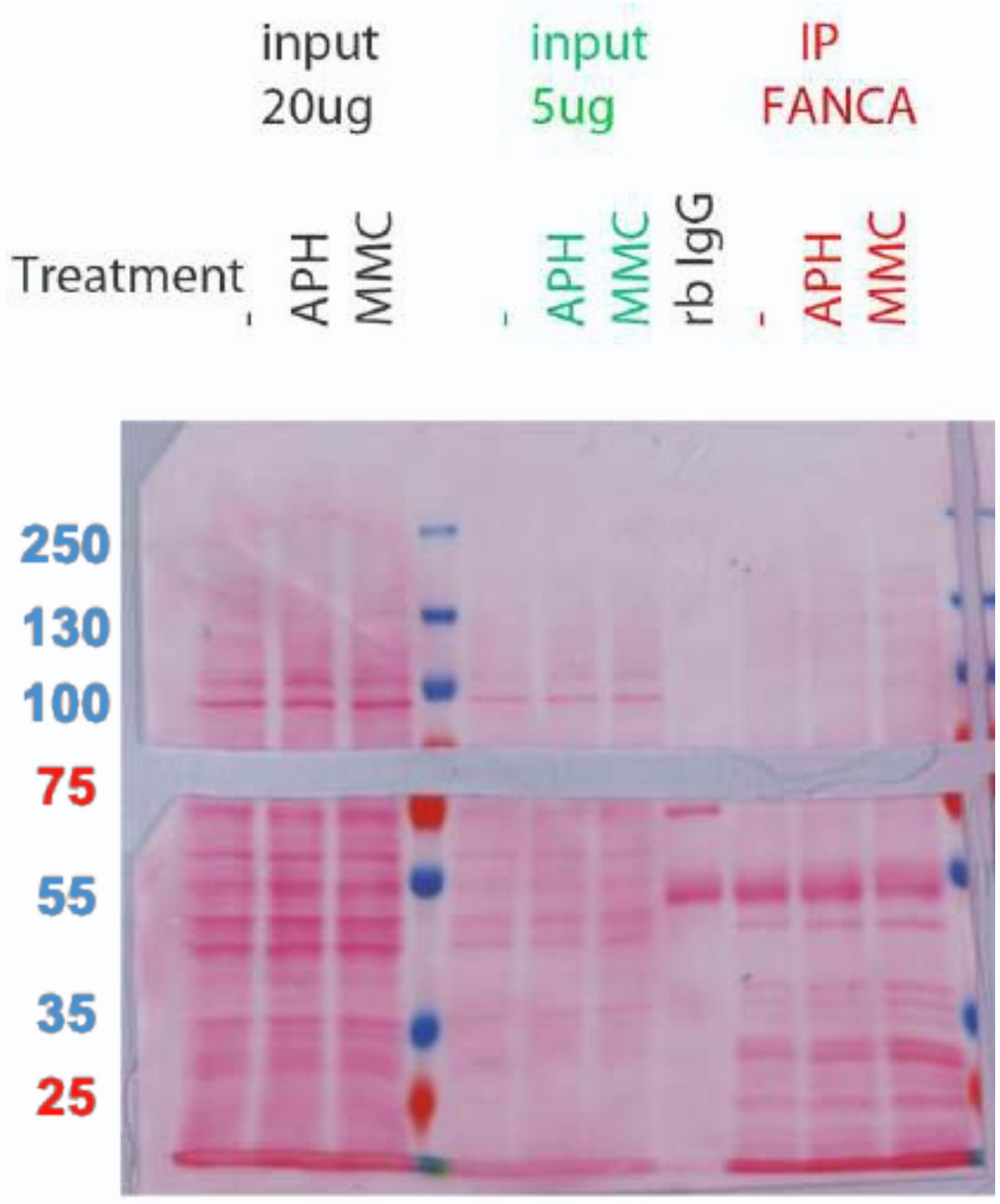

LIG4 and FANCA were detected sequentially on the upper membrane, in that order, without stripping the membrane between successive hybridizations.

For completeness, an overexposed image is included to enable visualization of the FANCA signal in the input samples.

XRCC4 was detected on the lower membrane.

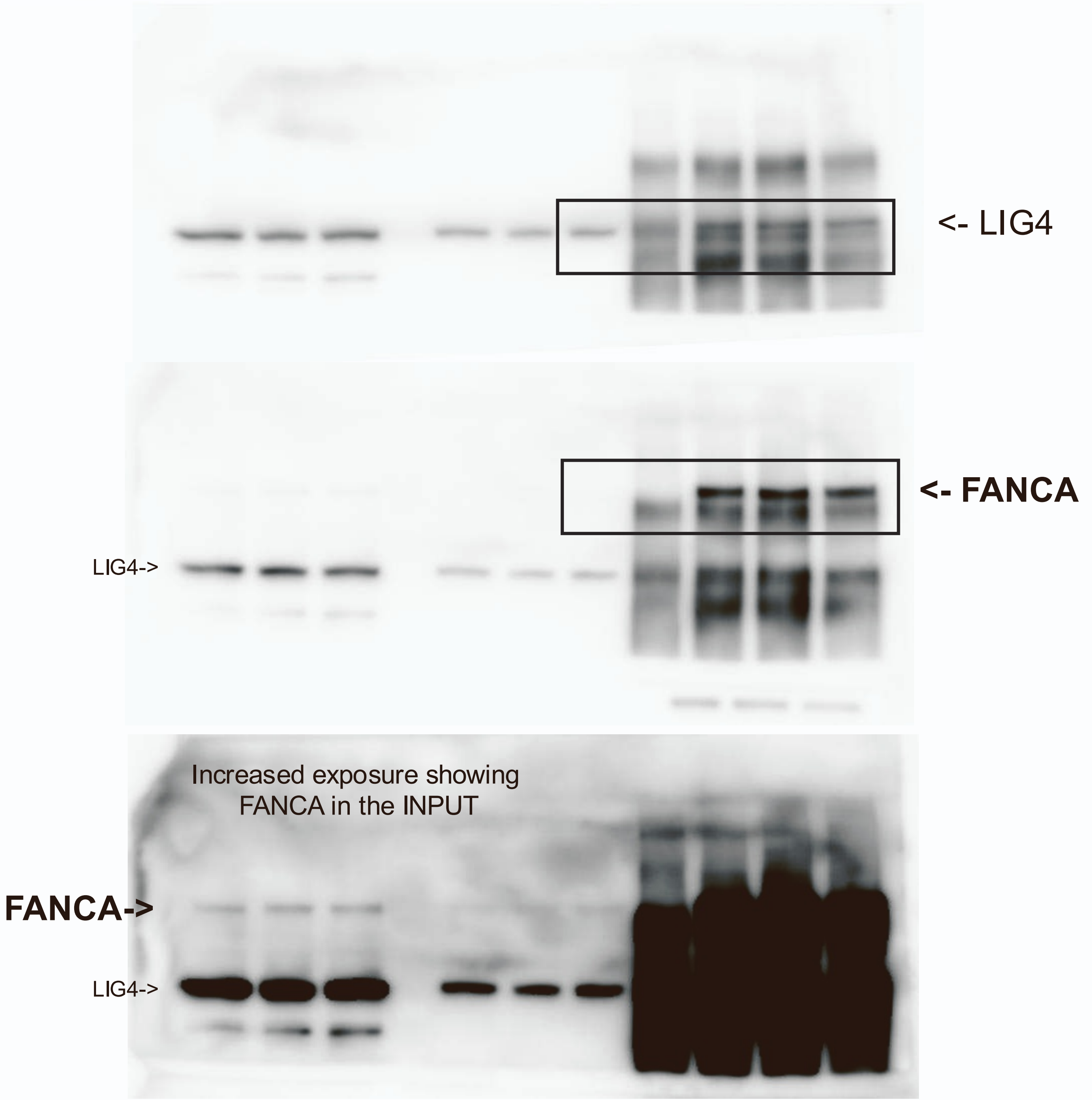

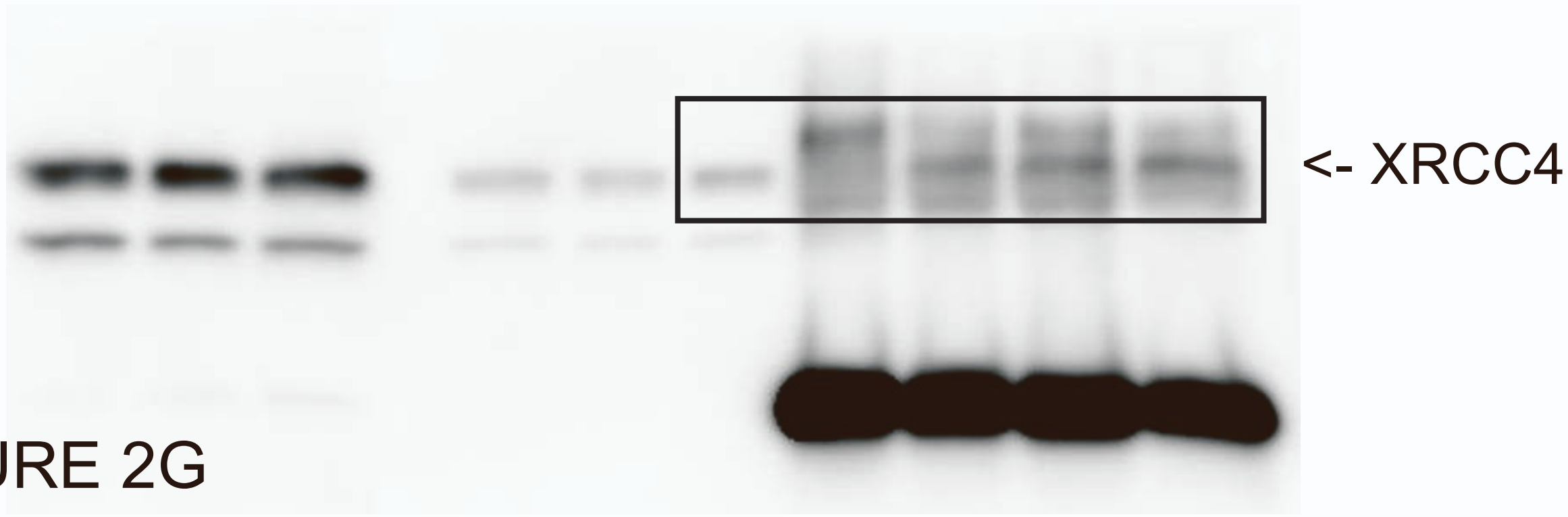

For FIGURE 2G

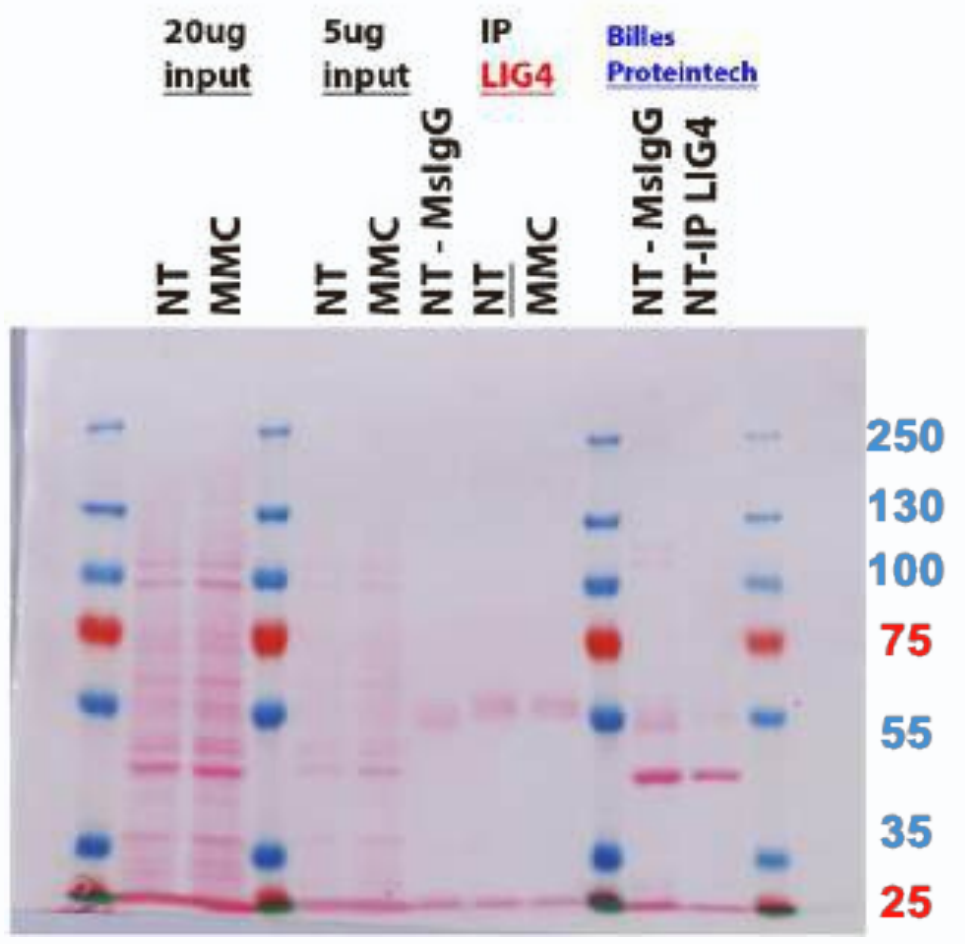

Hybridization order → FANCA, LIG4, XRCC4

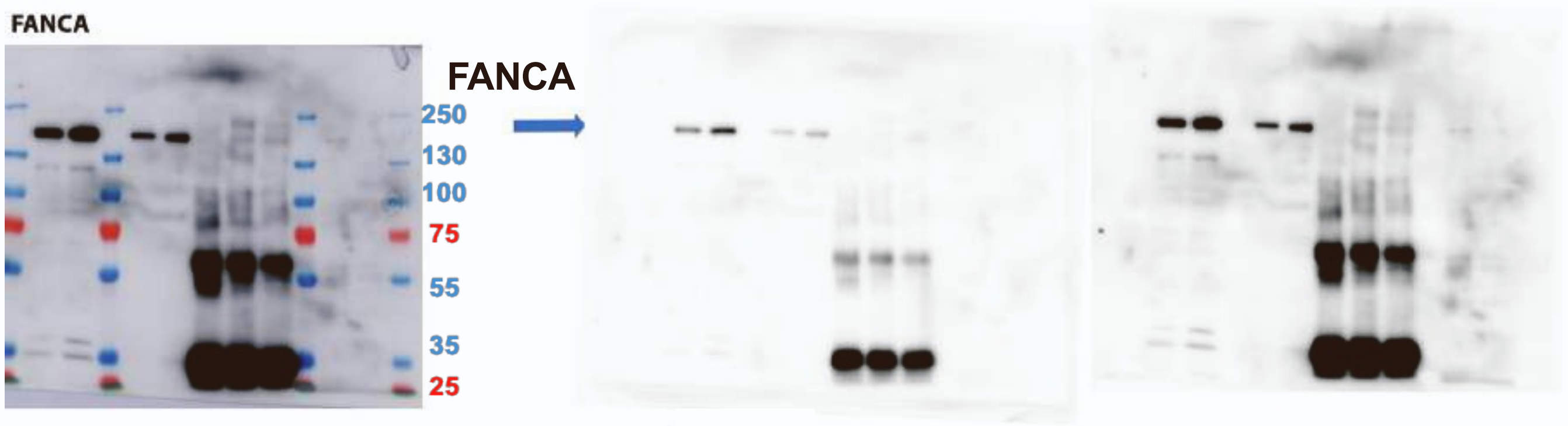

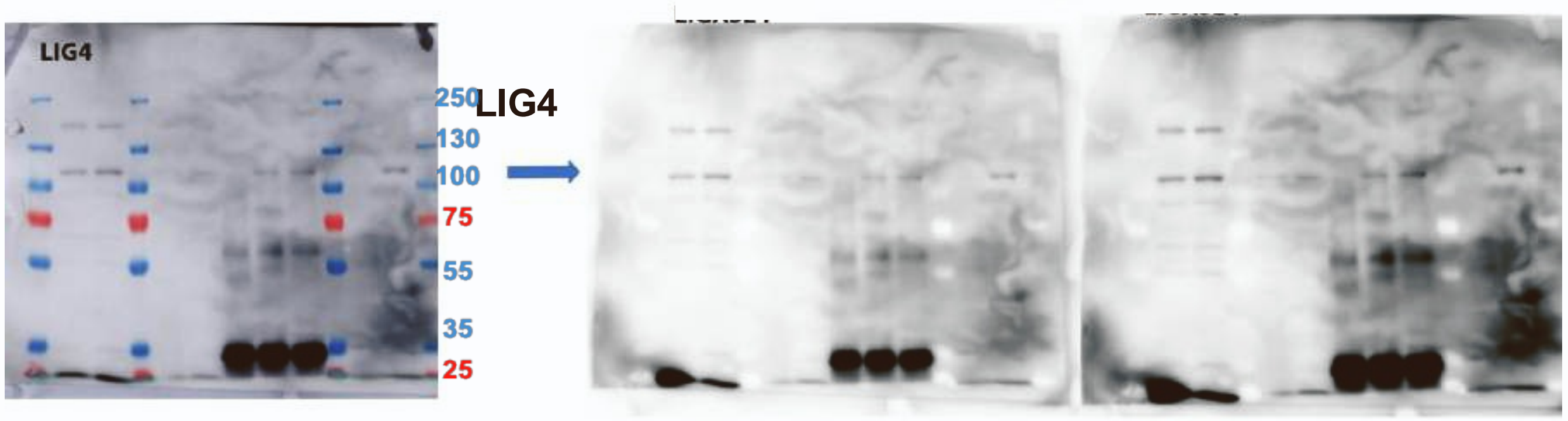

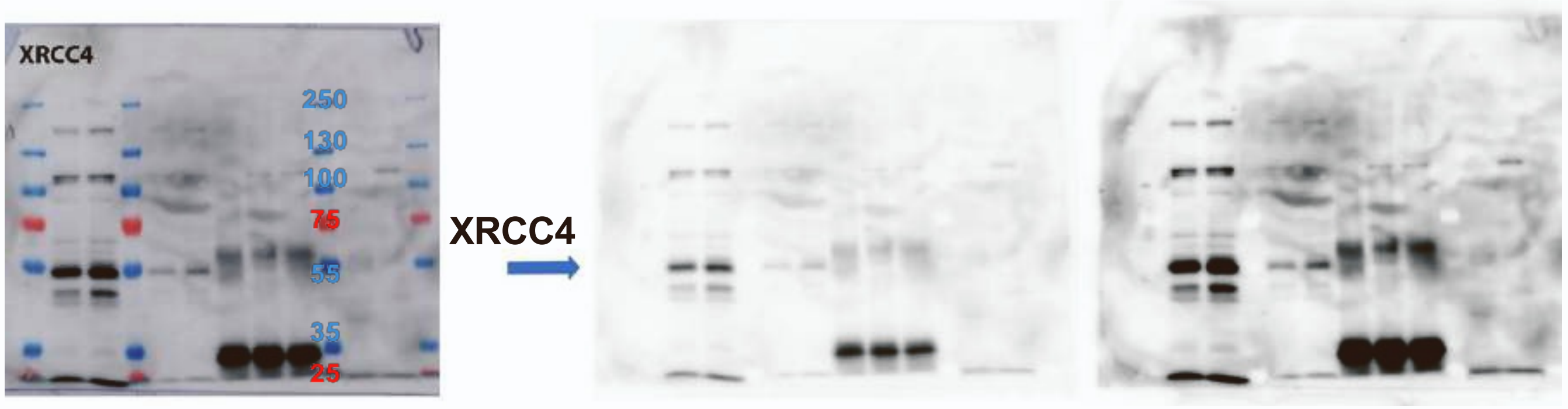

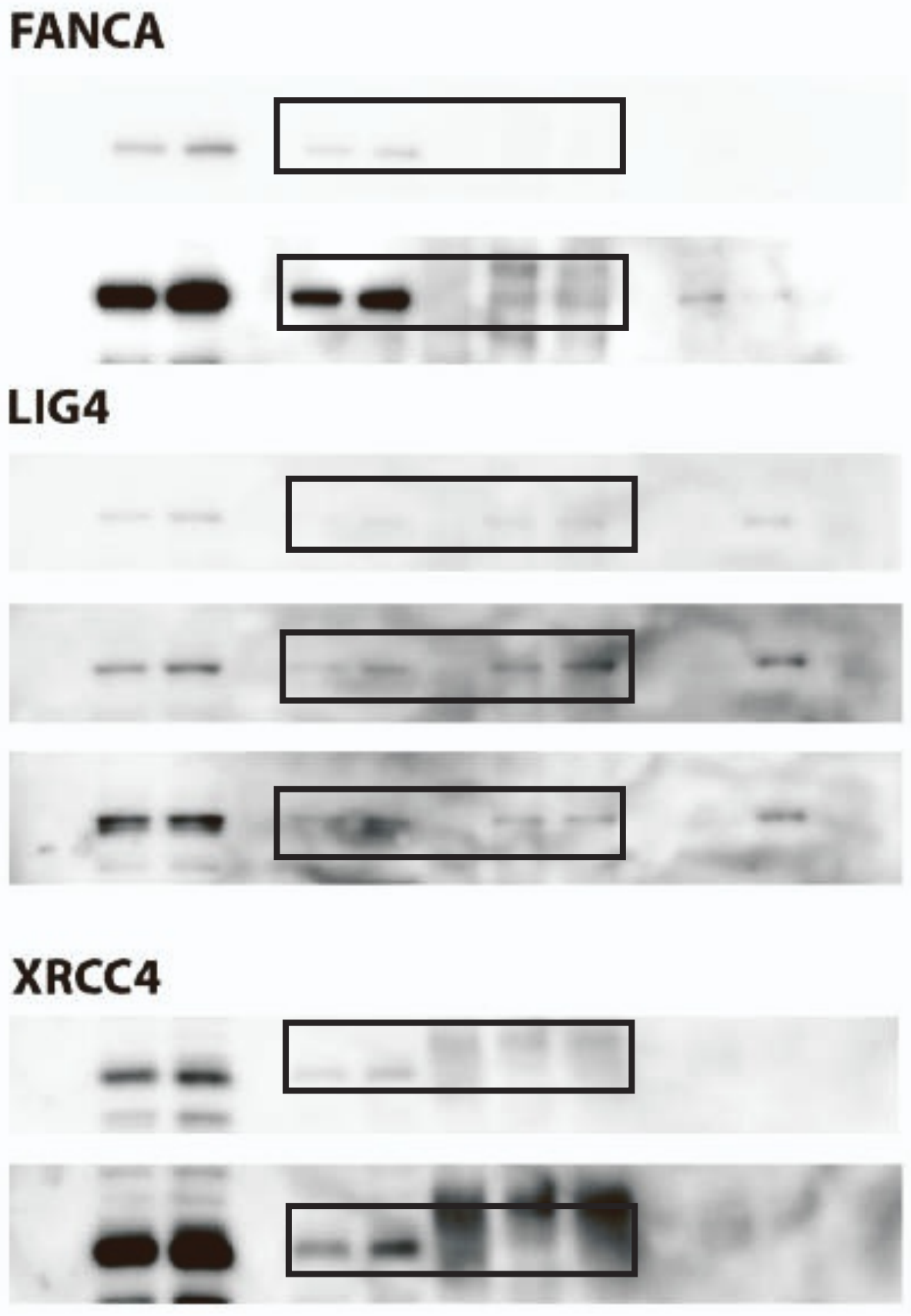

The regions shown in the main figure are indicated by frames. The exposure selected for the main figure is not necessarily the same as that presented here. Images were acquired using an imaging system that records multiple exposure times, allowing selection of the exposure best suited for presentation without altering the image itself

For FIGURE 2H

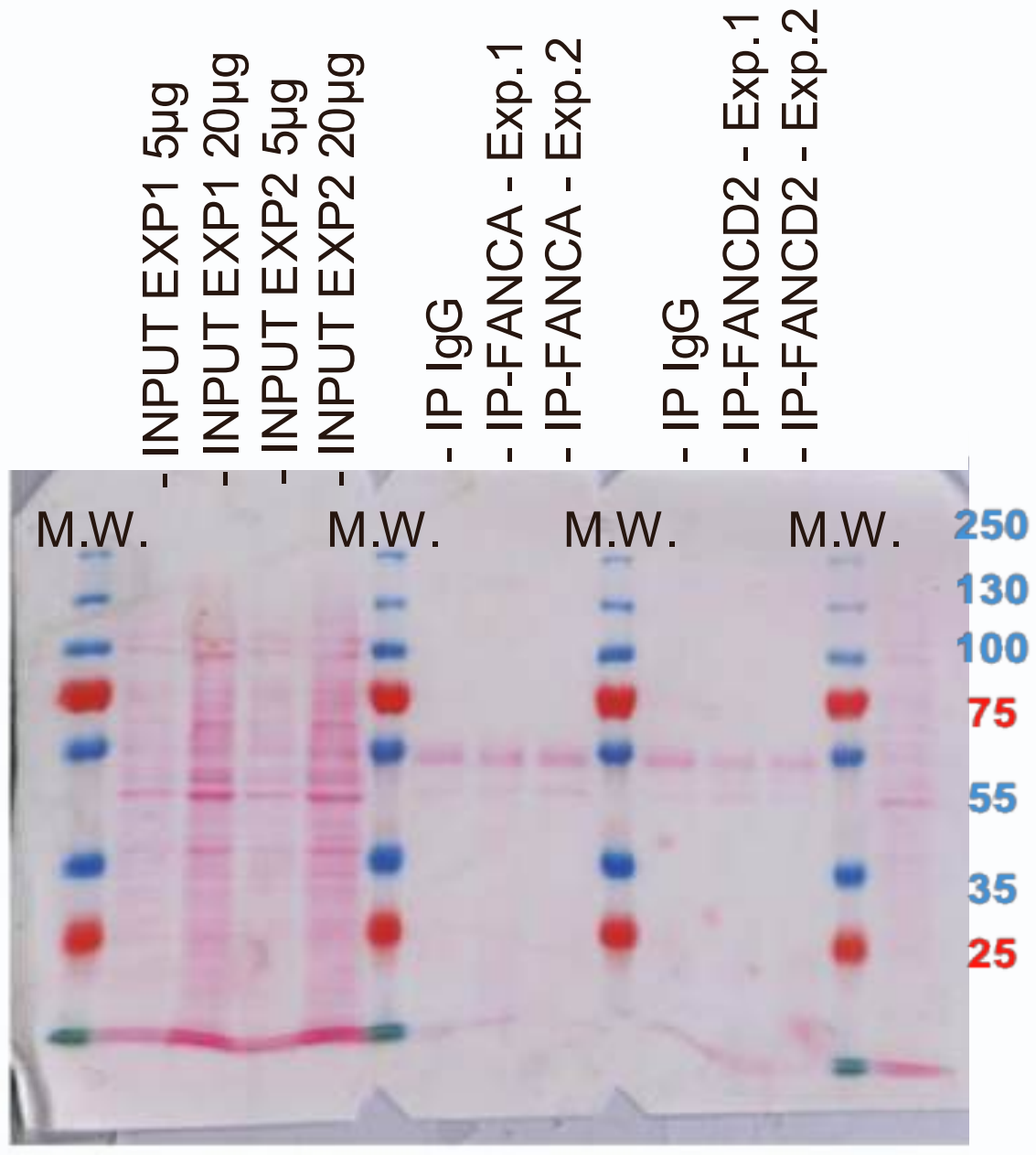

First hybridization: NCL

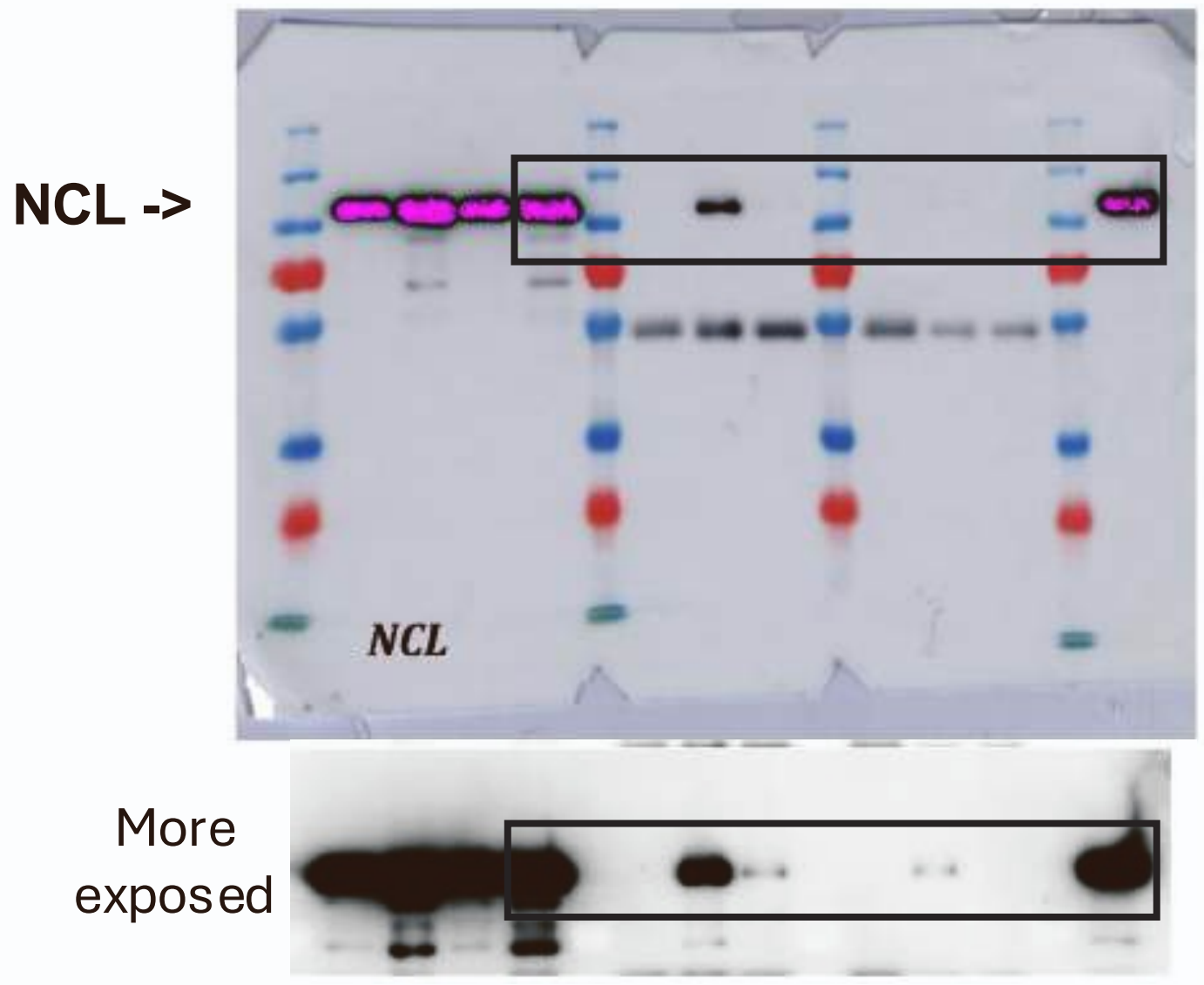

Second hybridization: NPM1

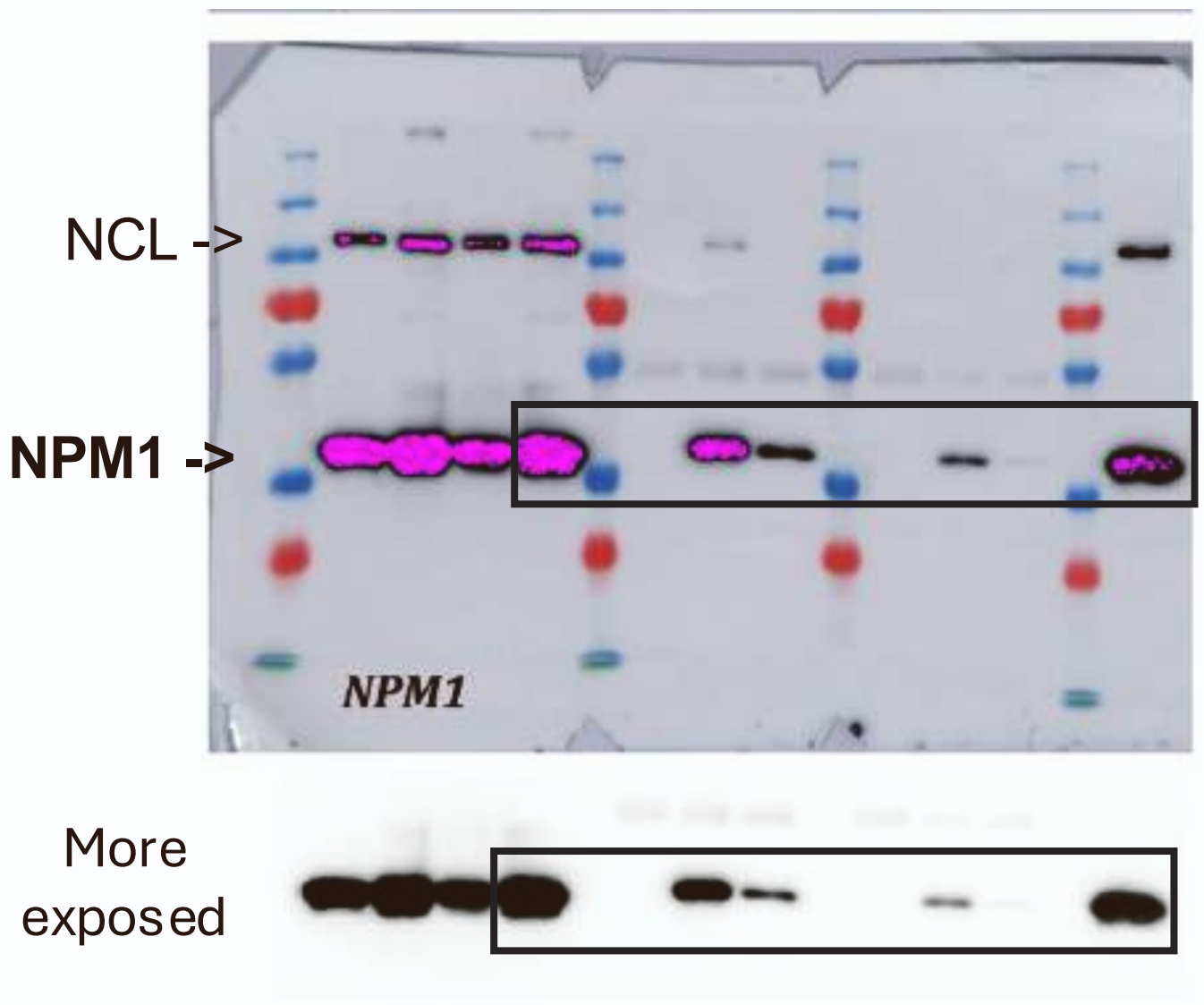

Third hybridization: FANCG

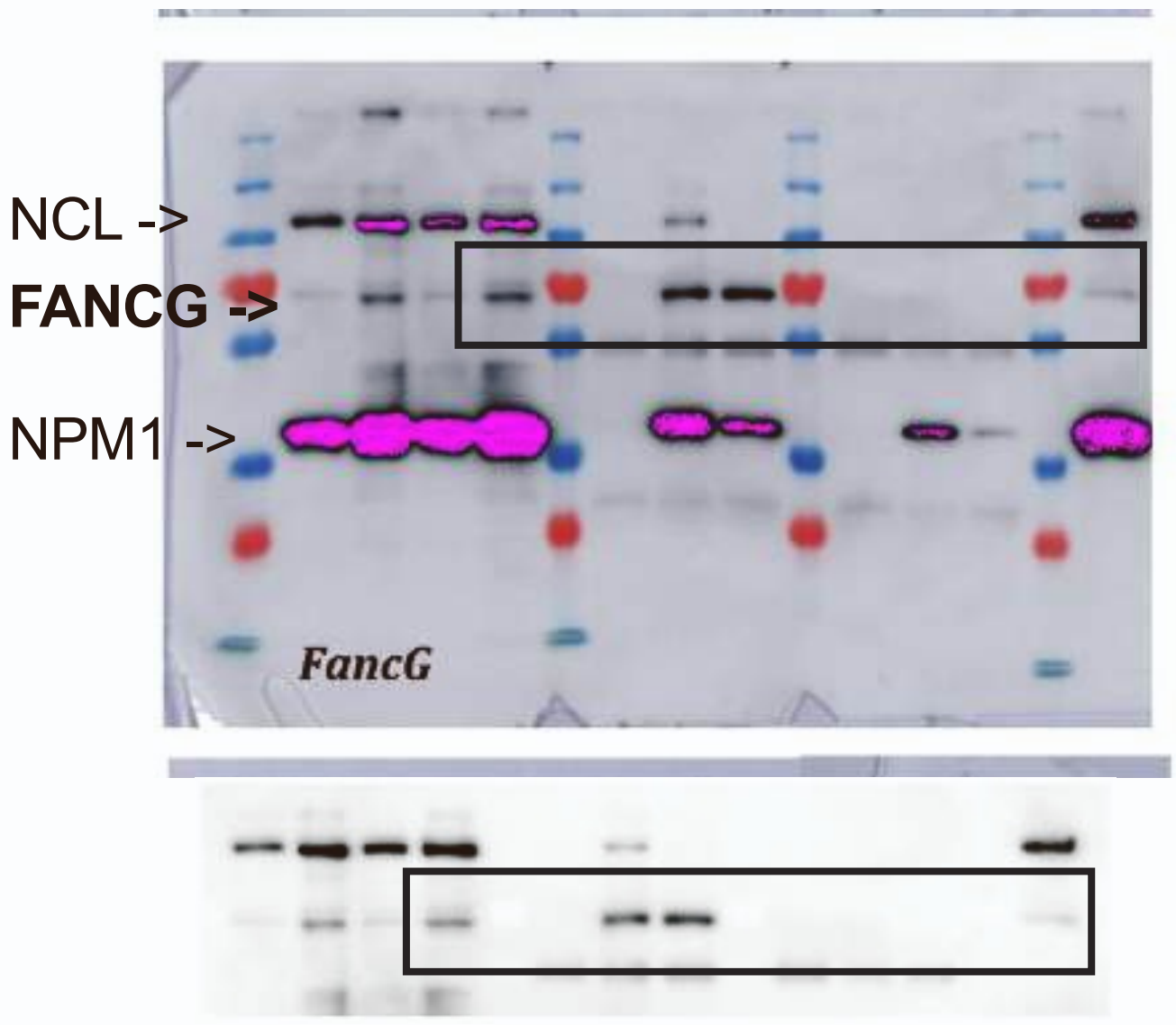

Next, the original membrane was cut vertically to reveal FANCA and FANCD2 (similar M.W) on the two alternative parts of the membrane.

Following this initial vertical separation, the membrane was further cut horizontally to allow detection of RPL18. The two resulting RPL18 membranes (corresponding to the FANCA- and FANCD2-associated samples) were subsequently hybridized together in the same buffers and developed simultaneously.

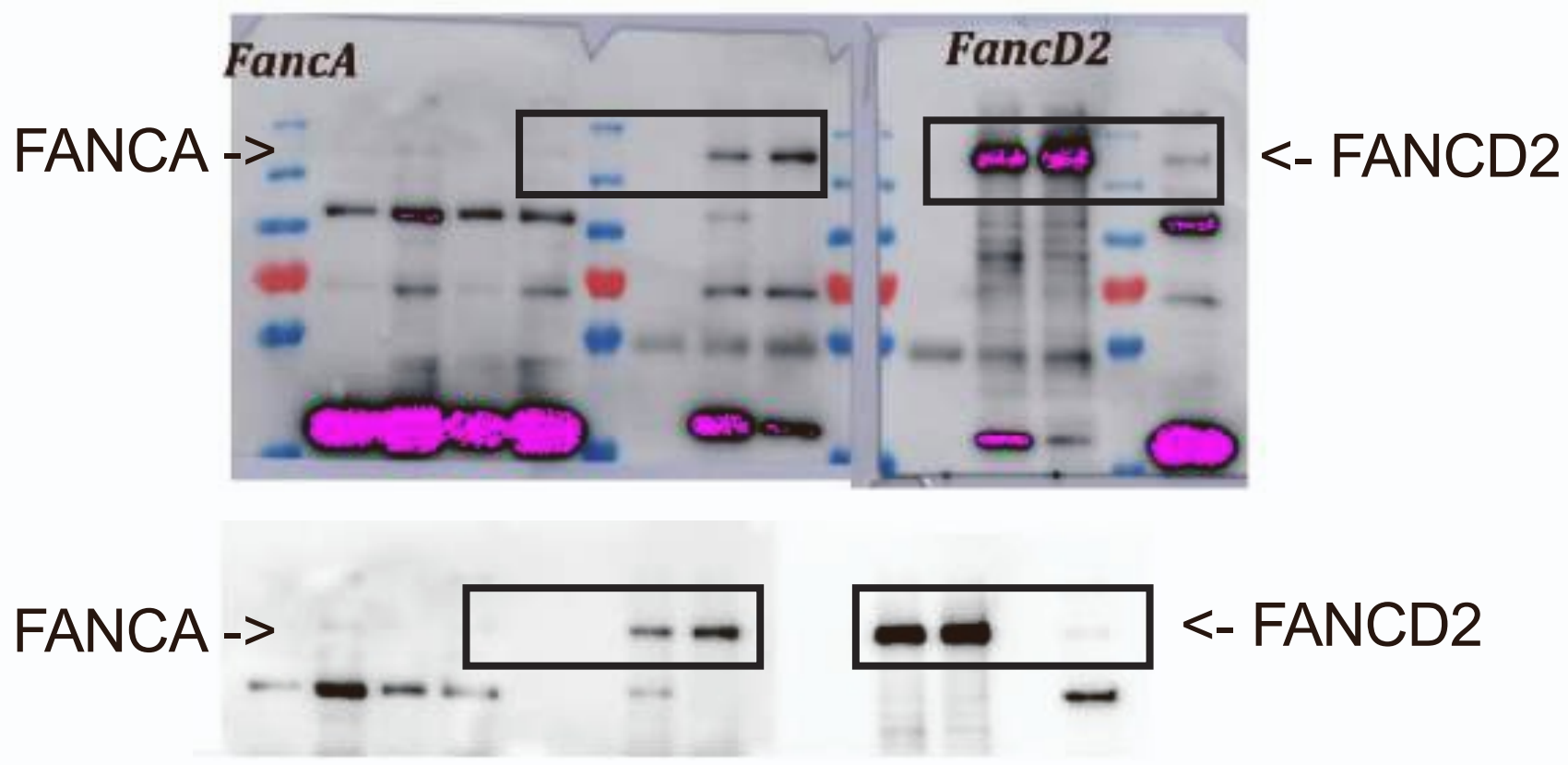

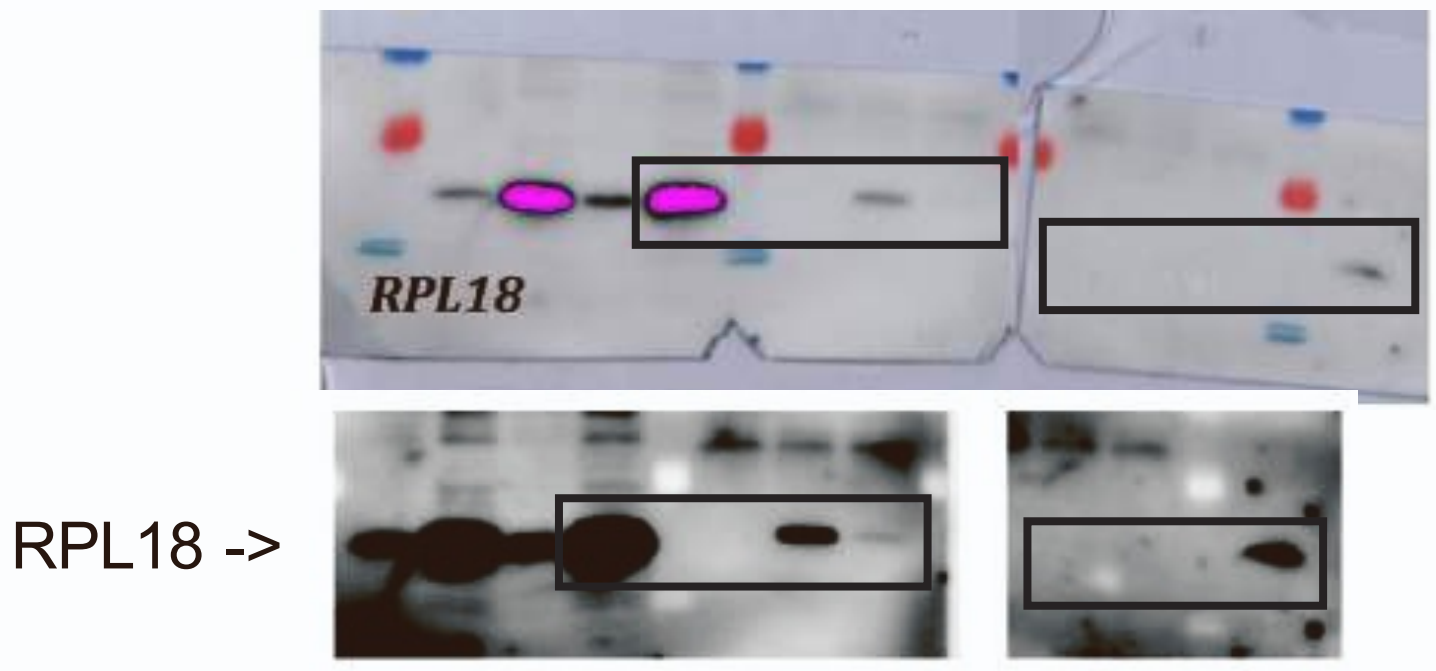

For FIGURE 2I

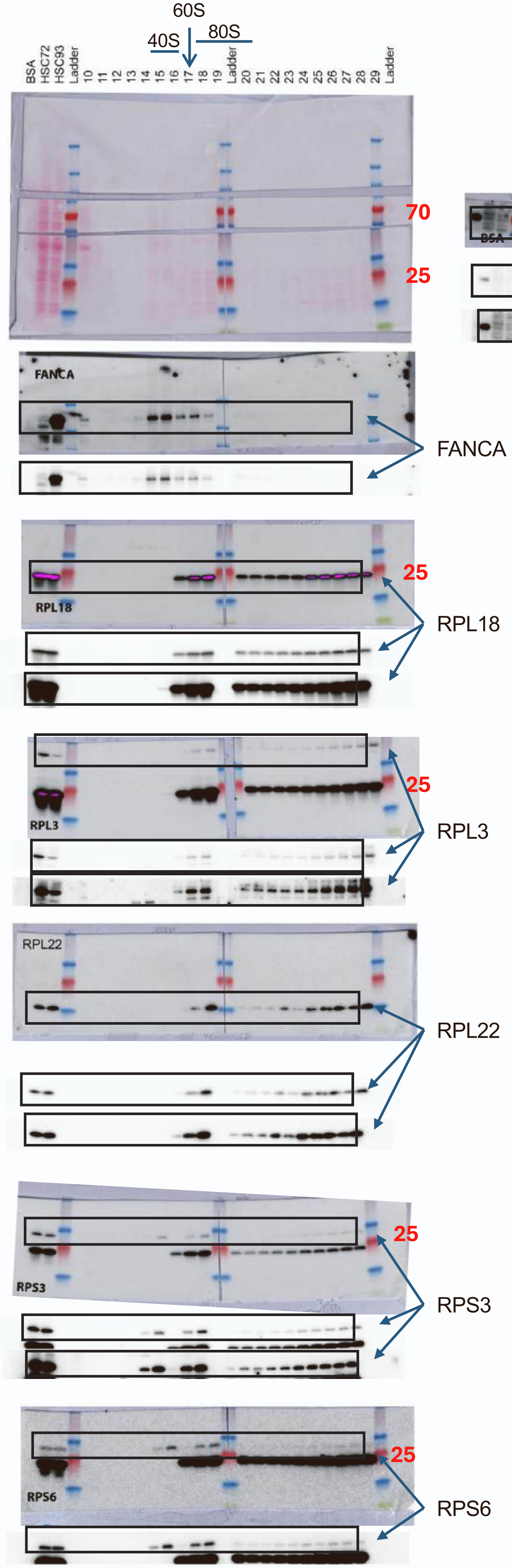

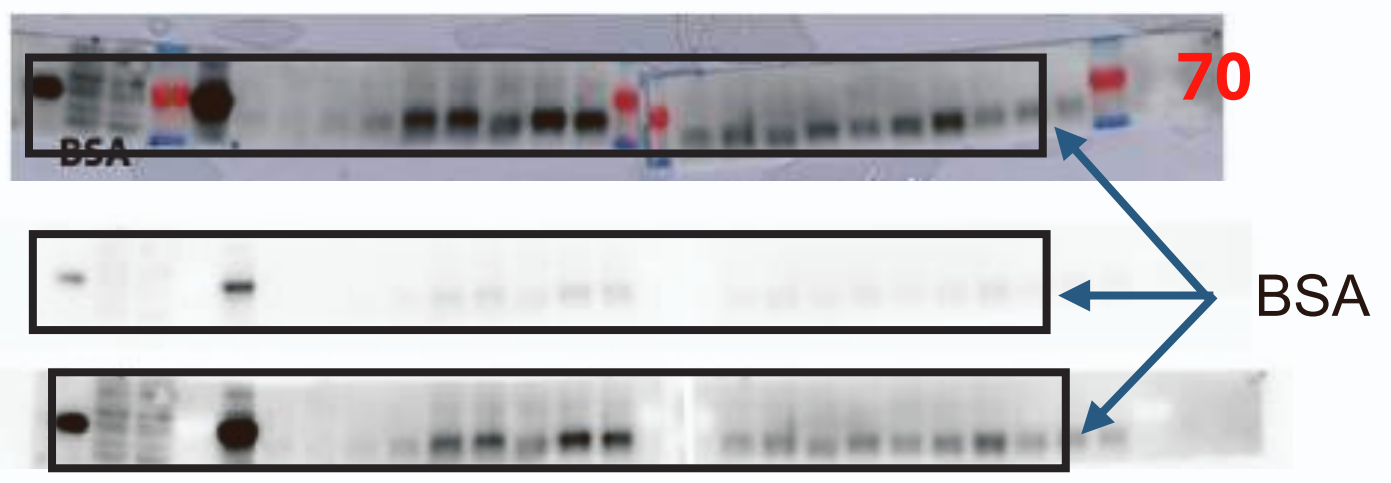

The original membrane was cut into three parts:
The upper part (>100 kDa) for FANCA;
The central part (between 55 kDa and 100 kDa) for BSA;
The lower part (<55 kDa) for the analyzed RPL and RPS proteins.

Two or three different exposure times are shown for each protein. The region chosen for the main figure is boxed.

The vertical cut was performed to allow hybridization in a suitable box or tube, limiting the amount of buffer containing the antibody. The two parts of the membrane were processed together for hybridization, washing, and detection in the Imager.

Approximate MW in kDa:

FANCA	160
BSA	66
RPL3	43
RPL18	22
RPL22	15
RPS3	28
RPS6	32

For FIGURE 7B

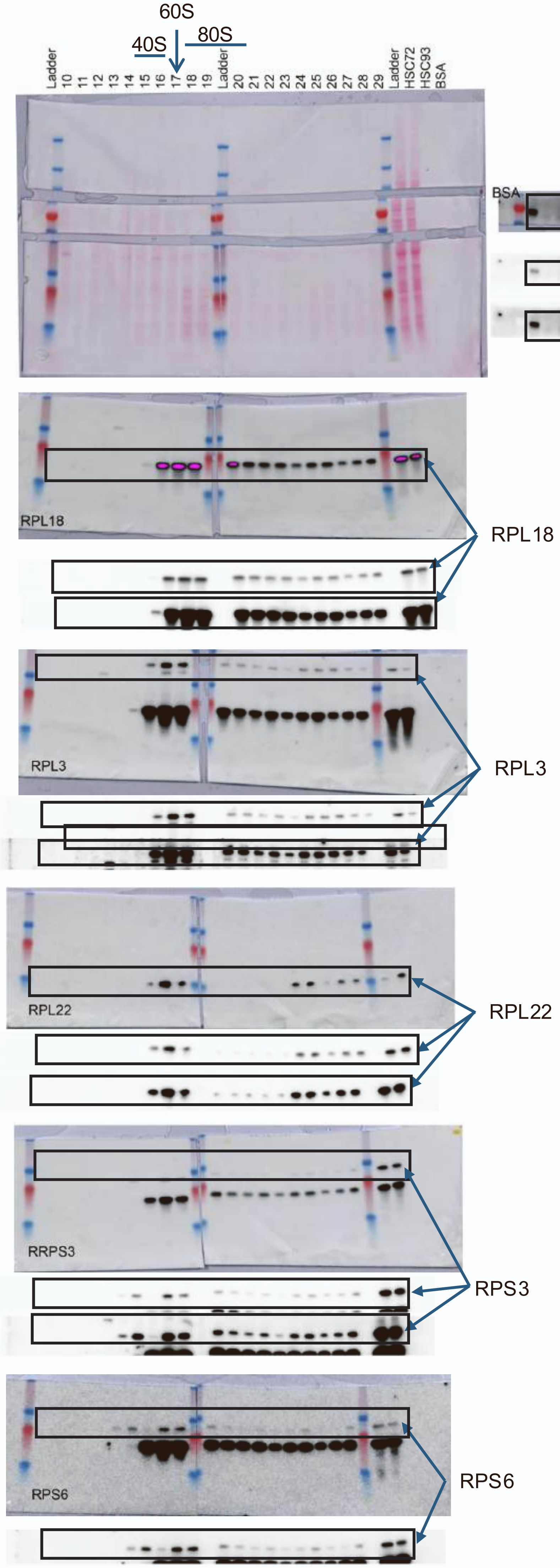

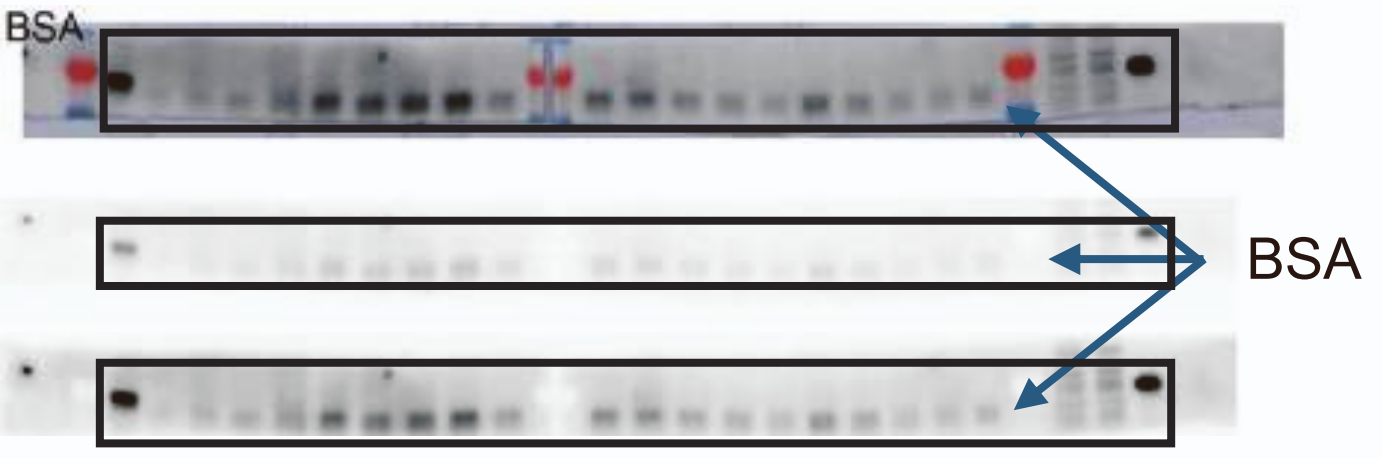

The original membrane was cut into three parts, as previously:
The upper part (>100 kDa) ;
The central part (between 55 kDa and 100 kDa) for BSA;
The lower part (<55 kDa) for the analyzed RPL and RPS proteins.

The upper portion was not used for hybridization with FANCA since, as can be observed in Figure 7B and as widely reported in the literature, the HSC72 cell line derived from FA patients does not express a full-length FANCA.

Different exposure times are shown for each protein. The region chosen for the main figure is boxed.

The vertical cut was performed to allow hybridization in a suitable box or tube, limiting the amount of buffer containing the antibody. The two parts of the membrane were processed together for hybridization, washing, and detection in the Imager.

Approximate MW in kDa:	
FANCA	160
BSA	66
RPL3	43
RPL18	22
RPL22	15
RPS3	28
RPS6	32

For FIGURE 7C
